# Vitamin D and mortality: Individual participant data meta-analysis of standardized 25-hydroxyvitamin D in 26916 individuals from a European consortium

**DOI:** 10.1371/journal.pone.0170791

**Published:** 2017-02-16

**Authors:** Martin Gaksch, Rolf Jorde, Guri Grimnes, Ragnar Joakimsen, Henrik Schirmer, Tom Wilsgaard, Ellisiv B. Mathiesen, Inger Njølstad, Maja-Lisa Løchen, Winfried März, Marcus E. Kleber, Andreas Tomaschitz, Martin Grübler, Gudny Eiriksdottir, Elias F. Gudmundsson, Tamara B. Harris, Mary F. Cotch, Thor Aspelund, Vilmundur Gudnason, Femke Rutters, Joline W. J. Beulens, Esther van ‘t Riet, Giel Nijpels, Jacqueline M. Dekker, Diana Grove-Laugesen, Lars Rejnmark, Markus A. Busch, Gert B. M. Mensink, Christa Scheidt-Nave, Michael Thamm, Karin M. A. Swart, Ingeborg A. Brouwer, Paul Lips, Natasja M. van Schoor, Christopher T. Sempos, Ramón A. Durazo-Arvizu, Zuzana Škrabáková, Kirsten G. Dowling, Kevin D. Cashman, Mairead Kiely, Stefan Pilz

**Affiliations:** 1 Department of Internal Medicine, Division of Endocrinology and Diabetology, Medical University of Graz, Graz, Austria; 2 Tromsø Endocrine Research Group, Department of Clinical Medicine, University of Tromsø, Tromsø, Norway; 3 Tromsø Cardiovascular Research Group UNN, Department of Clinical Medicine, UiT The Arctic University of Norway, Tromsø, Norway; 4 Department of Community Medicine, UiT The Arctic University of Norway, Tromsø, Norway; 5 Mannheim Institute of Public Health, Social and Preventive Medicine, Mannheim Medical Faculty, University of Heidelberg, Mannheim, Germany; 6 Synlab Academy, Mannheim, Germany; 7 Clinical Institute of Medical and Chemical Laboratory Diagnostics, Medical University of Graz, Graz, Austria; 8 Department of Internal Medicine II - Cardiology, University of Ulm Medical Centre, Ulm, Germany; 9 Department of Cardiology, Medical University of Graz, Graz, Austria; 10 Specialist Clinic for Rehabilitation Bad Aussee, Bad Aussee, Austria; 11 Department of Internal Medicine - Cardiology, Charité University Hospital Berlin, Campus Virchow Klinikum, Berlin, Germany; 12 Swiss Cardiovascular Center Bern, Department of Cardiology, Bern University Hospital, Bern, Switzerland; 13 Icelandic Heart Association, Kopavogur, Iceland; 14 National Institute on Aging, Laboratory of Epidemiology, and Population Sciences, Bethesda, Maryland, United States of America; 15 Division of Epidemiology and Clinical Applications, National Eye Institute, Bethesda, Maryland, United States of America; 16 Faculty of Medicine, School of Health Sciences, University of Iceland, Reykjavik, Iceland; 17 Department of Epidemiology and Biostatistics and EMGO+ Institute for Health and Care Research, VU University Medical Centre, Amsterdam, the Netherlands; 18 Julius Center for Health Sciences and Primary Care, University Medical Center Utrecht, Utrecht, The Netherlands; 19 Department of Endocrinology and Internal Medicine, Aarhus University Hospital, Aarhus, Denmark; 20 Department of Epidemiology and Health Monitoring, Robert Koch Institute, Berlin, Germany; 21 Department of Health Sciences and the EMGO Institute for Health and Care Research, Faculty of Earth and Life Sciences, VU University Amsterdam, Amsterdam, The Netherlands; 22 Department of Internal Medicine, Endocrine Section, VU University medical center, Amsterdam, the Netherlands; 23 National Institute of Health Office of Dietary Supplements, Bethesda, Maryland, United States of America; 24 Department of Preventive Medicine and Epidemiology, Loyola University Medical Center, Maywood, Illinois, United States of America; 25 Cork Centre for Vitamin D and Nutrition Research, School of Food and Nutritional Sciences, University College Cork, Cork, Ireland; 26 Department of Medicine, University College Cork, Cork, Ireland; 27 Irish Centre for Fetal and Neonatal Translational Research [INFANT], University College Cork, Cork, Ireland; Medical University of Gdańsk, POLAND

## Abstract

**Background:**

Vitamin D deficiency may be a risk factor for mortality but previous meta-analyses lacked standardization of laboratory methods for 25-hydroxyvitamin D (25[OH]D) concentrations and used aggregate data instead of individual participant data (IPD). We therefore performed an IPD meta-analysis on the association between standardized serum 25(OH)D and mortality.

**Methods:**

In a European consortium of eight prospective studies, including seven general population cohorts, we used the Vitamin D Standardization Program (VDSP) protocols to standardize 25(OH)D data. Meta-analyses using a one step procedure on IPD were performed to study associations of 25(OH)D with all-cause mortality as the primary outcome, and with cardiovascular and cancer mortality as secondary outcomes. This meta-analysis is registered at ClinicalTrials.gov, number NCT02438488.

**Findings:**

We analysed 26916 study participants (median age 61.6 years, 58% females) with a median 25(OH)D concentration of 53.8 nmol/L. During a median follow-up time of 10.5 years, 6802 persons died. Compared to participants with 25(OH)D concentrations of 75 to 99.99 nmol/L, the adjusted hazard ratios (with 95% confidence interval) for mortality in the 25(OH)D groups with 40 to 49.99, 30 to 39.99, and <30 nmol/L were 1.15 (1.00–1.29), 1.33 (1.16–1.51), and 1.67 (1.44–1.89), respectively. We observed similar results for cardiovascular mortality, but there was no significant linear association between 25(OH)D and cancer mortality. There was also no significantly increased mortality risk at high 25(OH)D levels up to 125 nmol/L.

**Interpretation:**

In the first IPD meta-analysis using standardized measurements of 25(OH)D we observed an association between low 25(OH)D and increased risk of all-cause mortality. It is of public health interest to evaluate whether treatment of vitamin D deficiency prevents premature deaths.

## Introduction

Vitamin D is involved in the regulation of calcium homeostasis and exerts beneficial effects on skeletal health.[1] Serum concentrations of 25-hydroxyvitamin D (25[OH]D) are measured to assess vitamin D status, which is mainly determined by sunlight (ultraviolet-B [UV-B]) induced vitamin D production in the skin and, to a lesser extent, by dietary or supplemental vitamin D intake.[1] Low 25(OH)D status has emerged as a risk factor for various adverse health outcomes including mortality,[2] but there is controversy on the classification of 25(OH)D status and the shape of the association between 25(OH)D concentrations and mortality.[1–10]

Existing knowledge on 25(OH)D status and mortality has been limited by lack of standardized serum 25(OH)D data. Previous studies have shown that differences in assay and laboratory methods have a significant impact on the reported 25(OH)D concentrations and thus on the association between 25(OH)D and health outcomes.[11,12] Therefore, the Vitamin D Standardization Program (VDSP), a collaborative initiative led by the National Institutes of Health-Office of Dietary Supplements (NIH-ODS), has developed protocols for standardizing 25(OH)D data from current and previous surveys to the standardized concentrations as measured by the National Institute of Standards and Technology (NIST) and Ghent University reference measurement procedure (RMP).[13,14]

In this work, which is part of the EU-project ‘Food-based solutions for eradication of vitamin D deficiency and health promotion throughout the life cycle’ (ODIN), we aimed to address the knowledge gap on the association between standardized 25(OH)D concentrations and mortality in a collaborative meta-analysis using individual participant data (IPD) from eight prospective studies across Europe. We are the first to use a one step approach for such a meta-analysis which has the advantage that IPD from all studies are modelled simultaneously whereas conventional two step approaches are based on aggregate data, thereby combining measures of effect (e.g. risk ratios) from studies instead of using data of each individual for meta-analyses, which causes a significant loss of information, especially for mortality data.[15,16] Specifically, we studied associations of 25(OH)D with all-cause mortality as the primary outcome, and with cardiovascular and cancer mortality as secondary outcomes. Considering that the majority of the individual studies of this meta-analysis have already reported mortality data on original 25(OH)D concentrations, we present our results for standardized as well as for original 25(OH)D levels.[17]

## Methods

### Study identification and selection

We established a collaboration to undertake this meta-analysis of IPD. Potential partners in a work-package of the European Commission-funded ODIN project (www.odin-vitd.eu/) were invited to attend a one-day workshop in Amsterdam in November 2012 to discuss aims, implementation and development of the task. Invited European-based participants were mainly identified on the basis of having recently published data from large prospective cohorts on 25(OH)D and mortality. There were a number of prerequisites for inclusion of individual cohort studies in ODIN, such as availability of bio-banked samples and validated prospective data on clinical outcomes. This meta-analysis is registered at ClinicalTrials.gov, number NCT02438488 and adheres to recommendations for the rationale, conduct, and reporting of IPD meta-analysis.[15]

### Studies and participants

We used IPD from eight independent prospective cohort studies from Norway, Germany, Iceland, Denmark, and the Netherlands which included the 4^th^ survey of the Tromsø study, the Ludwigshafen Risk and Cardiovascular Health Study (LURIC), the Age, Gene/Environment Susceptibility-Reykjavik Study (AGES), the New Hoorn Study (NHS), the Aarhus Mammography Cohort Study, the German Health Interview and Examination Survey for Adults (DEGS) and the first and second cohort of the Longitudinal Aging Study Amsterdam (LASA). Within each cohort, we standardized cardiovascular and cancer end points on an individual basis, using definitions based on clinical and research expertise, availability of data, published guidelines and definitions, and the Standardized Data Collection for Cardiovascular Trials Initiative.[18] Definitions were sought to fit as closely as possible by each participating institution.

The present analysis was based on individuals with complete data on age, sex, body mass index (BMI), season of blood sampling, 25(OH)D concentration, vital status at follow-up and follow-up-time (which was at least one day). Participants with missing data were excluded from the analysis and we performed no data imputation. Detailed study descriptions, including all covariate definitions, are presented in the appendix in S1 File. All cohort studies were conducted in accordance with the declaration of Helsinki and written informed consent was obtained from all participants.

### Participant involvement

No participants were involved in setting the research question or the outcome measures, nor were they involved in developing plans for design, or implementation of the study. No participants were asked to advise on interpretation or writing up of results. Easily comprehensible parts of the results will be disseminated to the public at http://www.odin-vitd.eu/.

### Standardization of 25(OH)D

In line with the VDSP protocol we conducted a sampling procedure to select a subset of 100–150 bio-banked serum samples from each individual cohort study to re-analyze 25(OH)D using a standardized and CDC-certified liquid chromatography-tandem mass spectrometry (LC- MS/MS) method, traceable to the United States NIST and RMPs standards.[13,14] The re-analysed 25(OH)D values were used to develop master regression equations for each cohort study which were used to re-calibrate existing 25(OH)D measurements according to the RMPs.[13,14] The NHS cohort had no previous 25(OH)D measurements and was analysed in full.

### Grouping by 25(OH)D concentrations

The IPD population was divided into seven groups according to their baseline 25(OH)D measurement. Thresholds of 25(OH)D incorporating both Institute of Medicine[1] and Endocrine Society[3] recommendations were defined as follows: Severely vitamin D deficient (<30.00 nmol/L); two groups of participants at risk for inadequacy (from 30 to 39.99 nmol/L and 40 to 49.99 nmol/L); vitamin D sufficient (from 50 to 74.99 nmol/L); two groups of vitamin D levels which are sufficient although not consistently associated with increased benefit (from 75 to 99.99 nmol/L and 100 to 124.99 nmol/L); and high vitamin D concentrations (≥ 125 nmol/L) with possible reason for concern.[1] To convert nmol/L to ng/mL divide by 2.496.[1]

### Statistical analysis

Differences between original and standardized 25(OH)D concentrations were assessed by paired Student’s t-test. For comparisons of characteristics across vitamin D status groups, we used ANOVA for continuous and χ^2^ test for categorical data, as appropriate.

All-cause mortality was the primary outcome and was available in all participating cohort studies. All mortality analyses were performed for original and standardized 25(OH)D concentrations, for the whole IPD and for each single cohort study.

Hazard rates were estimated by a one-step meta-analysis model. In general, the one-step approach for IPD meta-analyses was shown to be the more concise approach for binary outcomes compared to the frequently used two-step procedure.[16] A one-step IPD procedure can be employed by means of a parametric (e.g. Weibull) survival model, which may be more flexible compared to a Cox model when analysing mortality data.[19,20] A Weibull distribution of survival function was used since it graphically and in likelihood ratio testing appeared to fit best the underlying data against exponential, log-logistic, and log-normal distributions. We used a mixed model implemented in SAS PROC NLMIXED procedure (SAS Institute Inc., 100 SAS Campus Drive, Cary, USA) with random effects to account for clustering across cohort studies.[21] The goodness of fit was examined by likelihood ratio test and plotting estimated versus observed survival curves.

For mortality analyses, 25(OH)D was modelled two ways: using a traditional categorical variable approach with seven groups and a restricted cubic splines approach.[7] The cubic splines approach was chosen to retain the continuous nature of 25(OH)D values and to obtain hazard ratios (HR) with 95% confidence intervals (CI) at the median value of each group. For HR computation we chose the 25(OH)D group with the lowest mortality risk of model 2 as the reference.[22,23] Cubic splines were also used to estimate the nadir of the mortality curve, i.e. the concentration of 25(OH)D with the lowest mortality risk.[7] For cubic splines five knots located at the fifth, 27.5^th^, 50^th^, 72.5^th^, and 95^th^ percentile of the 25(OH)D distribution were selected.[24]

Our outcome analyses were adjusted for risk factors of mortality and determinants of vitamin D status. In model 1 we adjusted for age (in years), sex (male/female), and season of blood collection (Spring, Summer, Autumn, and Winter). In model 2, our main statistical model, we additionally adjusted for BMI (in kg/m^2^). In model 3, we additionally adjusted for diabetes mellitus (yes/no) and arterial hypertension (yes/no), and in model 4 we added history of cancer (yes/no), history of cardiovascular disease (yes/no) and current smoking status (yes/no) as covariates.

Additional covariate adjustments had model 2 as reference model and included adjustments for supplemental intake of calcium, supplemental intake of vitamin D, physical activity, estimated glomerular filtration rate (eGFR), parathyroid hormone, C-reactive protein, systolic blood pressure, low density lipoprotein cholesterol, and glucose. Participants or cohorts with missing additional covariate data were excluded from the respective additional covariate analysis.

All models for all-cause and cause-specific mortality were computed using original 25(OH)D but without NHS, as NHS had no original 25(OH)D measurements. For standardized 25(OH)D, we computed all models first without data from NHS to provide comparable results between models of original and standardized 25(OH)D measurements and then with all data (including NHS).

#### Sensitivity analyses

Pre-specified subgroup analyses were performed to stratify for risk factors for vitamin D deficiency and mortality. Specifically, we stratified for sex (females/males), age groups (<60 yrs; 60 to <70 yrs; ≥70 yrs), BMI groups (<25 kg/m^2^; 25 to <30 kg/m^2^; ≥30 kg/m^2^), calcium supplementation (yes/no), vitamin D supplementation (yes/no), history of CVD (yes/no), and history of cancer (yes/no). Further sensitivity analyses were restricted to general population cohorts (i.e. all cohorts except LURIC) and to individuals that died > 1 year and > 3 years after baseline examination.

#### Secondary outcomes

Secondary outcomes were cardiovascular mortality and cancer mortality, and were available in all cohort studies except NHS. We utilized traditional Cox proportional hazards and the modified risks regression according to the method of Fine and Gray to account for competing risks.[25]

#### Heterogeneity

To assess the size of the random effect as well as the heterogeneity across studies, we calculated an intra-class correlation coefficient (ICC) and its 95% CI. This coefficient can be interpreted as the I^2^ measure of inconsistency proposed by Higgins and colleagues, whereby 25% represents small heterogeneity, 50% represents medium heterogeneity, and 75% represents large heterogeneity.[26]

#### Macros

For the restricted cubic splines in regression models, we used a SAS macro provided by Harrell FE at the Department of Biostatistics, Vanderbilt University School of Medicine, Nashville, TN, USA.[27] The competing risk analyses were performed using R and ‘crrSC’ package, Version 1.1 (2013-06-23; Bingqing Zhou and Aurelien Latouche), which is an extension of the ‘cmprsk’ package, Version 2.2–7 (2014-06-17; Bob Gray) to stratified and clustered data.[28]

All statistical tests were two sided using an α level of 0.05. Analyses of all-cause mortality were conducted with SAS Version 9.2 (SAS Institute Inc., 100 SAS Campus Drive, Cary, USA), analyses of cause-specific mortality were run by R Version 3.1.1 (2014-07-10; Copyright © The R Foundation for Statistical Computing). Data pooling and management was performed with SPSS version 20 (IBM Corp. Released 2011. IBM SPSS Statistics for Windows, NY) or MS Excel (Microsoft Excel. Redmond, Washington, USA). Additional details on the statistical analyses can be found in the appendix in S1 File.

## Results

Baseline characteristics of the entire study population and of all eight individual cohort studies comprising 26916 participants are shown in Table 1.

**Table 1:** Baseline characteristics of the entire study population and the individual cohort studies.

Characteristic	Total cohort (N = 26916)	Tromsø (N = 7145) NORWAY	LURIC (N = 3299) GERMANY	AGES (N = 5510) ICELAND	NHS (N = 2591) NETHERLANDS	Aarhus (N = 2473) DENMARK	DEGS (N = 3862) GERMANY	LASA, first cohort (N = 1302) NETHERLANDS	LASA, second cohort (N = 734) NETHERLANDS
Age, median (IQR), years	61.6 (51.9–71.8)	60.1 (53.7–67.2)	64.5 (56.3–70.5)	76 (72–81)	54 (48–59)	50.5 (42.2–58.4)	43 (32–57)	75.1 (69.9–81.1)	60.2 (57.3–62.6)
Sex, % (women)	58	61	30	57	54	100	56	51	54
Season^a^, %									
Winter	27	32	23	26	26	26	16	29	51
Spring	27	35	20	26	15	21	28	22	38
Summer	16	4	25	15	26	24	20	23	10
Autumn	30	29	32	33	33	29	36	26	<1
BMI, median (IQR), kg/m²	25.9 (23.4–28.8)	25.5 (23.2–28.3)	27.1 (24.7–29.7)	26.7 (24.1–29.6)	25.6 (23.4–28.3)	23.1 (21.3–26)	25.9 (23.1–29.2)	26.5 (24.1–29.2)	26.8 (24.3–29.6)
Current smoking, %	24	33	20	12	21	23	32	18	27
Physical activity^b^, % (missing)	13	2	3	3	19	N.A.	<1	3	1
Low frequency, %	59	64	80	65	9	N.A.	55	61	43
Medium frequency, %	28	29	12	23	50	N.A.	31	34	47
High frequency, %	13	7	8	12	41	N.A.	14	5	10
Present Diabetes^c^, %	11	3	32	12	23	2	8	8	7
Glucose, median (IQR), mmol/L	5.4 (4.9–5.9)	N.A.	5.1 (4.6–5.9)	5.5 (5.2–6.0)	5.4 (4.5–6.6)	N.A.	5.2 (4.9–5.6)	5.6 (5.0–6.7)	4.8 (4.1–5.5)
Present HTN^d^, %	60	56	93	81	40	13	45	78	61
SBP, median (IQR), mmHg	138 (125–154)	140 (127–157)	123 (123–157)	140 (128–155)	131 (121–144)	N.A.	131 (129–145)	151 (134–169)	139 (126–154)
LDL-C, median (IQR), mmol/L	3.6 (2.9–4.4)	4.4 (3.6–5.2)	3.0 (2.4–3.8)	3.5 (2.8–4.2)	3.3 (2.7–3.9)	N.A.	3.6 (3.0–4.4)	3.6(3.1–4.3)	3.3 (2.8–4.0)
History of CVD^e^, %	14	7	46	19	N.A.	2	3	10	7
History of cancer, %	8	5	7	16	N.A.	7	3	12	8
eGFR, median (IQR), mL/min/1.73m²	83 (69–97)	97 (86–109)	81 (70–92)	67 (57–78)	N.A.	88 (78–98)	88 (78–98)	65 (57–73)	68 (61–76)
CRP, median (IQR), mg/dL	2.0 (0.9–4.4)	N.A.	2.7 (1.2–7.4)	1.9 (1.0–3.8)	N.A.	N.A.	1.4 (0.6–3.1)	3.2 (1.5–6.5)	N.A.
Calcium Supplements, %	7	7	1	17	N.A.	26	N.A.	11	N.A.
Vitamin D Supplements, %	21	40	1	79	N.A.	54	N.A.	N.A.	N.A.
PTH, median (IQR), pmol/L	3.4 (2.4–4.7)	2.5 (1.9–3.5)	3.1 (2.3–4.1)	4.5 (3.6–5.6)	N.A.	4.1 (3.2–5.3)	3.2 (1.8–4.6)	3.2 (2.5–4.3)	5.6 (4.3–7.0)

Baseline characteristics are presented as median with interquartile range or percentage where appropriate. Abbreviations: Tromsø = 4^th^ Tromsø Study; LURIC = Ludwigshafen RIsk and Cardiovascular Health Study; AGES = Age, Gene/Environment Susceptibility Reykjavik Study; NHS = New Hoorn Study; Aarhus = Aarhus Mammography Cohort Study; DEGS = German Health Interview and Examination Survey for Adults; LASA = The Longitudinal Aging Study Amsterdam; BMI = Body mass index; HTN = Arterial hypertension; SBP = Systolic blood pressure; LDL-C = Low density lipoprotein cholesterol; CVD = Cardiovascular disease; eGFR = Estimated glomerular filtration rate according to the four-variable Modification of Diet in Renal Disease (MDRD) formula; CRP = C-reactive protein; PTH = Parathyroid hormone; N.A. = Not available.

^a^Season of baseline blood sampling was defined as spring (March to May), summer (June to August), autumn (September to November), and winter (December to February).

^b^Physical activity was defined as frequency of medium- or vigorous leisure activity and was stratified in low (<1 hour per week), medium (1–3 hours) and high frequency (>3 hours per week).

^c^Diabetes mellitus at baseline was defined as (listed according to priority—highest priority first): Those participants on glucose lowering drugs, physician-reported, self-reported or according to ADA: fasting glucose ≥ 7.0 mmol/L, 2h postload glucose ≥ 11.1 mmol/L or HbA1c ≥ 6.5% (ICD-9: 250; ICD-10: E10-E14).

^d^Arterial hypertension at baseline was defined as (listed according to priority—highest priority first): Participants already on antihypertensive drug treatment, physician-reported, self-reported HTN, office systolic and/or diastolic blood pressure of equal to or higher than 140 and/or 90 mm Hg (ICD-9: 401,405; ICD-10: I10,I15).

^e^History of CVD at baseline was defined as positive history of myocardial infarction and/or stroke.

Table A in S1 File shows study specific details on 25(OH)D assays and data availability. Notably, we have already published data on the standardization procedure and the 25(OH)D distribution for all of our study cohorts, except of the LURIC study.[17] Distributions of participants across the 25(OH)D groups and differences between standardized and original 25(OH)D concentrations are shown in Table B in S1 File. Associations between standardized 25(OH)D concentrations and baseline variables are shown in Table C in S1 File. Several risk factors for mortality outcomes such as BMI, smoking, arterial hypertension, diabetes mellitus, C-reactive protein and low physical activity were associated with low 25(OH)D concentrations.

Descriptive mortality follow-up data are shown for the entire study population, as well as for each individual study in Table D in S1 File. Characteristics of survivors (n = 20114) compared to deceased study participants (n = 6802) are shown in Table E in S1 File.

**Fig 1:**
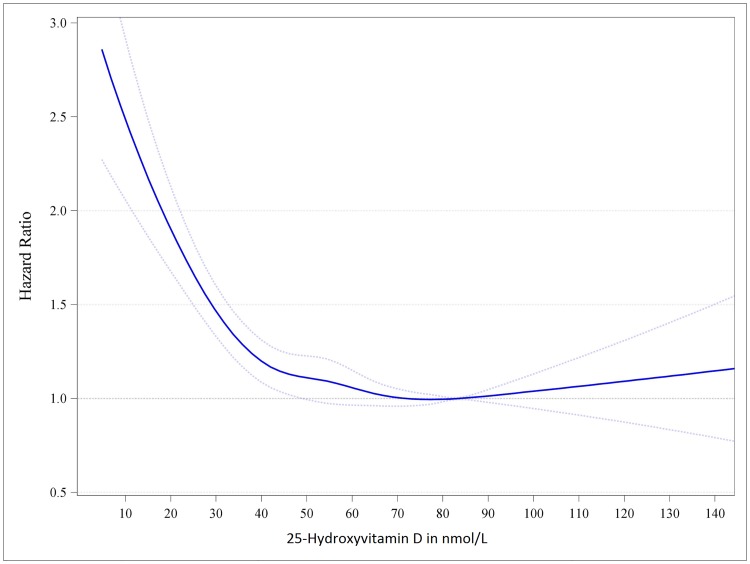
Dose-response trend of hazard ratios of death from all causes by standardized 25-hydroxyvitamin D. Dose-response trend of hazard ratios of all-cause mortality by standardized 25-hydroxyvitamin D were adjusted for age, sex, body mass index and season of blood drawing concentrations. Hazard ratios [blue line with 95% confidence interval as the dotted blue lines] are referring to the 25-hydroxyvitamin D concentration of 83.4 nmol/L (i.e. the median 25-hydroxyvitamin D concentration for the group with 25-hydroxyvitamin D concentrations from 75 to 99.99 nmol/L).

Results for the IPD meta-analysis on standardized 25(OH)D and total all-cause mortality are presented in Table 2, and the respective cubic splines regression curve is shown in Fig 1.

**Table 2:** Adjusted hazard ratio of death from all causes (95% CI) by standardized 25-hydroxyvitamin D concentrations in nmol/L and statistical approach for full database.

	Category	<30	30–39.99	40–49.99	50–74.99	75–99.99	100–124.99	≥125
	Median*, nmol/L	23.0	35.9	45.3	60.4	83.6	107.2	135.0
	Sample size, n	2951	3106	5018	11865	3125	679	172
	Deaths, n	999	892	1386	2935	522	57	11
Model 1^a^	Categorical HR (95% CI)	1.65 (1.43–1.87)	1.32 (1.14–1.49)	1.13 (0.99–1.28)	1.05 (0.93–1.16)	1.00	1.00 (0.66–1.33)	0.97 (0.27–1.67)
	Cubic-splines HR (95% CI)	1.75 (1.56–1.93)	1.28 (1.16–1.39)	1.13 (1.02–1.23)	1.05 (0.96–1.14)	1.00	1.06 (0.92–1.19)	1.14 (0.82–1.46)
	Nadir, nmol/L (95% CI)	77.6 (68.2–87.0)						
Model 2^b^	Categorical HR (95% CI)	1.67 (1.44–1.89)	1.33 (1.16–1.51)	1.15 (1.00–1.29)	1.05 (0.93–1.17)	1.00	1.00 (0.66–1.33)	0.98 (0.27–1.68)
	Cubic-splines HR (95% CI)	1.76 (1.58–1.95)	1.29 (1.17–1.41)	1.14 (1.03–1.24)	1.06 (0.96–1.15)	1.00	1.06 (0.92–1.19)	1.13 (0.81–1.45)
	Nadir, nmol/L (95% CI)	78.1 (67.9–88.3)						
Model 3^c^	Categorical HR (95% CI)	1.61 (1.39–1.83)	1.32 (1.14–1.50)	1.14 (1.00–1.29)	1.06 (0.94–1.18)	1.00	1.03 (0.68–1.37)	0.97 (0.26–1.69)
	Cubic-splines HR (95% CI)	1.72 (1.53–1.90)	1.27 (1.15–1.38)	1.12 (1.02–1.23)	1.05 (0.96–1.14)	1.00	1.06 (0.91–1.20)	1.15 (0.77–1.53)
	Nadir, nmol/L (95% CI)	77.7 (68.7–86.7)						
Model 4^d^	Categorical HR (95% CI)	1.50 (1.28–1.71)	1.24 (1.07–1.42)	1.12 (0.97–1.27)	1.05 (0.92–1.18)	1.00	1.07 (0.69–1.45)	0.87 (0.21–1.53)
	Cubic-splines HR (95% CI)	1.56 (1.38–1.74)	1.19 (1.08–1.31)	1.08 (0.97–1.19)	1.04 (0.95–1.12)	1.00	1.04 (0.89–1.19)	1.10 (0.72–1.49)
	Nadir, nmol/L (95% CI)	78.6 (69.3–88.0)						

Statistical approach was based on 1) categorical models and 2) cubic splines models. Estimates of the cubic splines approach were calculated for the (*) median 25-hydroxyvitamin D value of each category. Categories are based on the Institute of Medicine report 2011 used cut-off values. Abbreviations: HR = Hazard ratio with 95% confidence interval (CI). The nadir is the level of 25-hydroxyvitamin D with the lowest predicted risk.

^a^Adjusted for age, sex, and season of blood drawing at baseline visit.

^b^Adjusted for age, sex, season of blood drawing, and body mass index (BMI) at baseline visit.

^c^Adjusted for age, sex, season of blood drawing, BMI, diabetes mellitus, and arterial hypertension at baseline visit.

^d^Adjusted for age, sex, season of blood drawing, BMI, active smoker status, diabetes mellitus, arterial hypertension, history of cardiovascular disease (CVD), and history of cancer at baseline visit. History of CVD was defined as history of myocardial infarction and/or history of stroke. History of CVD and history of cancer were not available in the New Hoorn Study (NHS) therefore Model 4 was conducted without NHS.

Risk for all-cause mortality did not significantly differ by 25(OH)D concentration for values ranging from 50 to 125 nmol/L and higher. The lowest mortality risk was detected for 25(OH)D concentrations of approximately 78 nmol/L.[1,3] Compared to the reference group with 25(OH)D concentrations from 75 to 99.99 nmol/L, all-cause mortality was marginally increased in participants with 25(OH)D concentrations from 40 to 49.99 nmol/l and was significantly increased in the group with 25(OH)D concentrations from 30 to 39.99 nmol/L, and even more pronounced for 25(OH)D concentrations below 30 nmol/L. Results for mortality risk according to standardized 25(OH)D using additional adjustments are shown in Table F in S1 File.

For the seven studies with available original and standardized 25(OH)D concentrations we present data on overall mortality according to 25(OH)D values in Figure A and in Table G and Table H in S1 File. Individual study cohort results using standardized 25(OH)D appear in Tables I—P and in Figures B—F in S1 File. Sensitivity analyses for standardized 25(OH)D concentrations are shown in Table Q in S1 File.

There was a significant inverse association between standardized 25(OH)D concentrations and cardiovascular mortality (Table 3) while no linear association was found between categories of standardized 25(OH)D and cancer deaths (Table 4).

**Table 3:** Adjusted hazard ratio of death from cardiovascular causes (95% CI) by standardized 25-hydroxyvitamin D concentrations in nmol/L in competing risk analysis for full database without the New Hoorn Study.

	Category	<30	30–39.99	40–49.99	50–74.99	75–99.99	≥100
	Median*, nmol/L	22.8	35.9	45.3	60.2	83.7	110.3
	Sample size, n	2716	2853	4638	10717	2648	753
	Deaths, n	397	257	377	663	100	16
Model 1^a^	Categorical HR (95% CI)	3.18 (1.82–5.53)	1.99 (1.64–2.40)	1.72 (1.47–2.02)	1.35 (1.12–1.63)	1.00	0.95 (0.69–1.31)
Model 2^b^	Categorical HR (95% CI)	3.10 (1.78–5.41)	1.93 (1.60–2.33)	1.69 (1.47–1.95)	1.34 (1.12–1.61)	1.00	0.95 (0.69–1.31)
Model 3^c^	Categorical HR (95% CI)	2.54 (1.74–3.71)	1.74 (1.61–1.89)	1.68 (1.41–2.00)	1.38 (1.11–1.70)	1.00	0.91 (0.65–1.29)
Model 4^d^	Categorical HR (95% CI)	2.21 (1.50–3.26)	1.61 (1.46–1.77)	1.65 (1.39–1.97)	1.37 (1.12–1.67)	1.00	0.92 (0.62–1.36)

Categories are based on the Institute of Medicine report 2011 used cut-off values. Abbreviations: HR = Hazard ratio with 95% confidence interval (CI);

*The median is the median 25-hydroxyvitamin D value of each category.

^a^Adjusted for age, sex, and season of blood drawing at baseline visit.

^b^Adjusted for age, sex, season of blood drawing, and body mass index (BMI) at baseline visit.

^c^Adjusted for age, sex, season of blood drawing, BMI, diabetes mellitus, and arterial hypertension at baseline visit.

^d^Adjusted for age, sex, season of blood drawing, BMI, active smoker status, history of cardiovascular disease (CVD), and history of cancer at baseline visit. History of CVD was defined as history of myocardial infarction and/or history of stroke.

**Table 4:** Adjusted hazard ratio of death from cancer causes (95% CI) by standardized 25-hydroxyvitamin D concentrations in nmol/L in competing risk analysis for full database without the New Hoorn Study.

	Category	<30	30–39.99	40–49.99	50–74.99	75–99.99	≥100
	Median*, nmol/L	22.8	35.9	45.3	60.2	83.7	110.3
	Sample size, n	2716	2853	4638	10717	2648	753
	Deaths, n	152	158	313	670	105	16
Model 1^a^	Categorical HR (95% CI)	1.08 (0.74–1.60)	1.11 (0.91–1.35)	1.29 (1.12–1.49)	1.25 (1.07–1.47)	1.00	0.80 (0.60–1.06)
Model 2^b^	Categorical HR (95% CI)	1.10 (0.75–1.61)	1.13 (0.93–1.36)	1.30 (1.13–1.50)	1.26 (1.07–1.48)	1.00	0.80 (0.60–1.07)
Model 3^c^	Categorical HR (95% CI)	1.15 (0.78–1.69)	1.16 (0.94–1.42)	1.33 (1.14–1.55)	1.26 (1.07–1.49)	1.00	0.82 (0.61–1.10)
Model 4^d^	Categorical HR (95% CI)	1.08 (0.73–1.60)	1.07 (0.87–1.32)	1.25 (1.07–1.46)	1.24 (1.06–1.45)	1.00	0.79 (0.60–1.04)

Categories are based on the Institute of Medicine report 2011 used cut-off values. Abbreviations: HR = Hazard ratio with 95% confidence interval (CI);

*The median is the median 25-hydroxyvitamin D value of each category.

^a^Adjusted for age, sex, and season of blood drawing at baseline visit.

^b^Adjusted for age, sex, season of blood drawing, and body mass index (BMI) at baseline visit.

^c^Adjusted for age, sex, season of blood drawing, BMI, diabetes mellitus, and arterial hypertension at baseline visit.

^d^Adjusted for age, sex, season of blood drawing, BMI, active smoker status, history of cardiovascular disease (CVD), and history of cancer at baseline visit. History of CVD was defined as history of myocardial infarction and/or history of stroke.

For all of our meta-analyses the I^2^ measure of inconsistency was below 25%, thus indicating small heterogeneity. The Preferred Reporting Items for Systematic Review and Meta-Analyses of individual participant data (PRISMA-IPD) checklist is included in the appendix (S1 File).

## Discussion

In this IPD meta-analysis of a Northern European consortium, we have shown that low standardized 25(OH)D concentrations are associated with an increased risk of all-cause and cardiovascular mortality, but not with cancer mortality.

Our results are, in general, in line with findings from previous studies, but our work is the first meta-analysis using standardized 25(OH)D data and a one-step procedure for statistical analyses, in which data from each individual participant was used to calculate the regression curve for the 25(OH)D and mortality relationship.[4–10] The shape of this mortality curve has, in our opinion, important clinical implications that significantly add to the existing literature (Fig 1). We acknowledge that there was limited statistical power to evaluate the association between 25(OH)D and mortality for the small number of individuals with 25(OH)D concentrations higher than 125 nmol/L (n = 172). Nevertheless, in that group, there was no apparent excess of mortality and thus no clear indication of vitamin D toxicity leading to reduced survival. Some other investigations have reported on increased mortality at very high 25(OH)D levels, and it has been hypothesized that these findings may have been driven by individuals with particular high 25(OH)D concentrations who started supplementing vitamin D because they were previously vitamin D deficient.[29,30] While there is an ongoing debate on whether 50 and/or 75 nmol/L should be used as a threshold for 25(OH)D classification of sufficiency,[1,3] we observed no meaningful variation of all-cause mortality risk for 25(OH)D concentrations ranging from 50 to 125 nmol/L and higher. Compared to the reference group, there was a marginally increased risk of mortality in participants with 25(OH)D concentrations from 40 to 49.99 nmol/L, but statistical significance was lost in the majority of the multivariate adjusted models (Table F in S1 File). A consistent and steep increase in mortality was, however, observed in participants with 25(OH)D concentrations below 40 nmol/L. It is, thus, of concern that the prevalence of standardized 25(OH)D concentrations below 40 nmol/L was 22.5% in all cohort studies and 18.3% in the seven population-based studies. In this context, it should also be noted that even relatively small differences in mortality risk may be of clinical significance on a population level.

With regard to our secondary outcomes, we observed that vitamin D deficient individuals were at significantly increased risk of cardiovascular mortality, whereas there was no clear linear relationship between 25(OH)D and cancer mortality. Season and follow-up time did not have a significant effect on the association between 25(OH)D and cancer mortality (data not shown).[31–33] Furthermore, the association between 25(OH)D and cancer-specific mortality may be different in secondary compared to primary prevention cohorts [6]. Our findings on cause-specific mortality may, however, suggest that the association between 25(OH)D and mortality is significantly driven by cardiovascular rather than cancer deaths, but general limitations regarding the classification and analyses of specific causes of death should be considered.[19,34]

We did not observe major differences between original and standardized 25(OH)D concentrations, but a few percent of the participants, which is relevant from a public health perspective, were indeed re-classified into different 25(OH)D groups after the standardization procedure (Table B in S1 File). While there were thus no dramatic changes in mean 25(OH)D concentrations, as expected, it should be noted that previous investigations reported on significant assay and laboratory differences in 25(OH)D concentrations.[11–14,27] This underlines the importance of using standardized 25(OH)D concentrations, and caution is warranted when interpreting results based on 25(OH)D data (even from LC-MS/MS methods) that have not been standardized to the concentrations as measured by the NIST and Ghent RMPs.[11–14]

The association between 25(OH)D and mortality could be causal because the vitamin D receptor (VDR) is expressed in almost all human tissues and activation of this receptor regulates hundreds of genes with relevance for several diseases.[2–5] Alternatively, our results could be explained by reverse causation, i.e. that 25(OH)D is reduced due to underlying diseases, or by confounding. Potential confounding factors such as inflammation, obesity, low physical activity and poor nutrition are associated with both vitamin D deficiency and increased mortality. In the present meta-analysis, the association between low 25(OH)D and mortality remained, however, significant despite careful statistical adjustments, but we cannot exclude residual confounding. Moreover, our findings remained materially unchanged in sensitivity analyses of individuals who died at least three years after baseline examination, which argues against, but does not exclude, reverse causation. When addressing the question of causality, it should also be acknowledged that most findings from observational studies on the associations between vitamin D deficiency and increased risk of chronic diseases such as cancer or cardiovascular diseases could not be confirmed as causal vitamin D effects in randomized controlled trials (RCTs).[4,5] It should, however, be underlined that meta-analyses of RCTs suggest that vitamin D supplementation may moderately, yet statistically significantly, reduce mortality.[6,34–37] Putting these results into perspective with our IPD meta-analysis, there exists, in our opinion, an urgent need to perform vitamin D RCTs in individuals with initial “standardized” 25(OH)D concentrations below 40 nmol/L, or, even better, below 30 nmol/L which represents 13% of the European population and to achieve optimal 25(OH)D levels.[17,38] It is therefore of concern that none of the large ongoing vitamin D RCTs has targeted such study populations.[37–39] In the event that these RCTs will not show significant survival benefits of vitamin D, we should not draw the final conclusion that vitamin D has no effect unless we have sufficient RCT data in participants with 25(OH)D levels below 40 or 30 nmol/L. Results from subgroup analyses of RCTs in participants with severe vitamin D deficiency, even if showing survival benefits of vitamin D in pre-specified analyses, will likewise not change clinical practice.[37–40]

A drawback of our work is that our data are based on a selection of European cohort studies, albeit representing data from almost 27000 individuals. Our data are also limited by significantly different results in the individual studies and by the fact that our data were restricted to high income countries. Therefore, the generalisability of our findings to other populations is somewhat limited. Moreover, the observational nature of our work precludes final conclusions regarding causality. Our analyses were, however, adjusted for a panel of potential confounders and our findings remained materially unchanged in several sensitivity analyses. Main strengths of our meta-analysis are the use of standardized 25(OH)D, the inclusion of previously unpublished study data on 25(OH)D status and mortality (e.g. first 25[OH]D mortality data of the NHS and 10-year follow-up data of the LURIC study) and the IPD meta-analysis approach with data harmonization.

In conclusion, we have shown, using standardized methods to assess 25(OH)D, that individuals at the lower end of the 25(OH)D distribution are at significantly increased risk of mortality, whereas for the majority of the population there is no consistent association between 25(OH)D status and mortality. This finding could be an explanation why available data on vitamin D supplementation and mortality are inconsistent because previous RCTs have, by the majority, enrolled participants regardless of their prevailing 25(OH)D status. Based on our results, we believe that the final answer on potential survival benefits of vitamin D should be derived from RCTs in severely vitamin D deficient individuals. These RCTs are urgently needed because vitamin D deficiency and its diagnosis and treatment are important public health issues.[1–4,17,39,40]

## Supporting information

S1 File Study details and detailed results. (DOC)

S1 Data File Individual participant data. (XLSX)

## Abbreviations

25(OH)D: 25-hydroxyvitamin D
AGES: Age, Gene/Environment Susceptibility-Reykjavik Study
ANOVA: analysis of variance
BMI: body mass index
CDC: Centres for Disease Control and Prevention
CVD: cardiovascular disease
DEGS: German Health Interview and Examination Survey for Adults
eGFR: estimated glomerular filtration rate
ICC: intra-class correlation coefficient
IOM: Institute of Medicine
IPD: individual participant data
LASA: Longitudinal Aging Study Amsterdam
LURIC: Ludwigshafen Risk and Cardiovascular Health Study
NHS: New Hoorn Study
NIH-ODS: National Institutes of Health-Office of Dietary Supplements
NIST: National Institute of Standards and Technology
ODIN: ‘Food-based solutions for eradication of vitamin D deficiency and health promotion throughout the life cycle’
RCT: randomized controlled trials
RMP: the NIST and Ghent University reference measurement procedure
UV-B: ultraviolet-B
VDSP: Vitamin D Standardization Program

## References

[1] Ross AC, Manson JE, Abrams SA, Aloia JF, Brannon PM, Clinton SK, et al The 2011 report on dietary reference intakes for calcium and vitamin D from the Institute of Medicine: what clinicians need to know. *J Clin Endocrinol Metab*. 2011;96(1):53–8. 10.1210/jc.2010-2704 21118827 PMC3046611

[2] Pludowski P, Holick MF, Pilz S, Wagner CL, Hollis BW, Grant WB, et al Vitamin D effects on musculoskeletal health, immunity, autoimmunity, cardiovascular disease, cancer, fertility, pregnancy, dementia and mortality-a review of recent evidence. *Autoimmun Rev*. 2013 8;12(10):976–89. 10.1016/j.autrev.2013.02.004 23542507

[3] Holick MF, Binkley NC, Bischoff-Ferrari HA, Gordon CM, Hanley DA, Heaney RP, et al Evaluation, treatment, and prevention of vitamin D deficiency: an Endocrine Society clinical practice guideline. *J Clin Endocrinol Metab*. 2011;96(7):1911–30. 10.1210/jc.2011-0385 21646368

[4] Autier P, Boniol M, Pizot C, Mullie P. Vitamin D status and ill health: a systematic review. *Lancet Diabetes Endocrinol*. 2014;2(1):76–89. 10.1016/S2213-8587(13)70165-7 24622671

[5] Theodoratou E, Tzoulaki I, Zgaga L, Ioannidis JP. Vitamin D and multiple health outcomes: umbrella review of systematic reviews and meta-analyses of observational studies and randomised trials. *BMJ*. 2014;348:g2035 10.1136/bmj.g2035 24690624 PMC3972415

[6] Chowdhury R, Kunutsor S, Vitezova A, Oliver-Williams C, Chowdhury S, Kiefte-de-Jong JC, et al Vitamin D and risk of cause specific death: systematic review and meta-analysis of observational cohort and randomised intervention studies. *BMJ*. 2014;348:g1903 10.1136/bmj.g1903 24690623 PMC3972416

[7] Sempos CT, Durazo-Arvizu RA, Dawson-Hughes B, Yetley EA, Looker AC, Schleicher RL, et al Is there a reverse J-shaped association between 25-hydroxyvitamin D and all-cause mortality? Results from the U.S. nationally representative NHANES. *J Clin Endocrinol Metab*. 2013;98(7):3001–9. 10.1210/jc.2013-1333 23666975 PMC3701270

[8] Schöttker B, Jorde R, Peasey A, Thorand B, Jansen EH, Groot Ld, et al Vitamin D and mortality: meta-analysis of individual participant data from a large consortium of cohort studies from Europe and the United States. *BMJ*. 2014;348:g3656 10.1136/bmj.g3656 24938302 PMC4061380

[9] Zittermann A, Iodice S, Pilz S, Grant WB, Bagnardi V, Gandini S. Vitamin D deficiency and mortality risk in the general population: a meta-analysis of prospective cohort studies. *Am J Clin Nutr*. 2012;95(1):91–100. 10.3945/ajcn.111.014779 22170374

[10] Garland CF, Kim JJ, Mohr SB, Gorham ED, Grant WB, Giovannucci EL, et al Meta-analysis of all-cause mortality according to serum 25-hydroxyvitamin D. *Am J Pub Health*. 2014;104(8):e43–50. 10.2105/AJPH.2014.302034 PMC4103214 24922127

[11] Binkley N, Krueger D, Cowgill CS, Plum L, Lake E, Hansen KE, et al Assay variation confounds the diagnosis of hypovitaminosis D: a call for standardization. *J Clin Endocrinol Metab*. 2004;89(7):3152–7. 10.1210/jc.2003-031979 15240586

[12] Schöttker B, Jansen EH, Haug U, Schomburg L, Köhrle J, Brenner H. Standardization of misleading immunoassay based 25-hydroxyvitamin D levels with liquid chromatography tandem-mass spectrometry in a large cohort study. *PLoS One*. 2012;7(11):e48774 10.1371/journal.pone.0048774 23133659 PMC3486791

[13] Binkley N, Sempos CT. Vitamin D Standardization Program (VDSP). Standardizing vitamin D assays: the way forward. *J Bone Miner Res*. 2014;29(8):1709–14. 10.1002/jbmr.2252 24737265 PMC5443565

[14] Cashman KD, Kiely M, Kinsella M, Durazo-Arvizu RA, Tian L, Zhang Y, et al Evaluation of Vitamin D Standardization Program protocols for standardizing serum 25-hydroxyvitamin D data: a case study of the program's potential for national nutrition and health surveys. *Am J Clin Nutr*. 2013;97(6):1235–42. 10.3945/ajcn.112.057182 23615829 PMC3652922

[15] Riley RD, Lambert PC, Abo-Zaid G. Meta-analysis of individual participant data: rationale, conduct, and reporting. *BMJ*. 2010;340:c221 10.1136/bmj.c221 20139215

[16] Debray TP, Moons KG, Abo-Zaid GM, Koffijberg H, Riley RD. Individual participant data meta-analysis for a binary outcome: one-stage or two-stage? *PLoS One*. 2013;8(4):e60650 10.1371/journal.pone.0060650 23585842 PMC3621872

[17] Cashman KD, Dowling KG, Škrabáková Z, Gonzalez-Gross M, Valtueña J, De Henauw S, et al Vitamin D deficiency in Europe: pandemic? *Am J Clin Nutr*. 2016 4;103(4):1033–44. 10.3945/ajcn.115.120873 26864360 PMC5527850

[18] Hicks KA, Hung HMJ, Mahaffey KW, Mehran R, Nissen SE, Stockbridge NL,et al. Standardized definitions for end point events in cardiovascular trials. (Accessed June 9th, 2014, at http://www.clinpage.com/images/uploads/endpoint-defs_11-16-2010.pdf).

[19] Riley RD. Commentary: like it and lump it? Meta-analysis using individual participant data. *Int J Epidemiol*. 2010;39(5):1359–61. 10.1093/ije/dyq129 20660642

[20] Royston P. Flexible parametric alternatives to the Cox model, and more. *Stata J*. 2001; 1:1–28.

[21] Abo-Zaid G, Guo B, Deeks JJ, Debray TP, Steyerberg EW, Moons KG, et al Individual participant data meta-analyses should not ignore clustering. *J Clin Epidemiol*. 2013;66(8):865–873. 10.1016/j.jclinepi.2012.12.017 23651765 PMC3717206

[22] Liu Q. A two-stage hierarchical regression model for meta-analysis of epidemiologic nonlinear dose-response data. *Comput Stat Data Anal*. 2009;53:4157–67.

[23] Bagnardi V. Flexible meta-regression functions for modeling aggregate dose-response data, with an application to alcohol and mortality. *Am J Epidemiol*. 2004;159:1077–86. 10.1093/aje/kwh142 15155292

[24] Harrell FE. Regression Modeling Strategies with Applications to Linear Models, Logistic egression and Survival Analysis. New York: Springer-Verlag 2001.

[25] Jason PF, Gray RJ. A proportional hazards model for the subdistribution of a competing risk. *J Am Stat Assoc*. 1999;94(446):496–509.

[26] Thomas D, Radji S, Benedetti A. Systematic review of methods for individual patient data meta- analysis with binary outcomes. *BMC Med Res Methodol*. 2014;14:79 10.1186/1471-2288-14-79 24943877 PMC4074845

[27] Harrell FE., Jr, DASPLINE Macro for SAS. (Accessed January 16th, 2015, at http://biostat.mc.vanderbilt.edu/twiki/pub/Main/SasMacros/survrisk.txt)

[28] Zhou B, Latouche A, Rocha V, Fine J. Competing risks regression for stratified data. *Biometrics*. 2011;67(2):661–70. 10.1111/j.1541-0420.2010.01493.x 21155744 PMC3431205

[29] Grant WB, Karras SN, Bischoff-Ferrari HA, Annweiler C, Boucher BJ, Juzeniene A, et al Do studies reporting ‘U’-shaped serum 25-hydroxyvitamin D–health outcome relationships reflect adverse effects? *Dermatoendocrinol*. 2016;8(1): e1187349 10.1080/19381980.2016.1187349 27489574 PMC4951179

[30] Kroll MH, Bi C, Garber CC, Kaufman HW, Liu D, Caston-Balderrama A, et al Temporal Relationship between Vitamin D Status and Parathyroid Hormone in the United States. *PLoS One*. 2015;10(3):e0118108 10.1371/journal.pone.0118108 25738588 PMC4349787

[31] Grant WB. Effect of interval between serum draw and follow-up period on relative risk of cancer incidence with respect to 25-hydroxyvitamin D level; implications for meta-analyses and setting vitamin D guidelines. *Dermatoendocrinol*. 2011;3(3):199–204. 10.4161/derm.3.3.15364 22110780 PMC3219171

[32] Grant WB. Effect of follow-up time on the relation between prediagnostic serum 25-hydroxyitamin D and all-cause mortality rate. *Dermatoendocrinol*. 2012;4(2):198–202. 10.4161/derm.20514 22928077 PMC3427200

[33] Eliassen AH, Warner ET, Rosner B, Collins LC, Beck AH, Quintana LM, et al Plasma 25-hydroxyvitamin D and risk of breast cancer in women followed over 20 years. *Cancer Res*. 2016; 76:5423–5430. 10.1158/0008-5472.CAN-16-0353 27530324 PMC5026605

[34] Bolland MJ, Grey A, Gamble GD, Reid IR. The effect of vitamin D supplementation on skeletal, vascular, or cancer outcomes: a trial sequential meta-analysis. *Lancet Diabetes Endocrinol*. 2014;2(4):307–20. 10.1016/S2213-8587(13)70212-2 24703049

[35] Bjelakovic G, Gluud LL, Nikolova D, Whitfield K, Wetterslev J, Simonetti RG, et al Vitamin D supplementation for prevention of mortality in adults. *Cochrane Database Syst Rev*. 2014;1:CD007470. 10.1002/14651858.CD007470.pub3 PMC11285307 24414552

[36] Rejnmark L, Avenell A, Masud T, Anderson F, Meyer HE, Sanders KM, et al Vitamin D with calcium reduces mortality: patient level pooled analysis of 70,528 patients from eight major vitamin D trials. *J Clin Endocrinol Metab*. 2012;97(8):2670–81. 10.1210/jc.2011-3328 22605432 PMC3410276

[37] Kupferschmidt K. Uncertain verdict as vitamin D goes on trial. *Science*. 2012;337(6101):1476–8.30. 10.1126/science.337.6101.1476 22997323

[38] Heaney RP. Guidelines for optimizing design and analysis of clinical studies of nutrient effects. *Nutr Rev*. 2014;72(1):48–54. 10.1111/nure.12090 24330136

[39] Pilz S, Rutters F, Dekker JM. Disease prevention: vitamin D trials. *Science*. 2012;338(6109):883. 10.1126/science.338.6109.883-c 23161977

[40] Amrein K, Schnedl C, Holl A, Riedl R, Christopher KB, Pachler C, et al Effect of high-dose vitamin D3 on hospital length of stay in critically ill patients with vitamin D deficiency: the VITdAL-ICU randomized clinical trial. *JAMA*. 2014;312(15):1520–30. 10.1001/jama.2014.13204 25268295

