# Supplementary material for: Vitamin D and mortality: Individual participant data meta-analysis of standardized 25-hydroxyvitamin D in 26916 individuals from a European consortium
Source: PLoS One. 2017 Feb 16;12(2):e0170791. doi: 10.1371/journal.pone.0170791 (PMC5312926; doi:10.1371/journal.pone.0170791)

**Vitamin D and mortality: Individual participant data meta-analysis of standardized 25-hydroxyvitamin D in 26916 individuals from a European consortium^1-33^**

**Appendix**

1. Study identification and selection

2. Study details and missing values

3. Construction of an individual participant database

4. Endpoint definitions

5. Covariate definitions

6. Covariate assessment

7. Re-analysis of bio-banked serum/plasma for total 25-hydroxyvitamin D by liquid chromatography-tandem mass spectrometry

8. Applying the VDSP protocol for standardization of serum 25(OH)D data from past surveys to the ODIN WP8 study populations

9. Statistical analyses

10. SAS code

11. Additional funding sources

12. Acknowledgements

13. Author's contribution

14. Prisma IPD list

15. References

16. Supplementary Figures

17. Supplementary Tables

18. PRISMA checklist

19. Meta-analysis flow chart

**1. Study identification and selection**

We established a collaboration to undertake this meta-analysis of individual patient data (IPD) of standardized 25-hydroxyvitamin D (25[OH]D) and mortality. Potential participants in a work-package (work package 8 [WP8]) of the European Commission-funded ODIN project ([www.**odin**-**vitd**.eu/](http://www.odin-vitd.eu/)) ‘Food-based solutions for eradication of vitamin D deficiency and health promotion throughout the life cycle’ were invited to attend a one-day workshop in Amsterdam in November 2012 to discuss aims, implementation and development of the task.^33^ Invited European-based participants were mainly identified on the basis of having recently published data from large prospective cohorts on 25(OH)D and mortality. There were a number of prerequisites for inclusion of individual cohort studies in ODIN such as availability of bio-banked samples and validated prospective data on clinical outcomes. We subsequently invited representatives of the InCHIANTI (n=1453) study and the Supplementation en Vitamines et Mineraux Antioxydants (SU.VI.MAX; n=13017) study to express interest but they did not respond (SU.VI.MAX) or declined (InCHIANTI). The Danish Osteoporosis Prevention Study (DOPS; n=1006) failed the criteria due to samples inadequate for standardization measurement.

Included studies were the 4^th^ survey of the Tromsø study^34,35^ (n= 26956), the Ludwigshafen Risk and Cardiovascular Health Study^36^ (LURIC; n= 3316), the Age, Gene/Environment Susceptibility-Reykjavik Study^37^ (AGES; n= 5764), the New Hoorn Study^38^ (NHS; n=2807), the Aarhus Mammography Cohort Study^39^ (n=2555), the German Health Interview and Examination Survey for Adults^40,41^ (DEGS; n=4030) and the first (n=1509) and second (n=919) cohort of the Longitudinal Aging Study Amsterdam^42,43^ (LASA). Within each cohort, we standardized cardiovascular and cancer end points on an individual basis, using definitions based on clinical and research expertise, availability of data, published guidelines and definitions, and the Standardized Data Collection for Cardiovascular Trials Initiative.^16^ Definitions were sought to fit as closely as possible by each participating institution.

This meta-analysis is registered at ClinicalTrials.gov, number NCT02438488 and adheres to recommendations for the rationale, conduct, and reporting of IPD meta-analysis.

# Supplementary Figure A. Study flow diagram.

**Identification**

Participants of potentially relevant studies identified and screened for potential retrieval of biobanked serum samples (n=63332)

Participants of studies excluded (n=15476)

♦  No response of study authorities

(n=13017)

♦  Refusal of study authorities to

participate (n=1453)

♦  No availability of biobanked serum

samples (n=1006)

**Screening**

Participants of relevant studies included in the meta analysis (n=47856)

Participants excluded (n=20940)

♦  Missing 25(OH)D measurement

(n=20723)

♦  Lost to follow-up (n=111)

♦  Missing BMI measurement (n=106)

♦  Missing baseline date (n=3)

**Eligibility**

**Inclusion**

Participants included in the meta analysis (n=26916)

Study flow diagram for the collaborative individual-participant-data meta analysis. Potential collaborating study authorities of a work-package of the European Commission-funded ODIN (‚Food-based solutions for eradication of vitamin D deficiency and health promotion throughout the life cycle‘) project were invited to participate. Prerequisite for inclusion of study cohorts were availability of bio-banked samples and validated prospective data on clinical outcomes. Inclusion criterion for the present analysis on the individual participant-level were available participant data for 25-hydroxyvitamin D [25(OH)D] measurements, data on vital status at follow-up and follow-up time/censoring time, data on age, sex, body mass index (BMI) and season of blood sampling (i.e. sampling date).

**2. Study details and missing values**

All following cohort studies and collaborators are part of the ODIN consortium that aims to address the knowledge gaps on the associations between standardized 25[OH]D concentrations and mortality.^33^

**1.** **Tromsø Study:** *Data collection*: The Tromsø Study, conducted by the University of Tromsø in cooperation with the National Health Screening Service, is a repeated population-based study in the municipality of Tromsø, Norway, situated at 69°N.^34,35^ The baseline visit for the present analysis is the fourth survey, which was performed in 1994-1995, with repeated follow-up surveys conducted at six to seven years intervals. Of the 26956 participants, there were missing values for 25-hydroxyvitamin D (25[OH]D) (n=19796), and/or body mass index (BMI) (n=15), so that the final sample for the present study compromised 7145 individuals.

**2. Ludwigshafen RIsk and Cardiovascular Health (LURIC) Study:** *Data collection*: The LURIC Study is a prospective, hospital-based cohort study among 3316 study participants who were routinely referred to a tertiary care medical centre in south-west Germany, situated at 49°N, between 1997 and 2000.^36^ Inclusion criteria were the availability of a coronary angiogram, German ancestry, and clinical stability with the exception of acute coronary syndromes (ACS). Exclusion criteria were any acute illness other than ACS, any chronic disease where non-cardiac disease predominated and a history of malignancy within the past five years. Participants were continuously followed up with respect to fatal events. As there were missing values for 25(OH)D (n=17), the final sample compromised 3299 individuals.

**3.** **Age, Gene/Environment Susceptibility (AGES) Reykjavik Study:**

*Data collection*: The AGES Reykjavik Study is conducted by a collaboration of the National Institute on Aging, National Insitutes of Health, USA and, the Icelandic Heart Association. The AGES-Reykjavik sample is drawn from an established population-based cohort, the Reykjavik Study, and presents a repeated population-based study conducted in Iceland, situated at latitude of about 64°N. AGES-Reykjavik examinations began in 2002. At that time, there were 11549 previously examined Reykjavik Study cohort members still alive. At the end of AGES-Reykjavik examinations in February 2006, the study compromised 5764 survivors of the Reykjavik Study cohort. The AGES-Reykjavik examination is a single wave of examination, completed in three clinic visits, with a participant’s full examination completed within a four to six week time window.^37^ Of the 5 764 individuals, there were missing values for 25(OH)D (n=245), BMI (n=8), and/or mortality follow-up (n=1), so that the final sample compromised 5510 individuals.

**4.** **The New Hoorn Study:**

*Data collection*: From July 2006 until November 2007, the population-based New Hoorn Study (NHS) on glucose tolerance was performed in the city of Hoorn, the Netherlands. A random sample of 6180 men and women aged 40-65 years was drawn from the municipal population registry of Hoorn, situated at 53°N.^38^ Of the 6180 people who were invited, 2 807 agreed to participate (45. 4%). Of the non-attendees, 47% provided a reason for not participating, of which the most common were no time to participate (43%) and already having regular health checks (24. 5%). For the present analysis, 216 individuals of 2807 were excluded, as there were missing frozen samples for measurement of 25(OH)D (n=182), and missing data on mortality follow-up (n=30) and/or BMI (n=4), so that the final sample compromised 2591 individuals.

**5.** **Aarhus Mammography Cohort Study:**

*Data collection*: Between May 1^st^, 2003 and July 1^st^, 2007, 2555 women referred to a diagnostic mammography examination at Aarhus University Hospital (Aarhus, Denmark) were included in the Aarhus Mammography Cohort Study. The primary focus of this study was the association between pre-diagnostic plasma 25(OH)D concentrations and risk of breast cancer. Included women have been followed prospectively with assessement of mortality and incident diesases using The Danish National Hospital Discharge Register and the Danish Cancer Register.^39^ There were missing values for BMI (n=63) and/or mortality follow-up (n=19), so that the final sample compromised 2473 individuals. The mainland of Denmark compromises latitude from 54°N to 57°N.

**6.** **German Health Interview and Examination Survey for Adults (DEGS):**

*Data collection*: DEGS is primarily designed as a periodically repeated cross-sectional national health interview and examination survey of adults in Germany. The target population comprises adults 18-79 years of age with permanent residence in Germany according to local population registries. In order to lay the ground for longitudinal studies, persons who had participated in the 1997-1999 national health interview and examination survey (GNHIES98) were invited to take part in DEGS1 in 2008-2011, provided they had agreed to be re-contacted and were still contactable. Of the 7124 persons who participated in GNHIES98, a subsample of 4030 randomly selected men and women also participated in the affiliated German Nutrition Survey (GeNuS) and had data on serum 25(OH)D concentrations. For the current analysis this random subsample of 4030 participants was selected, and GNHIES98 and GeNuS were set as baseline visit and DEGS1 as follow-up visit. For the current analysis, 168 individuals were excluded due to missing values for age, sex, 25(OH)D (n=113), month of blood sampling and/or BMI (n=16), and/or mortality follow-up (n=39), so that the final sample compromised 3862 individuals.^40,41^ The mainland of Germany compromises latitude from 47°N to 54°N.

**7.** **Longitudinal Aging Study Amsterdam (LASA) - First cohort:**

*Data collection*: LASA is an ongoing multidisciplinary cohort study on predictors and consequences of changes in older persons. In 1992/1993, a random sample of men and women aged 55 years old and over, stratified by age, sex, urbanization grade, and expected five-year mortality rate, was drawn from the population registers of eleven municipalities, in three regions of the Netherlands (n=3107). Measurement cycles were repeated every three years and included a main interview and medical interview. Blood samples were obtained in the first follow-up cycle 1995/1996, which for the present analysis, was defined as baseline visit. The blood samples were centrifuged and stored at −20 °C. Of the 1509 individuals that completed the baseline examination in 1995/96, there were missing values for 25(OH)D (n=189), month of blood sampling (n=3), and/or mortality follow-up (n=18), so that the final sample compromised 1302 individuals.^42,43^ The study compromises latitude from 51°N to 53°N.

**8.** **Longitudinal Aging Study Amsterdam (LASA) - Second cohort:**

*Data collection*: An additional cohort was recruited from the same sampling frame in 2002/2003, exactly ten years after the first LASA cycle of the original cohort. This new cohort consisted of 1002 men and women who were born between 1938 and 1947. Of the 919 individuals with complete demographic data of the baseline examination, there were missing values for 25(OH)D (n=181), and/or missing data on mortality follow-up (n=4), so that the final sample compromised 734 individuals.

**3. Construction of an individual participant database**

Investigators from each study cohort provided individual participant data on a MS Excel template (Microsoft Excel. Redmond, Washington, USA) or translated comparable data into a SPSS (IBM Corp. Released 2011. IBM SPSS Statistics for Windows, NY) or SAS (SAS Institute Inc., 100 SAS Campus Drive, Cary, USA) data file. The template was harmonized prior to the exchange by all investigators to alleviate co-operation and to ensure clear assignment of the data. Data was transmitted to the analysis coordination centre (Medical University of Graz, Austria) by email or by password protected file transfer exchange server. Datasets contained no personally identifiable information, e.g. names or absolute dates. For transmitting, missing data was indicated as “9999” for particular missing values and “8888” for general missing covariates in the datasets. Database management was processed by SPSS version 20 and higher.

**4. Endpoint definitions**

The purpose of this chapter is to provide a framework of definitions for cardiovascular and cancer end points within the current analysis. These definitions are based on clinical and research expertise, availability of data, published guidelines, and definitions, and refer to the Standardized Data Collection for Cardiovascular Trials Initiative.^16^ Definitions were sought to fit as closely as possible by each participating institution.

Cardiovascular death was pre-specified as acute myocardial infarction (ICD-9: 410; ICD-10: I21, I22)^44^, sudden cardiac death (ICD-9: 427; ICD-10: I46-I49, R00)^45^, death due to heart failure (ICD-9: 428; ICD-10: I50)^46^, death due to stroke (ICD-9: 362.3, 430, 431, 433.01, 433.11, 433.21, 433.31, 433.81, 433.91, 434.01, 434.11, 434.91, 436.x; ICD-10: H34.1, I60, I61, I63, I64)^47^, and other causes of cardiovascular death. Other causes of death refer to a cardiovascular death not included in the above categories [e.g. pulmonary embolism, cardiovascular intervention (other than one related to an AMI), aortic aneurysm rupture, or peripheral arterial disease].^16^

Cancer death was defined as death from cancer sites as follows^48^: Lung, trachea, and bronchus (ICD-7: 162; ICD-9: 162; ICD-10: C33, C34), colorectal cancer death (ICD-7: 153, 154; ICD-9: 153, 154; ICD-10: C18-C21), other cancer of the digestive system, i.e., oesophagus, stomach, liver, pancreatic cancer (ICD-7: 150, 151, 155, 157; ICD-9: 150, 151, 155, 157; ICD-10: C15, C16, C22, C25), breast cancer death (ICD-7: 170; ICD-9: 174, 175, 233; ICD-10: C50), prostate cancer death (ICD-7: 177; ICD-9: 185; ICD-10: C61), skin cancer death (ICD-7: 190, 191; ICD-9: 172, 173, 232; ICD-10: C43-C44), and other cancers, including buccal, larynx, melanoma, gynaecological sites, kidney, bladder, brain, multiple myeloma (ICD-7: 140-148, 152, 155.1, 160, 161, 162.2, 172-176, 178-181, 191-197 ;ICD-9: 140-149, 152, 156, 160, 161, 163-165, 170-171, 179, 181-184, 186-189, 190-199, 203, 209; ICD-10: C00 - C14, C17, C23 - C24, C26 - C32, C37 - C41, C45 - C49, C51 - C52, C54 - C60, C62 - C65, C67 - C80, C88, C90, C97).

**1.** **Tromsø Study:** Information on endpoints of all-cause and cause-specific mortality in the Tromsø Study was derived from a National registry. Each participant was linked to data from the Norwegian Causes of Death Registry using a personal identification number to identify vital status. Cause of death is in Norway based on the International Classification of Diseases (ICD) system and the underlying cause of death was used. ICD-9 was applied for deaths occurring up to 1996 and ICD-10 was applied for deaths occurring in 1996 and thereafter.^49^

Cardiovascular death was specified as acute myocardial infarction (ICD-9: 410; ICD-10: I21.9, I22.9), sudden cardiac death (ICD-9: 427.3; ICD-10: I44.1, I45.6, I46.1, I46.9, I49.1, I49.9, I51.4, I51.6, I51.9), death due to heart failure (ICD-9: 428.9; ICD-10: I50.0, I50.1, I50.9), death due to stroke (ICD-9: 430, 431, 433.1, 434.9, 436; ICD-10: H34.0, I60.0, I60.1, I60.2, I60.8, I60.9, I61.2, I61.3, I61.4, I61.9, I62.0, I63.0, I63.1, I63.2, I63.3, I63.4, I63.9, I64, I69.1, I69.3, I69.4), and other causes of cardiovascular death.

Other causes of cardiovascular death contained coronary/peripheral atherosclerosis (ICD-9: 414.0, 414.3, 414.8, 414.9; ICD-10: I70.2, I70.9), unspecified cerebrovascular/peripheral vascular disease (ICD-9: 437.9; ICD-10: I73.9, I67.2, I67.8, I67.9), generalized and unspecified atherosclerosis (ICD-9: 440.9), aneurysm (ICD-9: 441.0, 441.1, 441.4, 441.5, 441.6; ICD-10: I71.0-I71.5, I71.8, I71.9), endocarditis (ICD-10: I38, I33.0), atrial fibrillation, (rheumatic) valve disease (ICD-9: 394.9, 424.1; ICD-10: I05.8, I06.0, I06.9, I08.0, I35.0, I35.1, I35.2, I35.9), hypertensive (heart) disease (ICD-10: I10, I11.0), hypertensive kidney disease (ICD-10: I12.0, I12.9, I13.2), angina pectoris (ICD-10: I20.9), and ischaemic heart disease (ICD-10: I24.1, I25.1, I25.2, I25.5), thrombosis and embolism (ICD-10: I26.9, I74.2, I74.3, I80.2, I80.3), other pulmonary heart diseases (ICD-10: I27.0, I27.9), chronic constrictive pericarditis (ICD-10: I31.1), cardiomyopathy (ICD-10: I42.0, I42.2, I42.6, I42.9), myocarditis (ICD-10: I45.6), arteritis (ICD-10: I77.6).

Cancer death was defined as death from cancer sites as follows: Lung, trachea, and bronchus (ICD-9: 162.9; ICD-10: C33, C34.0-C34.3, C34.9), colorectal cancer death ( ICD-9: 153; ICD-10: C19, C20, C18.0-C18.7, C18.9, C21.0, C21.1), other cancer of the digestive system, i.e., oesophagus, stomach, liver, pancreatic cancer (ICD-9: 150, 151, 155.0, 155.1, 157; ICD-10: C15.4, C15.5, C15.8, C15.9, C16.0, C16.2-C16.6, C16.8, C16.9, C22.0, C22.1, C23, C25.0, C25.1, C25.8, C25.9), breast cancer death (ICD-10: C50.2- C50.5, C50.8, C50.9), prostate cancer death (ICD-9: 185; ICD-10: C61), skin cancer death (ICD-9: 172; ICD-10: C43.3, C43.5-C43.7, C43.9), and other cancers, including buccal, larynx, melanoma, gynaecological sites, kidney, bladder, brain, multiple myeloma (ICD-9: 161, 171, 183.0, 184.9, 188, 190, 199.1, 200.1, 202.1, 204.1; ICD-10: C01, C02.1, C02.9, C04.9, C07, C08, C09.9, C13.9, C14, C17.9, C24.0, C24.1, C24.9, C26.9, C31, C32.0, C32.2, C32.9, C38.0, C38.4, C41.0, C45.0, C45.7, C45.9, C48.0-C48.2, C49.0, C49.2, C49.9, C51.9, C52, C53.9, C55, C56, C54.9, C57.4, C57.9, C64, C65, C73, C67.9, C68.9, C69.3, C71.0, C71.2, C71.9, C74, C75.9, C76.2, C80, C81.9, C82.1, C83.0, C83.1, C83.3, C83.7, C85.1, C85.7, C85.9, C88.0, C90.0, C91.0, C91.1, C91.3, C91.9, C92.0, C92.1, C92.5, C95.9, C96.1).

**2. Ludwigshafen RIsk and Cardiovascular Health (LURIC) Study:**

Information about vital status was obtained from local person registries. Medical records of local hospitals, death certificates, and autopsy data were used to classify the causes of death into cardiovascular and non-cardiovascular mortality. Classification of the causes of death was independently done by two experienced physicians who were blinded to any data of the study subjects except of those that were necessary for the coding of the causes of death. In the event of a disagreement regarding a specific case, the final classification was done by one of the principle investigators of LURIC (W. M.).^50^

**3.** **Age, Gene/Environment Susceptibility (AGES) Reykjavik Study:**

Information on vital status and the causes of death was based on data from a complete adjudicated registry of deaths available from the Icelandic National Roster.^51^

Cardiovascular death was pre-specified as acute myocardial infarction (ICD-10: I21, I22), sudden cardiac death (ICD-10: I47-I49, R00), death due to heart failure (ICD-10: I50), death due to stroke (ICD-10: H34.1, I60, I61, I63, I64,G45). Other causes refer to a cardiovascular death not included in the above categories [e.g. pulmonary embolism, cardiovascular intervention (other than one related to an AMI), aortic aneurysm rupture, or peripheral arterial disease].

Cancer death was defined as death from cancer sites as follows: Lung, and bronchus (ICD-10: C34), colorectal cancer death (ICD-10: C18-C21), other cancer of the digestive system, i.e., oesophagus, stomach, pancreatic cancer (ICD-10: C15, C16, C25), breast cancer death (ICD-10: C50), prostate cancer death (ICD-10: C61), skin cancer death (ICD-10: C43-C44), and other cancers, including buccal, larynx, melanoma, gynaecological sites, kidney, bladder, brain, multiple myeloma (ICD-10: C00-D49, other than listed above).

**4.** **The New Hoorn Study:**

Information on vital status was obtained from the Dutch Municipal Population Register. No specific causes of death have been yet coded.

**5.** **Aarhus Mammography Cohort Study:**

Information on endpoints of all-cause and cause-specific mortality was derived from The Danish National Hospital Discharge Register and the Danish Cancer Register.^52^

Data from death certificates were used to decide the fundamental cause of death. Coding was based on the ICD-10 classification, but the coding has been performed by the physician filling in the death certificate. ICD-10 codes I00-I99 have resulted in a death being considered as a cardiovascular death, and C00-D49 have resulted in a death being classified as due to cancer.

**6.** **German Health Interview and Examination Survey for Adults (DEGS):**

Information on endpoints of all-cause and cause-specific mortality in the German Health Interview and Examination Survey for Adults was derived from local population registries and death certificates.^53^ Cardiovascular death was specified in ICD-10 codes as I00 to I99. Cancer death was specified in ICD 10 codes as C00 toC97.

**7.** **Longitudinal Aging Study Amsterdam, first and second cohort**:

Occurrence of deaths among the study participants was retrieved up to 1^st^ November 2013 through linkage with population register data. Primary causes of death were obtained from the Dutch Central Bureau of Statistics. The cause of death was coded according to the International Classification of Diseases, 10th Revision (ICD-10).^54^

Cardiovascular death was pre-specified as acute myocardial infarction (ICD-9: 410; ICD-10: I21, I22), sudden cardiac death (ICD-9: 427; ICD-10: I46-I49, R00), death due to heart failure (ICD-9: 428; ICD-10: I50), death due to stroke (ICD-9: 362.3, 430, 431, 433.01, 433.11, 433.21, 433.31, 433.81, 433.91, 434.01, 434.11, 434.91, 436.x; ICD-10: H34.1, I60, I61, I63, I64), and other causes of cardiovascular death. Other causes included pulmonary embolism, aortic aneurysm rupture, or peripheral arterial disease (ICD-9: 415.1, 441.3, 441.6, 440.2; ICD-10: I26.9, I71.0, I71.3, I71.4, I71.9, I73.9).

Cancer death was defined as death from cancer sites as follows: Lung, trachea, and bronchus (ICD-9: 162; ICD-10: C33, C34), colorectal cancer death (ICD-9: 153, 154; ICD-10: C18-C21), other cancer of the digestive system, i.e., oesophagus, stomach, liver, pancreatic cancer (ICD-9: 150, 151, 155, 157; ICD-10: C15, C16, C22, C25), breast cancer death (ICD-9: 174, 175, 233; ICD-10: C50), prostate cancer death (ICD-9: 185; ICD-10: C61), skin cancer death (ICD-9: 172, 173, 232; ICD-10: C43-C44), and other cancers, including gynaecological sites, and malignant neoplasms of lymphoid, hematopoietic and related tissue (ICD-10: C53.9, C81.9, C85.9, C91.0, C91.1, C92.0, C92.1).

**5. Covariate definitions**

Age was defined as age (years) at the baseline visit and inserted as a linear term in the regression models. Sex was inserted as a binary variable coding as “0” for women and “1” for men. Body mass index (BMI) was defined as weight in kilograms divided by height in meters squared and inserted as linear term. Season of blood sampling was coded from information on month of blood sampling. Season of blood sampling at baseline was coded as dummy variable for each of the four Seasons, with spring defined as March to May (with coding “0” for baseline blood sampling not during spring, and “1” for during spring ), summer as June to August (with coding “0” for baseline blood sampling not during summer, and “1” for during summer), autumn as September to November (with coding “0” for baseline blood sampling not during autumn, and “1” for during autumn), and winter as December to February (with coding “0” for baseline blood sampling not during winter, and “1” for during winter). Autumn was defined as reference season and left out in the regression equation.^19^ Supplemental intakes of calcium and vitamin D were defined as intake by supplements at baseline. Supplemental intake of calcium was defined as with definitions listed according to priority - if more than one point was available in a study the higher ranked definition was given priority: ATC code A12AA, A12AX, or questionnaire declaration. Supplemental intake of vitamin D was defined as with definitions listed according to priority - if more than one point was available in a study the higher ranked definition was given priority: ATC codes A11CC01, A11CC04, A12AX, or questionnaire declaration. Supplemental calcium intake was coded as a binary variable coding as “0” for no supplemental intake and “1” for positive supplemental intake of calcium at the baseline visit. Supplemental vitamin D intake was coded as a binary variable coding as “0” for no supplemental intake and “1” for positive supplemental intake of vitamin D at the baseline visit. Present arterial hypertension was defined as with definitions listed according to priority - if more than one point was available in a study the higher ranked definition was given priority: Participants already on antihypertensive drug treatment, physician-reported arterial hypertension, self-reported arterial hypertension, office systolic and/or diastolic blood pressure of equal to or higher than 140 and/or 90 mmHg according to 2003 guidelines of the European Society of Hypertension (ESH) and the European Society of Cardiology (ESC).^55^ Present arterial hypertension was inserted as a binary variable with “0” coding for no arterial hypertension present and “1” for arterial hypertension present at baseline. Present diabetes mellitus was specified as with definitions listed according to priority - if more than one point was available in a study the higher ranked definition was given priority: Those participants on glucose lowering drugs, physician-reported, self-reported or according to American Diabetes Association (ADA) criteria fasting glucose ≥ 7.0 mmol/L, two hours post-load glucose ≥ 11.1 mmol/L or HbA1c ≥ 6.5%.^56^ Present diabetes mellitus was inserted as a binary variable with “0” coding for no diabetes mellitus present and “1” for diabetes mellitus present at baseline. Current smoking habit was dichotomised as no active smoking (“0”) versus active smoking (“1”) at baseline. Physical activity was coded in three levels according to the frequency of leisure activity in hours per week from low to high frequency.^57^ Each level was transformed into a dummy variable and inserted into a regression model as a binary variable coding for “0” level not present versus “1” level present and the third (highest frequency) group was defined as reference and left out in the regression equation. Basically, physical activity was defined as leisure activity, with a degree of medium or vigorous intensity (for example bicycling, swimming, tennis, gardening, distance walking). If calculated, medium or vigorous intensity were determined as metabolic equivalent of task (MET) of three and higher, equivalent to 600-1499 kcal per week. The lowest level of physical activity includes participants on moderate and vigorous activity with a frequency of less than one hour per week. Additionally, participants without participating in any regular leisure activity (e.g. bedridden participants) or participating only in light activities (intensity MET-score ≤2.9, e.g. light household, billiards, walking slowly) were included into the lowest level of physical activity, regardless of their frequency. Participants assigned to light activities are defined as participants who are not participating in any moderate or vigorous activities or any regular activity with MET-score of three and higher. The medium level of physical activity compromises participants with moderate and vigorous activity (intensity MET-score of three and higher) with a frequency of one to three hours per week and the highest level of physical activity compromises participants with moderate and vigorous activity (intensity MET-score of three and higher) with a frequency of more than three hours per week. History of cardiovascular disease (CVD) was coded as binary variable “0” for negative and “1” for positive history. History of CVD included previous acute myocardial infarction and previous stroke/ transient ischemic attack at the baseline visit. History of cancer was a binary variable coding as “0” for negative and “1” for positive history of cancer at baseline. History of cancer included cancer of lung, trachea, and bronchus, colorectal cancer, oesophagus-, stomach-, liver-, and pancreatic cancer, breast cancer, prostate cancer, skin cancer, and other cancer sites including buccal, larynx, melanoma, gynaecological sites, kidney, bladder, brain, and multiple myeloma. Estimated glomerular filtration rate in mL/min/1.73m² was calculated from creatinine at baseline visit according to the four-variable Modification of Diet in Renal Disease (MDRD) Study equation and inserted as a linear term in the regression equations. C-reactive protein at baseline was inserted as linear term in mg/L. Parathyroid hormone (PTH) was inserted as linear term in pmol/L, low density lipoprotein (LDL) was inserted as linear term in mmol/L, and systolic blood pressure as linear term in mmHg in the regression equation.

**6. Covariate assessment**

Appendix table 1: List of additional baseline confounders available for model building of parametric survival model in the eight observational cohort studies included in the meta-analysis.

| **Characteristic** | **Tromsø** | **LURIC** | **AGES** | **NHS** | **Aarhus** | **DEGS** | **LASA, first cohort** | **LASA, second cohort** |
| --- | --- | --- | --- | --- | --- | --- | --- | --- |
| Age | X | X | X | X | X | X | X | X |
| Sex | X | X | X | X | X | X | X | X |
| Season | X | X | X | X | X | X | X | X |
| BMI | X | X | X | X | X | X | X | X |
| Current smoking | X | X | X | X | X | X | X | X |
| Present Diabetes | X | X | X | X | X | X | X | X |
| Present HTN | X | X | X | X | X | X | X | X |
| History of CVD | X | X | X | N.A. | X | X | X | X |
| History of cancer | X | X | X | N.A. | X | X | X | X |
| Physical activity | X | X | X | X | N.A. | X | X | X |
| eGFR | X | X | X | N.A. | X | X | X | X |
| CRP | N.A. | X | X | N.A. | N.A. | X | X | N.A. |
| Calcium Supplements | X | X | X | N.A. | X | N.A. | X | N.A. |
| Vitamin D Supplements | X | X | X | N.A. | X | N.A. | N.A. | N.A. |
| PTH | X | X | X | N.A. | X | X | X | X |
| SBP | X | X | X | X | N.A. | X | X | X |
| LDL-C | X | X | X | X | N.A. | X | X | X |
| Glucose | N.A. | X | X | X | N.A. | X | X | X |

Abbreviations: Tromsø = Tromsø Study; LURIC = Ludwigshafen RIsk and Cardiovascular Health Study; AGES = Age, Gene/Environment Susceptibility Reykjavik Study; NHS = New Hoorn Study; Aarhus = Aarhus Mammography Cohort Study; DEGS = German Health Interview and Examination Survey for Adults; LASA = Longitudinal Aging Study Amsterdam; X = Confounder available; N.A. = Confounder not available; BMI = Body mass index; HTN = Arterial hypertension; CVD = Cardiovascular disease; eGFR = estimated glomerular filtration rate according to the four-variable Modification of Diet in Renal Disease (MDRD) Study equation; CRP = C-reactive protein; PTH = Parathyroid hormone; SBP = Systolic blood pressure; LDL-C = Low density lipoprotein cholesterol. Basic adjustment variables age, sex, BMI, season of blood sampling, vital status at follow up and follow-up/failure time were minimum requirements and were available in all cohorts.

**1.** **Tromsø Study:**

*Present diabetes*: The diagnosis of diabetes mellitus was derived from questionnaires (“Do you have, or have you had diabetes?”), through elevated HbA1c (6.5%), by linkage of the participant list to the University Hospital of North Norway digital discharge diagnosis registry (ICD-9 codes 250, 357.2, 362.0, 583.8, 648.0, 648.8, 790.2, ICD-10 codes E10.0-E14, O24 and R73) and hospital records.

*Present arterial hypertension*: Present diagnosis of arterial hypertension was established as intake of antihypertensives or office systolic and/or diastolic blood pressure of equal to or higher than 140 and/or 90 mmHg.

*Physical activity*: Information on physical activity was obtained from a questionnaire on light and hard leisure activity. Light activity was asked for as “*Light activity (not sweating or out of breath): How has your physical activity in leisure time been during this last year? Think of your weekly average for the year. Time spent going to work count as leisure time (hours per week).*” Hard leisure activity was obtained as followed: “*Hard physical activity (sweating/out of breath): How has your physical activity in leisure time been during this last year? Think of your weekly average for the year. Time spent going to work count as leisure time (hours per week).*” For recoding only the Tromsø variable of hard leisure activity was used, and the weekly frequency was divided from a four-levelled scale, which compromised the answers, “no activity”, “less than one hour per week”, “one to two hours per week”, and “three hours or more per week”, into the three-levelled co-variable according to the current definition. For re-coding, the lowest level of hard leisure activity was kept as it as, answer two and three were combined to the medium level of physical activity and answer four was re-coded into the highest level of physical activity.

*Active smoker status*: Active smoking status was obtained from questionnaire (“*Do you smoke cigarettes daily?*”).

*History of cardiovascular disease*: Positive history of cardiovascular disease was defined as positive history of myocardial infarction or positive history of stroke. History of myocardial infarction and stroke were self-reported (“*Do you have, or have you had a heart attack*?”, and “*Do you have, or have you had a cerebral stroke/brain haemorrhage*?”) and obtained from medical records.

*History of cancer*: Information on cancer incidence and cancer location was retrieved from the Cancer Registry.

*Creatinine*: Plasma creatinine in µmol/L was analysed by a modified Jaffe reaction on a Roche Hitachi 911 automated analyser (Boehringer Mannheim/Hitachi, Indianapolis, USA).

*Intake of calcium by supplements*: Supplemental intake of calcium was obtained from questionnaires (“*Have you used calcium tablets or bone meal during the last 14 days?*”) and hospital records.

*Intake of vitamin D by supplements*: Supplemental intake of vitamin D was obtained from questionnaires (“*Have you used vitamin D supplement during the last 14 days?*”) and hospital records.

*Measurement of serum 25(OH)D*: Sera from the second visit of the study were stored at −70 °C, and after a median storage time of 13 years, they were thawed in March 2008 and analysed for 25(OH)D3 by electrochemiluminescence immunoassay (ECLIA) from Roche (Roche Diagnostics, Mannheim, Germany) using an automated clinical chemistry analyser (Modular E170, Roche Diagnostics, Mannheim, Germany). According to the manufacturer, the assay has, for total analytical precision, a coefficient of variation ≤7.8% as judged in any of three different concentrations (48.6, 73.8, and 177.0 nmol/L). The cross-reactivity with 25(OH)D2 was <10%, and the analytical sensitivity was 10 nmol/L.^34^ Since this assay overestimated 25(OH)D in smokers^58^, standardization was performed separately for smokers and non-smokers.

*Body mass index*: Height and weight were measured while the subjects wore light clothing and no shoes and body mass index (BMI) was calculated as kg/m².^35^

*Blood pressure*: Blood pressure was measured with an automatic device (Dinamap Vital Signs Monitor 1846; Critikon Inc, Tampa, FL). The subjects were seated for two minutes. Three recordings were made at two minutes intervals, and the mean of the last two measurements was used.

*Low-density lipoprotein*: In the fourth survey of the Tromsø Study, serum total cholesterol (TC) and triacylglycerol (TAG) concentrations were analysed by enzymatic colorimetric methods with commercial kits (CHOD-PAP for TC and GPO-PAP for TAG; Boehringer-Mannheim, Mannheim, Germany). Serum high-density lipoprotein cholesterol (HDL-C) was measured after the precipitation of lower-density lipoproteins with heparin and manganese chloride. The serum low-density lipoprotein cholesterol (LDL-C) was calculated using the formula LDL-C=TC−HDL-C−(TAG × 0.46), provided the serum TAG value was <4.0mmol/L.^59^

*Parathyroid hormone*: Intact PTH was measured on Immulite (Diagnostic Products Corporation, Los Angeles, CA, USA) based on a two-site chemiluminescent immunometric assay. The reference range in our laboratory is 1.1-6.8 pmol/l for those below the age of 50, and 1.1-7.5 pmol/l for those 50 years or above, the between assay coefficient of variation (CV) being 6-8% in the actual range.^60^

**2. Ludwigshafen RIsk and Cardiovascular Health (LURIC) Study:**

*Present diabetes*: Diabetes mellitus was diagnosed according to the criteria published by the American Diabetes Association (ADA) 1997^56^ and by the World Health Organization (WHO)^61^. These criteria for the definition of diabetes mellitus either demanded a pathological fasting (plasma glucose ≥ 126 mg/dL) or a two hour plasma glucose concentration ≥ 200 mg/dL after an oral glucose tolerance test. In addition, an individual with a documented history of diabetes was defined as diabetic, regardless whether the laboratory criteria for diabetes were met or not. Requirement for an antidiabetic medication, i.e., oral antidiabetic and/or insulin use for the control of glycaemia, was accepted as a documentation of diabetes, as were medical documents (i.e., hospital discharge letters or other documentation) with a diagnosis of diabetes (including diabetics treated by diet only).

*Present arterial hypertension*: Present diagnosis of arterial hypertension was established as intake of antihypertensives or office systolic and/or diastolic blood pressure of equal to or higher than 140 and/or 90 mmHg.

*Physical activity*: Daily physical activity was recorded using a non-validated 11-point scale ranging from bedridden to extremely active. Key points on the scale were “1” meant bed rest;” 2” meant mostly supine, “3” meant not very active, “6” meant usual office work, “9” meant heavy work or sports, and “11” meant extremely sportive. Recoding was performed according to WHO reccomendations into (1 thru 7=1), (8=2) and (9 thru 11=3).^62^

*Active smoker status*: Active smoking status was self-reported (“*Do you smoke cigarettes regularly?*”).

*History of cardiovascular disease*: Positive history of cardiovascular disease was defined as positive history of myocardial infarction or positive history of stroke. Previous event of stroke was obtained from medical records. A previous myocardial infarction (MI) was diagnosed if a MI had been survived for more than one month before enrolment into LURIC. The diagnosis could either be based on ECG (see Winkelmann BR et al.^36^ for detailed information), or it was based on a report of a diagnosis of MI in a medical document (discharge letter, catheterisation report). Additionally, previously unrecognised MI (often called silent MI) was included as history of CVD. Silent MI was defined as a subtype of previous MI, where the individual had not been hospitalised during the acute phase of the MI and the diagnosis of MI was established in retrospect. Previously unrecognised MI was diagnosed in the presence of new pathologic Q-waves in at least two adjacent leads with or without wall motion abnormalities. Isolated documentation of wall motion abnormality, vessel occlusion without further clinical evidence or evidence of scarring by imaging was not sufficient for the diagnosis of a previously unrecognised MI.

*History of cancer*: Participants who had malignant cancer in the previous five years were excluded from the LURIC Study. Information on history of cancer prior to the previous five years was obtained from questionnaire.

*Creatinine*: Serum creatinine was measured by Jaffé method (twin mode) on a CREA/Hitachi 717 automated analyser (Roche Mannheim, Germany).

*C-reactive protein*: C-reactive protein was analyzed by an immunoturbidimetric assay on a Hitachi 911 analyzer (Roche Mannheim, Germany)

*Intake of calcium by supplements*: Supplemental intake of calcium was obtained from medication history questionnaire with explicit field for vitamin intake. Retrospective search tags were Ossofortin, Ortho core PLUS, Ca BT, Calcium BT, Phosetamin, Calcium Dura, Solugastril, Calcium Sandoz, Calcium forte, Calcium, Calcimagon, Frubase Ca forte, Ca-Acetat , also multivitamin preparations were taken into account.

*Intake of vitamin D by supplements*: Supplemental intake of vitamin D was obtained from medication history questionnaire with explicit field for vitamin intake. Retrospective search tags were Vigatoletten, Ossofortin, Rocaltrol, Vitamin D3, D-tracetten, Frubase Ca forte, and Calcimagon. Also, multivitamin preparations were taken into account.

*Body mass index*: Body weight was measured without shoes and in light clothing by a trained nurse. Body height was recorded to the nearest centimetre with the subject barefoot and in the upright position.

*Blood pressure*: Blood pressure was measured with an automated oscillometric device (Omron MX4, Omron Healthcare GmbH, Hamburg, Germany) while supine for at least 10 minutes. At least three consecutive measurements of systolic and diastolic blood pressures were taken 30 seconds apart and mean values of these measurements are reported. Measurements were considered invalid and repeated if they varied > 10 mmHg systolic, > 5 mmHg diastolic, or heart rate of more than five beats per minute from each other (except for atrial fibrillation).^36^

*Low-density lipoprotein cholesterol*: Low-density lipoprotein cholesterol was measured after separating lipoproteins with a combined ultracentrifugation-precipitation method.

*Parathyroid hormone*: Intact PTH was determined in serum by ElectroChemiLuminescence Immunoassay (ECLIA) on an Elecsys 2010 (Roche Diagnostics, Mannheim, Germany), with a normal range of 15-65 pg/mL and an inter-assay coefficient of variation of 5.7-6.3%.

*Parathyroid hormone*: Intact PTH was determined in serum by ElectroChemiLuminescence Immunoassay (ECLIA) on an Elecsys 2010 (Roche Diagnostics, Mannheim, Germany), with a normal range of 15-65 pg/mL and an inter-assay coefficient of variation of 5.7-6.3%.

**3.** **Age, Gene/Environment Susceptibility (AGES) Reykjavik Study:**

*Present diabetes*: Diabetes was defined using glucose lowering drugs, or fasting glucose ≥ 7.0 mmol/L and self-report according to questionnaire (“*Has a doctor or other health personnel ever told you that you had diabetes?*”).

*Present arterial hypertension*: Information on present arterial hypertension was obtained either from questionnaire (“*Has a doctor or other health provider ever told you that you had hypertension or high blood pressure?*”), from physiological measurements (office systolic and/or diastolic blood pressure of equal to or higher than 140 and/or 90 mm Hg), or usage of antihypertensive medication (specifically Anatomical Therapeutic Chemical (ATC) classification code beginning with C02).

*Physical activity*: Participants answered questions about frequency of current moderate or vigorous physical activity (“*How often did you participate in moderate or vigorous physical activities in the past 12 months*?”). Answers were categorised into never, rarely, occasionally, moderate or high frequency of participation. Options one to three were combined to basic level of physical activity, option four was assigned the second level of physical activity and options five and six were set level three. Participants with only light activities were assigned to basic level of physical activity.

*Active smoker status*: Active smoking status was self-reported (“*Do you smoke cigarettes now?*” and “*Do you smoke cigar or pipe now?*”).

*History of cardiovascular disease*: History of cardiovascular disease was defined as history of myocardial infarction or history of stroke. History of myocardial infarction and stroke were either self-reported obtained from the Directorate of Health or from hospital records.

*History of cancer*: History of cancer was obtained from hospital records.

*Creatinine*: Serum creatinine was measured using the Roche-Hitachi 912 instrument with Roche Creatinine Jaffé compensated method; Roche Diagnostics, Mannheim, Germany. The coefficient of variation for the creatinine assay was 2.5%.^63^

*C-reactive protein*: High sensitivity CRP was measured on a Hitachi 912, using reagents from Roche Diagnostics and following the manufacturer’s instructions. Both within- and between-assay quality control procedures were used and the coefficient of variation of the method was 1.3% to 3.4%, respectively, through the period of data collection. The assay could detect a minimal CRP concentration of 0.1 mg/L and values below this level were classified as undetectable. All participants in this study had detectable CRP levels.^64^

*Intake of calcium by supplements*: Calcium intake by supplements was defined as use of any calcium supplements according to questionnaire or list of „Over the counter drug use“. Question asked was „Do you take calcium tablets?“.

*Intake of vitamin D by supplements*: Intake of vitamin D supplements was defined as regular use of any vitamin D supplements according to questionnaire or list of „Over the counter drug use“. In the definition all occurrences of products with information on vitamin D supplements, multivitamins, and fish liver oil were used. People were also asked to bring all medications and supplements to the AGES-visit. Questions asked were „Do you take multi-vitamins?“ and „On average how often do you take cod or Saithe liver oil or liver oil pills (not halibut liver oil pills)?“

*Measurement of serum 25(OH)D*: Blood was collected during the first clinic visit to AGES-study, from September 2002 to January 2006, and fasting serum samples were kept frozen at −80°C on-site in the IHA biorepository. Quantitative determination of total 25(OH) D (D2 and D3) was conducted by means of a direct, competitive chemiluminescence immunoassay (CLIA), using the LIAISON 25 OH Vitamin D Total assay (DiaSorin, Inc., Stillwater, Minnesota). The inter-assay coefficient of variation was <6.5%, using a previously frozen serum pool as the control sample and <12.7% when the calculated data were from measurements using Liaison quality controls.^65^

*Body mass index*: Body mass index was calculated as weight in kilogram divided by height in meters squared. Baseline weight was measured using a digital scale and height was measured using a stadiometer.

*Blood pressure*: *Blood pressure*: Blood pressure was measured with a mercury sphygmomanometer with a large cuff, and the mean value of two consecutive blood pressure measurements was used in the analysis.

*Low-density lipoprotein*: Total cholesterol (TC), high-density lipoprotein (HDL) cholesterol, and triglycerides were analysed on a Hitachi 912 chemistry automated analyser, using reagents from Roche Diagnostics and following the manufacturer's instructions (Roche Diagnostics GmbH, Mannheim, Germany). Low-density lipoprotein (LDL) was calculated using the Friedewald equation.

*Parathyroid hormone*: Intact PTH was measured on Roche Elecsys 2010 analyser from Roche Diagnostics GmbH, Mannheim using electrochemiluminescence technology on a two-site immunoassay. PTH values were converted from pg/mL to pmol/L by multiplication with 0.106. The inter-assay coefficient of variation was less than 3.1% when using a frozen serum pool as the control sample and was less than 2.8% when using Roche quality controls.^65^

*Glucose*: Glucose was analysed on a Hitachi 912 chemistry automated analyser, using reagents from Roche Diagnostics and following the manufacturer's instructions (Roche Diagnostics GmbH, Mannheim, Germany). Inter-assay coefficient of variation was 2.0%.

**4.** **The New Hoorn Study:**

*Present diabetes*: Based on the results of the OGTT, participants were categorized into 3 groups using the WHO ’06 criteria (1): normal glucose metabolism (NGM), impaired glucose regulation, i.e. impaired fasting glucose (IFG) and/or impaired glucose tolerance (IGT) based on fasting glucose greater of equal 6.1 mmol/l and/or 2h post OGTT glucose greater of equal 7.8 mmol/l or newly detected diabetes mellitus (NDM). In addition, known diabetes mellitus (KDM) was defined by the use of insulin or oral hypoglycaemic agents, and self-reported known diabetes (“*Have you ever been told by a medical doctor (or another health professional) that you have diabetes?*”). For the present analysis participants with known, as well as newly diagnosed and participants with IFG were considered positive for this variable.^61^

*Present arterial hypertension*: Present arterial hypertension was defined in participants already on antihypertensive drug treatment or according to WHO 2003 statement^66^ and in participants with office systolic and/or diastolic blood pressure of equal to or higher than 140 and/or 90 mm Hg.

*Physical activity*: Physical activity was obtained from questionnaires on leisure activity, with focus on frequency and degree of exertion. Information on four kinds of regularly performed sports plus biking was obtained. After recoding each type of activity from minutes per day into hours per week, the overall time for no activity, light and moderate to vigorous degree of exertion was calculated for every type of sports and biking. Then, the overall weekly time for each level of intensity was calculated as the sum of different kinds of sports performed at each level of intensity. The time variables were then stratified into three levels of frequency separately for light and moderate to vigorous intensity. For moderate to vigorous intensity, frequency of less than one hour per week was re-coded to the lowest level of physical activity, one to three hours per week were set as the medium level of physical activity and three hours or more per week were set as the highest level of physical activity. For light intensity, frequency of less than two hour per week was re-coded to the lowest level of physical activity, two to six hours per week were set as the medium level of physical activity and six hours or more per week were set as the highest level of physical activity. Finally, out of the stratified frequency variables of light and moderate to vigorous intensity, the participant was signed the higher value out of both variables.

*Active smoker status*: Information on active smoking status was obtained from questionnaire (“*Do you smoke?*”).

*Measurement of serum 25(OH)D*: The NHS was the only cohort study with no original 25(OH)D measurements; so that the NHS was not calibrated, but measured in full a liquid chromatography-mass spectrometry method at University College Cork.

*Body mass index*: Height and weight were measured without shoes and heavy clothes. Body mass index was calculated as weight (in kg) divided by height (in meters) square.

*Blood pressure*: Blood pressure was measured three times on the right arm after a 10 minute rest period, using a Colin Press BP 8800p Non-Invasive Blood Pressure Monitor (Colin Medical Technology Corporation, USA). Final blood pressure was calculated as the mean of the last two measurements.

*Low-density lipoprotein*: Triglycerides, total and high density lipoprotein cholesterol were determined from fasting plasma samples by enzymatic techniques (Boehringer-Mannheim, Mannheim, Germany). Low-density lipoprotein cholesterol was estimated with the Friedewald formula, except in individuals with triglycerides >4.5 mmol/l.

*Glucose*: Glucose was measured in venous plasma by the glucose-oxidase method (Glucoquant/hexokinase/G6P-DH; Boehringer-Mannheim, Mannheim, Germany). HbA1c was assessed using a DCCT standardized reversed-phase cation exchange chromatography (HA8160 analyser, Menarini, Florence, Italy). The intra-assay coefficient of variation (CV) was 0.65% at a mean of 4.89% and the inter-assay CV 1.55% at a mean of 5.52%.

**5.** **Aarhus Mammography Cohort Study:**

*Present diabetes*: The diagnosis is based on a hospitalization discharge (ICD) code, self-reported treatment with anti-diabetic drugs, or both.

*Present arterial hypertension*: The diagnosis is based on a hospitalization discharge (ICD) code, self-reported treatment with anti-hypertensive drugs, or both.

*Active smoker status*: Smoking status was self-reported by the participants in a self-administered questionnaire (“*Do you smoke*?)”.

*History of cardiovascular disease*: Positive history of cardiovascular disease was defined as positive history of myocardial infarction or positive history of stroke. History of stroke and history of myocardial infraction were retrieved from The Danish National Hospital Discharge Register.

*History of cancer*: History of cancer was retrieved from The Danish National Hospital Discharge Register and the Danish Cancer Register.

*Creatinine*: Creatinine was measured by standard laboratory methods at the Aarhus University Hospital laboratory.

*Intake of calcium by supplements*: The use of calcium supplements was self-reported in a self-administered questionnaire (“*Do you daily use supplements of calcium? If yes, please state the name of the supplement*”).

*Intake of vitamin D by supplements*: The use of vitamin D supplements, including use of multivitamin pills, was self-reported in a self-administered questionnaire (“*Do you daily use supplements of vitamin D? If yes, please state the name of the supplement*” and “*Do you use multivitamin tablets?*”).

*Measurement of serum 25(OH)D*: Prior to the mammography, a blood sample from each participant was collected. Samples were divided in aliquots and stored immediately at −80°C until analysis. At the end of the study, plasma 25(OH)D concentrations were analysed by isotope dilution liquid chromatography-tandem mass spectrometry by a method adapted from Maunsell et al.^67^. Mean coefficients of variation for 25(OH)D3 were 6.4% and 9.1% at concentrations of 66.5 and 21.1 nmol/L.^39^

*Body mass index*: Height and weight were self-reported by the participants in a self-administered questionnaire, which was filled in prior to the mammography examination.

*Parathyroid hormone*: Intact parathyroid hormone (PTH) was measured in plasma using a second-generation electrochemiluminescent immunoassay (ECLIA) on an automated instrument (Cobas e601; Roche Diagnostics, GmbH, Mannheim, Germany). According to the manufacturer, the reference interval for PTH concentrations is 1.6-6.9 pmol/l. The lower limit of detection was 0.127 pmol/l, and total imprecision (CV%) was 3.3% at 3.69 pmol/l and 2.7% at 26.6 pmol/l.

**6.** **German Health Interview and Examination Survey for Adults (DEGS):**

*Present diabetes*: Diabetes mellitus was defined as either a self-reported history of physician-diagnosed diabetes in a standardized physician-administered computer-assisted personal interview (CAPI), or current intake of glucose lowering drugs (Anatomical Therapeutic Chemical [ATC] classification code A10) within the last seven days prior to baseline examination, or a baseline hbA1c value of ≥6.5% according to ADA 2010 criteria.^56,68^ Participants were asked “*Have you ever been diagnosed with diabetes by a doctor*?”.

*Present arterial hypertension*: Present diagnosis of arterial hypertension was defined as either a self-reported history of physician-diagnosed hypertension or office systolic and/or diastolic blood pressure of equal to or higher than 140 and/or 90 mmHg. Participants were asked “*Have you ever been diagnosed with high or elevated blood pressure by a doctor*?”.

*Physical activity*: Participants were asked 1) for how many hours per week they regularly engage in sports (no sports, less than 1 hour, 1-2 hours, 2-4 hours or more than 4 hours per week) and 2) on how many occasions per week and for how many minutes per occasion they engage in sports or other physical activities in a way that they start to sweat or get out of breath.

From their answers on the first question, participants were categorized into <1 hour, 1-2 hours or >2 hours of sport per week. From the answers to the second question, the time spent on moderate intensity physical activity per week was calculated, and participants were categorized into <1 hour, 1-3 hours or >3 hours of moderate intensity physical activity per week. Eventually, level of physical activity per week was classified as low, medium, and high and participants were allocated to these categories based on the higher categories of both classifications derived from the first and second question.^69^

*Active smoker status*: Active smoking status was assessed by self-administered questionnaire. Participants were asked “Did you use to smoke or do you smoke now?”, if “yes, I smoke now” then the participants were also asked “daily or occasionally?”.

*History of cardiovascular disease*: Positive history of cardiovascular disease was defined as positive lifetime history of physician-diagnosed myocardial infarction or positive lifetime history of physician-diagnosed stroke based on self-reports in the CAPI. Participants were aksed „Has a doctor ever diagnosed you as having a myocardial infarction?” and „Has a doctor ever diagnosed you as having a stroke?“.

*History of cancer*: Lifetime history of physician-diagnosed cancer was assessed by self-reports of participants in the CAPI. Participants were asked „Has a doctor ever diagnosed you as having cancer?“.

*Creatinine*: Serum creatinine was measured by a kinetic alkaline picrate method on an ARCHITECT ci8200 analyser (Abbott Diagnostics, Wiesbaden, Germany). Maximum intra- and inter-assay coefficient of variation were 1.27% and 2.02%, respectively.

*C-reactive protein*: Serum C-reactive protein (CRP) was measured by Immunoturbidimetry on an ARCHITECT ci8200 analyser (Abbott Diagnostics, Wiesbaden, Germany). Maximum intra- and inter-assay coefficient of variation were 3.95% and 4.5%, respectively.

*Measurement of serum 25(OH)D*: The participants were asked to fast for at least 3 h, whereupon venous blood samples were drawn for biochemical analyses. Extra serum was aliquoted and stored at −40°C. Serum 25(OH)D levels were measured from June to September 2005 in the Epidemiological Research Laboratory of the Robert Koch-Institute, using LIAISON chemiluminescence immunoassay (CLIA; DiaSorin Inc., Stillwater, MN, USA). Inter- and intra-assay coefficients of variation for serum 25(OH)D were 11.7% and 9.9%, respectively. The lower detection limit of the assay was 5 nmol/L.^70^

*Body mass index*: Body weight was measured with a calibrated electronic scale (type: SECA) to the nearest 0.1 kg and body height was measured with a levelling board on the electronic scale to the nearest 0.1 cm. Height and weight were measured by trained personnel while the participants wore light clothing and no shoes and body mass index was calculated as weight in kg divided by height in meters squared.^71^

*Blood pressure:* Blood pressure was measured by a physician three times after at least three minutes rest, using a mercury sphygmomanometer (Erkameter 3000, Erka, Bad Jölz, Germany). Mean systolic and diastolic blood pressure was calculated from the second and the third measurements.^72^

*Low-density lipoprotein cholesterol*: LDL cholesterol was estimated using the Friedewald equation.^73^

*Parathyroid hormone*: Serum intact parathyroid hormone concentrations (iPTH) were measured from June to September 2005 in the Epidemiological Research Laboratory of the Robert Koch-Institute, using LIAISON chemiluminescence immunoassay (CLIA) (DiaSorin Inc., Stillwater, MN, USA). Inter- and intra-assay coefficients of variation were 7.2% and 3.7%, respectively. The lower detection limits of the assay was 0.106 pmol/L.^70^

Glucose: Glucose was measured by the glucose oxidase technique using Mega Merck Kits (Merck, Darmstadt, Germany). Inter-assay coefficients of variation ranged between 1.11% and 2.62%, respectively.

**7.** **Longitudinal Aging Study Amsterdam (LASA), first cohort:**

*Present diabetes*: Present diabetes mellitus was defined in those participants that were on glucose lowering drugs, and in those with self-reported diabetes mellitus (“*Do you have diabetes?*”).

*Present arterial hypertension*: Hypertension was defined as systolic blood pressure >140 mmHg and/or diastolic blood pressure >90 mmHg and/or use of anti-hypertensive medication. To obtain information on medication use, the participants had to show all the prescribed drugs he/she used at the moment of the medical interview at home. The names, types and doses were noted by the interviewer. All medication was classified according to the internationally accepted ATC-classification. Medications with ATC-code starting with C02, C07, C08 or C09 were classified as anti-hypertensive medication.

*Physical activity*: Information on physical activity was obtained during each main interview of LASA. The following activities were addressed: walking outdoors, bicycling, light household, heavy household, and two sports activities. In general, the respondents were asked how often and for how long in the previous two weeks they had engaged in each activity. The LASA Physical Activity Questionnaire (LAPAQ) has been validated against seven-day physical activity diaries and seven-day pedometer counts in a subsample of 439 LASA participants. For LASA first and second cohort, a constructed variable was created according to the time per week spent on activities with MET score higher than three: The first level of physical activity encompassed individuals that spent less than one hours per week on activities with MET score higher than three, individuals within the second group spent one to three hours per week and the third level involved individuals with more than three hours. Participants with activities at MET score below three are automatically assigned to the lowest group of physical activity.^74^

*Active smoker status*: In LASA, both current smoking status (never, former, current smoker) and smoking history (age when started smoking, age when stopped smoking) were assessed at each examination during the medical interview (“*Do you smoke?*”).

*History of cardiovascular disease*: Positive history of cardiovascular disease was defined as positive history of myocardial infarction or positive history of stroke. History of myocardial infarction and stroke were self-reported. Questions were “Have you had a myocardial infarction?”, “Did you ever have a stroke or a transient ischemic attack?”, “When did this happen?” and a detailed questionnaire on symptoms left after the event.

*History of cancer*: History of cancer was self-reported. Questions were “*Do you have a tumour or cancer or have you ever had it*?” and “*From which age do you have this disease*?”

*Creatinine*: Serum creatinine in µmol/L was measured using the Jaffe alkaline picrate reaction with a Hitachi 747 analyser.

*C-reactive protein*: The serum concentrations of CRP were determined using a sensitive enzyme-linked immunosorbent assay (ELISA) at Sanquin Research, Amsterdam. CRP concentrations were measured with a sandwich-type ELISA in which polyclonal rabbit anti-CRP antibodies were used as catching antibodies and a biotinylated monoclonal antibody (mAb) against CRP (CLB anti-CRP-2) as the detecting antibody. CRP was measured in duplicate, and averages were used. The detection limit was 0.8 ng/ml, the inter-assay coefficient of variation (CV) was < 4.2% CRP.^75^

*Intake of calcium by supplements*: Calcium intake by supplements was defined as ATC-code A12AA from prescribed medication.

*Measurement of serum 25(OH)D*: Serum 25(OH)D measurements took place in 1997/1998 by a radioimmunoassay (Nichols Diagnostics Capistrano, CA, USA). The inter-assay coefficient of variation was 11 % on average concentrations of 27 and 141 nmol/L. All measurements were performed at the Endocrine Laboratory of the VU University Medical Centre.^43^

*Body mass index*: Body weight was measured without clothes and shoes using a calibrated bathroom balance scale; body height was measured using a stadiometer. BMI was calculated as body weight in kilograms divided by height in meters squared.^76^

*Blood pressure*: Blood pressure (in mmHg) was measured after 5 min of rest at the upper left arm with subjects in a seated position, using an oscillometric blood pressure monitor (Omron Corporation, Tokyo, Japan).

*Low-density lipoprotein*: HDL cholesterol and triglycerides were determined by an enzymatic colorimetric test (Roche diagnostics, Mannheim, Germany). The inter-assay coefficient of variation (CV) was <2.8% for triglycerides, and <6.4% for HDL cholesterol. All laboratory analyses were performed in EDTA plasma samples stored at -80° C, at the Department of Clinical Chemistry of the VUmc in 2005. LDL-cholesterol was calculated as total cholesterol - HDL-cholesterol - VLDL-cholesterol; VLDL-cholesterol was calculated as 0.456 x total triglyceride concentration expressed in mmol/L (Friedewald).1 This was done only for triglyceride concentrations of < 5.0 mmol/L.

The concentration of LDL-cholesterol is usually calculated by the formula of Friedewald et al., because isolation of the LDL fraction requires ultracentrifugation, a technique not generally available in service laboratories. The Friedewald formula provides an adequate estimate of LDL-cholesterol for most fasting specimens but is known to be less reliable as triglyceride concentration increases. Therefore, the formula was only used if triglycerides were < 5.0 mmol/L. LDL cholesterol could not be calculated in 9 subjects, because triglyceride concentrations were 5.0 mmol/L in these subjects.

*Parathyroid hormone*: Parathyroid hormone (pmol L−1) was measured by means of immunoradiometric assay (Incstar Corp., Stillwater, MN, USA), with an inter-assay CV of 12%. The analyses were carried out at the Endocrine Laboratory of the VU University Medical Center.^77^

Glucose: The cutoff of 0.247 mmol/l for fructosamine corresponds to the cutoff of 6.1 mmol/l for fasting plasma glucose in terms of sensitivity and specificity in discriminating subjects with glucose intolerance from subjects with normal glucose tolerance^78^. Because the instructions before blood sampling allowed respondents to take tea and dry toast but no dairy products, we could not guarantee fasting blood samples. Fructosamine is little affected by eating, unlike the plasma glucose concentration. Therefore, we used serum fructosamine as a proxy for plasma glucose. Fructosamine was determined by a colorimetric test (Roche Diagnostics, Mannheim, Germany).

**8.** **Longitudinal Aging Study Amsterdam (LASA), second cohort:**

*The methods of LASA, second cohort, were the same as the methods of the first cohort. Differences between cohorts in covariate assessment are given below.*

*Measurement of serum 25(OH)D*: For all serum 25(OH)D measurements, a radioimmunoassay was used (Diasorin, Stillwater, MN, USA). Blood was collected at the baseline visit, in 2002/2003, and 25(OH)D measurements were performed in 2010/2011. The inter-assay coefficient of variation was 10 % at average concentrations of 30 and 65 nmol/L. All measurements were performed at the Endocrine Laboratory of the VU University Medical Centre.^43^

*Parathyroid hormone*: Parathyroid hormone (pmol L−1) was measured by means of immunometric assay (Luminescence (Architect, Abbott, Laboratories, Diagnostics Division, Abbott park, Chigaco, Illinois USA), with an inter-assay CV of 5%. The analyses were carried out at the Endocrine Laboratory of the VU University Medical Center.

**7. Re-analysis of bio-banked serum/plasma for total 25-hydroxyvitamin D by liquid chromatography-tandem mass spectrometry**

The concentrations of total 25(OH)D (i.e., 25(OH)D2 plus 25(OH)D3) in specifically selected bio-banked serum/plasma (referred to as serum hereafter for ease, unless specified) samples from each of the study populations were measured by the Vitamin D Research Group at University College Cork using a certified LC-MS/MS method, as has been described in detail elsewhere.^12,15,79^ In brief, the LC-MS/MS method measures 25(OH)D2 and 25(OH)D3 in serum as well as the 3-epimer of 25(OH)D3 (3-epi-25(OH)D3), which is not chromatographically resolved from 25(OH)D3 by most routine LC-MS/MS methods. The presence of 3-epimers of 25(OH)D can pose problems for LC-MS/MS methods because the precursor ion and fragmentation patterns are the same as 25(OH)D, thus failure to account for these metabolites can result in overestimation of 25(OH)D3 in particular as the quantitatively more abundant metabolite. The intra-assay CV of the method was <5% for all 25-hydroxyvitamin D metabolites, while the inter-assay CV was <6%. The Vitamin D Research Group is a participant in the VDSP^80^ and is certified by Centers for Disease Control and Prevention’s Vitamin D Standardization Certification Program.^81^ The inter-assay CV for total 25(OH)D was 3.6%. Both the VDSP and the certification program reports total 25(OH)D as well as 25(OH)D2 and 25(OH)D3 using the higher order reference laboratories. In addition, the quality and accuracy of serum total 25(OH)D analysis by the LC-MS/MS in our laboratory is monitored on an ongoing basis by participation in the Vitamin D External Quality Assessment Scheme [DEQAS, Charing Cross Hospital, London, UK].

**8. Applying the VDSP protocol for standardization of serum 25(OH)D data from past surveys to the ODIN WP8 study populations**

The VDSP protocol for standardization of serum 25(OH)D data from past surveys, as employed by some of us previously on the Irish national serum 25(OH)D data^15^, and again in this study, generally entails three steps (outlined in detail elsewhere^11,82^):

1. Use results from the VDSP inter-laboratory comparison study to develop a master equation to convert values based on the current measurement procedure (LC-MS/MS in our case) to the reference measurement procedures at Ghent University and NIST (Protocol 1) [however, this step was not necessary for the present work because the LC-MS/MS method within our Vitamin D Research Group is certified as being standardized to that of the reference measurement procedures; see http://www.cdc.gov/labstandards/pdf/hs/CDC_Certified_Vitamin_D_Procedures.pdf];

2. Re-analysis of a statistically defined sub-sample of the stored (bio-banked) sera from the study population (see below) and an equation is developed to convert all past serum 25(OH)D values to the current measurement procedure (Protocol 2); and

3. The equation is used to convert the previous serum 25(OH)D values to the Vitamin D Research Group certified procedure.

To facilitate Protocol 2, a statistical algorithm for estimating the number of stored samples that need to be re-analysed was developed within the VDSP, and published recently.^11^ The maximum projected sample size of stored serum samples required for this protocol and with this collection of population studies was calculated using procedures for the estimation of the predicted LC-MS/MS-based 25(OH)D value for a given serum 25(OH)D value from the original method of analysis (e.g., immunoassay or LC-MS/MS) with a pre-defined precision of a 95% confidence interval, which have been described elsewhere.^11,12^

Serum samples within each study population separately were selected by first dividing the range of the previous method-based serum 25(OH)D measurements into quartiles, with each quartile being sampled according to a uniform distribution.^79^ This method has been shown, via computer simulations, to be statistically more efficient than uniform random sampling in the entire range.^11^ The selected serum samples for each population study were retrieved from the respective bio-banks and each set shipped to the Vitamin D Research Group, where they were re-analysed for serum total 25(OH)D using LC-MS/MS (as outlined above). Serum total 25(OH)D concentration within each sample were calculated as the sum of respective 25(OH)D2 and 25(OH)D3 concentrations.

The relationship between serum 25(OH)D in the statistical algorithm-defined subset of the sera for each of the WP8 studies separately, as measured by the original method and re-analysed by our traceable LC-MS/MS method, was evaluated using regression analysis, as described elsewhere.^13^ Several best fit lines were evaluated for each data set (as defined by R2 as well as consideration of the residuals plots), and the resulting regression equation which provided the best fit was applied to the entire data set for that population study, as per the VDSP Protocol 2.

**9. Statistical analyses**

The current work displays an individual-participant data (IPD) meta-analysis which was implemented in a one-step approach. In a one-step approach one statistical model is used, while accounting for the clustering between cohort studies, to estimate an overall effect. Rather than the two-step approach, where aggregated data (AD) is pooled first and then effect estimates are combined to a final model, the one-step approach is considered the gold standard of meta-analyses, because the risk for ecological bias is lower than in AD meta-analysis, allows better control of confounding by participant- and study-level covariates, and improves power for detecting interactions and subgroup analyses.^13^ Although it is widely believed that in meta-analysis one- and two-step approaches deliver comparable results^83^, for binary outcomes, e.g. time-to-death and time-to-follow-up in survival analysis, the one-step approach produces more reliable results than the two-step method when few studies or few subjects per study are available.^14^ In survival analysis, the possibility for longer and more up to date follow-up times, and exact inspection of model assumptions may be seen as benefits of conducting an IPD meta-analysis. Parametric models may be more feasible for IPD meta-analysis and serve as an alternative approach to the widely used Cox model, which is a so-called semi-parametric model and makes no assumptions on the distribution of the underlying probability density functions.^18^

Hazard rates were estimated by a one-step meta-analysis model and a parametric Weibull distribution of survival function was used.^84^ We used a mixed model implemented in SAS PROC NLMIXED procedure (SAS Institute Inc., 100 SAS Campus Drive, Cary, USA) with random effects to account for clustering across in cohort studies. The Weibull model itself has been used to derive the Framingham coronary heart disease (CHD) risk equation^84^ and a coronary heart disease risk score for type II diabetes from Tayside data.^85^

For the underlying Weibull distribution two parameters (baseline hazard and scale) must be estimated to describe the underlying distribution of survival function. We used PROC LIFEREG in SAS to assess the parametric distribution. Compared to exponential, log-logistic and lognormal distributions, the Weibull distribution was assessed as best fit in likelihood ratio test. Also, the Weibull distribution assumption was evaluated graphically using log-log survival plots.^86^

Parameter estimation was based on maximum likelihood estimation using a Quasi-Newton optimization and Broyden-Fletcher-Goldfarb-Shanno algorithm.^87^ To account for heterogeneity across study centres, random effects were employed. To numerically integrate over the distribution of random effects, Gauss-Hermite quadrature was used in the likelihood equations and in calculation of conditional intercepts.^88^ The inclusion of random effects in the model was preferred regardless of results of formal testing for statistical heterogeneity.^89^

The present analysis was based on individuals with complete data on age, sex, BMI, season of blood sampling, 25(OH)D concentration, vital status at follow-up and follow-up-time (which was at least 1 day). Participants with missing data were excluded from the analysis and we performed no data imputation.

Continuously distributed data are provided as mean and standard deviation when normally distributed and as median with interquartile ranges (IQR) when skewed. Original and standardized 25(OH)D measurements as well as other covariates at baseline were provided both for each study centre and the whole IPD cohort. Differences between original and standardized 25(OH)D concentrations were assessed by paired Student’s t-test. For comparisons of characteristics across vitamin D status groups, we used ANOVA for continuous and χ^2^ test for categorical data, as appropriate.

For mortality analyses, 25(OH)D was modelled two ways, using a traditional categorical variable approach with seven groups and a restricted cubic splines approach.^6^

The cubic-splines approach was chosen to retain the continuous nature of 25(OH)D values and to calculate hazard ratios (HR) with 95% confidence intervals (CI) at the median value of each group. For HR computation we chose the 25(OH)D group with the lowest mortality risk as the reference.^18,19^ The highest category is generally open-ended and too heterogeneous for being used as a reference group.^8^ Cubic splines were also used to estimate the nadir of the mortality curve, i.e. the concentration of 25(OH)D with the lowest mortality risk. The cubic-splines were determined with five knots at the fifth, 27.5^th^, 50^th^, 72.5^th^, and 95^th^.^22^

The nadir was calculated according to the critical points (zero derivatives) of the spline curve and obtained by a general formula for solving quadratic equations.^6^

Our outcome analyses were adjusted for risk factors of mortality and determinants of vitamin D status. In model 1 we adjusted for age (in years), sex (male/female), and season of blood collection (Spring, Summer, Autumn, and Winter). In model 2, our main statistical model, we additionally adjusted for BMI (in kg/m²). In model 3, we additionally adjusted for diabetes mellitus (yes/no) and arterial hypertension (yes/no), and in model 4 we added history of cancer (yes/no), history of cardiovascular disease (yes/no) and current smoking status (yes/no) as covariates.

**Additional adjustments**

Additional adjustments had model 2 as reference model and included adjustments for supplemental intake of calcium, supplemental intake of vitamin D, physical activity, estimated glomerular filtration rate (eGFR), parathyroid hormone, C-reactive protein, systolic blood pressure, low density lipoprotein cholesterol, and glucose. Participants with missing additional adjustment data were excluded from the respective additional adjustment analysis Additional covariates were not available in every cohort study, so additional covariate adjustments were only performed in the studies that could provide those covariates:

First, supplemental intake of calcium (yes/no) was added to model two. The first additional adjustment analysis was performed in all studies, but DEGS and LASA, second cohort, as no information on supplemental usage was available.

Second, supplemental intake of vitamin D (yes/no) was added to model two in all studies but LASA, first and second cohort, and DEGS, as no information on supplemental usage of vitamin D was available in these studies.

Third, additional adjustment of model two for physical activity (three dummy variables for low, medium and high frequency of physical activity) was processed in all studies but the Aarhus mammography cohort. For sensitivity analysis, we also left out DEGS, as the participants in DEGS revealed a high proportion of younger, physically active individuals.

Fourth, adjustment for estimated glomerular filtration rate (eGFR; in mL/min/1.73m²) was added to model two. The eGFR was calculated from creatinine at baseline visit according to the four-variable Modification of Diet in Renal Disease (MDRD) Study equation and was added to model two in all studies but NHS, as up to the current analysis, no creatinine measurements were available in NHS.

In a fifth analysis, adjustment for parathyroid hormone (in pmol/L) was added to model two in all studies but NHS, Aarhus mammography cohort and LASA, second cohort.

In a sixth analysis, adjustment for C-reactive protein (in mg/L) was added to model two in all studies but NHS, Aarhus mammography cohort and LASA, second cohort.

In a seventh analysis, adjustment for systolic blood pressure (in mm Hg) was added to model two in all studies but the Aarhus mammography cohort, and DEGS.

In an eighth analysis, adjustment for low density lipoprotein cholesterol (in mmol/L) was added to model two in all studies but the Aarhus mammography cohort, and DEGS.

In a ninth analysis, adjustment for glucose (in mmol/L) was added to model two in all studies but the Tromsø Study, Aarhus mammography cohort, and DEGS.

All models on original 25(OH)D were performed without NHS, as NHS had no original 25(OH)D measurements. For standardized 25(OH)D, we computed all models 1) with data of NHS and 2) without data from NHS to provide comparable results between models of original and standardized 25(OH)D measurements.

**Sensitivity Analyses**

Pre-specified subgroup analyses were performed to stratify for risk factors for vitamin D deficiency and mortality. Specifically, we stratified for sex (females/males), age groups (<60 yrs; 60 to <70 yrs; ≥70 yrs), BMI groups (<25 kg/m²; 25 to <30 kg/m²; ≥30 kg/m²), calcium supplementation (yes/no), vitamin D supplementation (yes/no), history of CVD (yes/no), and history of cancer (yes/no). Further sensitivity analyses were restricted to general population cohorts (i.e. all cohorts except LURIC) and to individuals that died > 1 year and > 3 years after baseline examination.

**Secondary outcomes**

Secondary outcomes were cardiovascular mortality and cancer mortality, and were available in all cohort studies except NHS. We utilized traditional Cox proportional hazards and the modified risks regression according to the method of Fine and Gray^23^ to account for competing risks. In brief, proportional hazards may not be satisfied in multi-centre settings, so baseline hazards were allowed to vary across single cohort studies.^26^ The analysis was carried out in one step and clusters of the Tromsø Study, LURIC, the AGES Reykjavik Study, the Aarhus mammography cohort, DEGS and the first LASA cohort. For NHS, no cause-specific mortality was available at the time-point of the analysis.

**Macros**

For the above mentioned usage of restricted cubic splines in regression models, a SAS macro was carried out provided by Harrell FE at the Department of Biostatistics, Vanderbilt University School of Medicine, Nashville, TN, USA.^25^

The competing risk analyses were performed using R Version 3.1.1 and ‘crrSC’ package, Version 1.1 (2013-06-23; Bingqing Zhou and Aurelien Latouche), which is an extension of the ‘cmprsk’ package, Version 2.2-7 (2014-06-17; Bob Gray) to Stratified and Clustered data.^26^

**Heterogeneity**

To assess the size of the random effect and the heterogeneity across studies^19^, we calculated an intra-class correlation coefficient (ICC) and its 95% CI.^89,90^ The ICC was defined as the ratio of between-cluster variance to total variance, while the total variance is the sum of between-cluster and within-cluster variance. The between-cluster variance was estimated by the NLMIXED procedure and the within-cluster variance was set π²/6 to take account of the asymmetric Weibull distribution with its long tail to the right.^91^ The coefficient can be interpreted as the I² measure of inconsistency proposed by Higgins and colleagues, whereby 25% represents small heterogeneity, 50% represents medium heterogeneity, and 75% represents large heterogeneity.^78^

Risk of bias assessment is limited because our work is based on a collaborative meta-analysis of observational studies and not on a study selection based on a systematic literature review.^92,93^ In brief, we consider our work at low risk of study selection bias, publication bias and data avilability bias as our work includes studies that were selected based on pre-specified criteria that are mainly related to data availability. Participant selection bias and attrition bias is also considerd to be relatively low as we detected no significant differences in study parameters for participants who were eligible for analyses and those with missing values. Performance and detection bias as well as outcome or availability bias should also be low due to the clear endpoint definitions of our work.

All statistical tests were two sided using an α level of 0.05 if not otherwise specified. All meta-analyses were conducted with SAS Version 9.2 (SAS Institute Inc., 100 SAS Campus Drive, Cary, USA) or R Version 3.1.1 (2014-07-10; Copyright © The R Foundation for Statistical Computing), data management was performed with SPSS version 20 or higher (IBM Corp. Released 2011. IBM SPSS Statistics for Windows, NY) or MS Excel (Microsoft Excel. Redmond, Washington, USA).

**10. SAS code^94,95^**

*Preparation for cubic splines model with random intercept;

%include "E:\SAS\cubicsplines\survrisk2.sas"; *%DASPLINE macro location;

* SAS macro „%DASPLINE“ was provided by Harrell FE at the Department of Biostatistics, Vanderbilt University School of Medicine, Nashville, TN, USA (Harrell FE., Jr DASPLINE Macro. [January 16th, 2015]; http://biostat.mc.vanderbilt.edu/twiki/pub/Main/SasMacros/survrisk.txt).;

%***daspline*** (Cork25ohd, nk=**5**, **data**=ODINdata.ODINhazard); *5 knots at the fifth, 27.5th, 50th, 72.5th, and 95th percentiles;

**data** tempCork; set ODINdata.ODINhazard; *New output data set;

&_ Cork25ohd;

*The Macro creates 3 additional variables for oldvitd which have to be included into the linear term together with the actual variable in the later model (additional variables are automatically named Cork25ohd1, Cork25ohd2 and Cork25ohd3);

*Model 2 (adjustment for age, sex, body mass index and month of blood sampling);

*Sort database first;

**proc** **sort** data=tempCork; by centerid Cork25ohd; **run**;

ods output;

*Spline model;

*According to John Amrhein, McDougall Scientific Ltd. Introduction to Frailty Models. Paper 1492-2014. AND: Xian Liu. Survival Analysis: Models and Applications. Wiley 1 edition (August 13, 2012).;

TITLE "Model 2: Cubic splines model with random intercept (Cork standardized)";

**proc** **nlmixed** data=tempCork cov ecov tech=quanew update=bfgs;

*Optimization Algorithm = Quasi-Newton optimization and Broyden-Fletcher-Goldfarb-Shanno algorithm (Stedman MR, Lew RA, Losina E, Gagnon DR, Solomon DH, Brookhart MA. A comparison of statistical approaches for physician-randomized trials with survival outcomes. Contemp Clin Trials. 2012 Jan 33(1):104-15.);

*Following statements represent starting values for estimazation process, absolute values are derived from Proc LIFEREG;

parms b0=**12.3208** b01=**0.0189** b1=-**0.0564** b2=**0.2547** b3=-**0.3368** b5=-**0.0578** b6=-**0.3377** b71=**0.0055** b41=**0.0043** b42=-**0.0043** b44=**0.0445** lamda=**1.6118** s2u=**0.0**;

bounds lamda > **0**; *Lamda is set positive;

*Knots for Cork25ohd at (computed as the fifth, 27.5th, 50th, 72.5th, and 95th percentile): 22.10000038 43.02 54.2 65.3 93.30000305;

K1=**22.10000038**; * Knot 1;

K2=**43.02**; * Knot 2;

K3=**54.2**; * Knot 3;

K4=**65.3**; * Knot 4;

K5=**93.30000305**; * Knot 5;

*Reference point and group medians;

Ref1=**83.4**; * Reference point = Median group 5;

G1=**22.8**; * Estimate point 1: Median group 1;

G2=**35.6528300**; * Estimate point 2: Median group 2;

G3=**45.2000008**; * Estimate point 3: Median group 3;

G4=**60.3**; * Estimate point 4: Median group 4;

G5=**83.4**; * Estimate point 5: Median group 5;

G6= **107.0999985**; * Estimate point 6: Median group 6;

G7=**135.0**; * Estimate point 7: Median group 7;

Const_pi = CONSTANT('PI');

linp = b0 + b01*Cork25ohd + b1*Cork25ohd1 + b2*Cork25ohd2 + b3*Cork25ohd3 + b5*age + b6*sex + b71*bmi + b41*monthbaseline11 + b42*monthbaseline12 + b44*monthbaseline14 + u;

* Cork25ohd, Cork25ohd1 … 3 are the variables indicating cubic splines of standardized 25(OH)D. age is the age at baseline visit. sex is the individual participants sex. bmi is the individuals’s body mass index at baseline visit. monthbaseline11 … 14 are the variables indicating month of baseline blood sampling. ;

alpha = exp(-linp);

G_t = exp(-(alpha*timemort)**lamda); *The survival distribution function;

g = lamda*alpha*((alpha*timemort)**(lamda-**1**))*G_t; *The probability density function;

ll = (death=**1**)*log(g) + (death=**0**)*log(G_t);

model timemort ~ general(ll);

random u ~ normal(**0**,s2u) subject=centerid out=EB_corkspline2; *The random effect is normally distributed (s2u is the estimated between-cluster variance);

ESTIMATE 'ICC' s2u /( s2u + ((Const_pi**2)/6)); *The ICC is defined according to Rodrıguez, G. and Elo, I. (2003). Intra-class correlation in random-effects models for binary data. The Stata Journal, 3(1):32-46.;

predict **1**-G_t out=cdf_corkspline2; *The cumulative distribution function;

predict alpha out=alpha_corkspline2;

*Predictions for plot of hazard ratios (the curve has to be further implemented in graphics procedures as PROC GPLOT or PROC SGRENDER);

DROP _kd_;

_kd_= (K5 - K1)****.666666666666** ;

predict exp(-(b01*Cork25ohd

+b1*(max((Cork25ohd-K1)/_kd_,**0**)****3**+((K4-K1)*max((Cork25ohd-K5)/_kd_,**0**)****3**-(K5-K1)*max((Cork25ohd-K4)/_kd_,**0**)****3**)/(K5-K4))

+b2*(max((Cork25ohd-K2)/_kd_,**0**)****3**+((K4-K2)*max((Cork25ohd-K5)/_kd_,**0**)****3**-(K5-K2)*max((Cork25ohd-K4)/_kd_,**0**)****3**)/(K5-K4))

+b3*(max((Cork25ohd-K3)/_kd_,**0**)****3**+((K4-K3)*max((Cork25ohd-K5)/_kd_,**0**)****3**-(K5-K3)*max((Cork25ohd-K4)/_kd_,**0**)****3**)/(K5-K4))

-

(b01*Ref1

+b1*(max((Ref1-K1)/_kd_,**0**)****3**+((K4-K1)*max((Ref1-K5)/_kd_,**0**)****3**-(K5-K1)*max((Ref1-K4)/_kd_,**0**)****3**)/(K5-K4))

+b2*(max((Ref1-K2)/_kd_,**0**)****3**+((K4-K2)*max((Ref1-K5)/_kd_,**0**)****3**-(K5-K2)*max((Ref1-K4)/_kd_,**0**)****3**)/(K5-K4))

+b3*(max((Ref1-K3)/_kd_,**0**)****3**+((K4-K3)*max((Ref1-K5)/_kd_,**0**)****3**-(K5-K3)*max((Ref1-K4)/_kd_,**0**)****3**)/(K5-K4))

)

)*lamda)

out=alpha2_corkspline2;

*Estimates for group medians (hazard ratios);

DROP _kd_;

_kd_= (K5 - K1)****.666666666666**;

estimate "G1" exp(-(b01*G1

+b1*(max((G1-K1)/_kd_,**0**)****3**+((K4-K1)*max((G1-K5)/_kd_,**0**)****3**-(K5-K1)*max((G1-K4)/_kd_,**0**)****3**)/(K5-K4))

+b2*(max((G1-K2)/_kd_,**0**)****3**+((K4-K2)*max((G1-K5)/_kd_,**0**)****3**-(K5-K2)*max((G1-K4)/_kd_,**0**)****3**)/(K5-K4))

+b3*(max((G1-K3)/_kd_,**0**)****3**+((K4-K3)*max((G1-K5)/_kd_,**0**)****3**-(K5-K3)*max((G1-K4)/_kd_,**0**)****3**)/(K5-K4))

-

(b01*Ref1

+b1*(max((Ref1-K1)/_kd_,**0**)****3**+((K4-K1)*max((Ref1-K5)/_kd_,**0**)****3**-(K5-K1)*max((Ref1-K4)/_kd_,**0**)****3**)/(K5-K4))

+b2*(max((Ref1-K2)/_kd_,**0**)****3**+((K4-K2)*max((Ref1-K5)/_kd_,**0**)****3**-(K5-K2)*max((Ref1-K4)/_kd_,**0**)****3**)/(K5-K4))

+b3*(max((Ref1-K3)/_kd_,**0**)****3**+((K4-K3)*max((Ref1-K5)/_kd_,**0**)****3**-(K5-K3)*max((Ref1-K4)/_kd_,**0**)****3**)/(K5-K4))

))*lamda);

estimate "G2" exp(-(b01*G2

+b1*(max((G2-K1)/_kd_,**0**)****3**+((K4-K1)*max((G2-K5)/_kd_,**0**)****3**-(K5-K1)*max((G2-K4)/_kd_,**0**)****3**)/(K5-K4))

+b2*(max((G2-K2)/_kd_,**0**)****3**+((K4-K2)*max((G2-K5)/_kd_,**0**)****3**-(K5-K2)*max((G2-K4)/_kd_,**0**)****3**)/(K5-K4))

+b3*(max((G2-K3)/_kd_,**0**)****3**+((K4-K3)*max((G2-K5)/_kd_,**0**)****3**-(K5-K3)*max((G2-K4)/_kd_,**0**)****3**)/(K5-K4))

-

(b01*Ref1

+b1*(max((Ref1-K1)/_kd_,**0**)****3**+((K4-K1)*max((Ref1-K5)/_kd_,**0**)****3**-(K5-K1)*max((Ref1-K4)/_kd_,**0**)****3**)/(K5-K4))

+b2*(max((Ref1-K2)/_kd_,**0**)****3**+((K4-K2)*max((Ref1-K5)/_kd_,**0**)****3**-(K5-K2)*max((Ref1-K4)/_kd_,**0**)****3**)/(K5-K4))

+b3*(max((Ref1-K3)/_kd_,**0**)****3**+((K4-K3)*max((Ref1-K5)/_kd_,**0**)****3**-(K5-K3)*max((Ref1-K4)/_kd_,**0**)****3**)/(K5-K4))

))*lamda);

estimate "G3" exp(-(b01*G3

+b1*(max((G3-K1)/_kd_,**0**)****3**+((K4-K1)*max((G3-K5)/_kd_,**0**)****3**-(K5-K1)*max((G3-K4)/_kd_,**0**)****3**)/(K5-K4))

+b2*(max((G3-K2)/_kd_,**0**)****3**+((K4-K2)*max((G3-K5)/_kd_,**0**)****3**-(K5-K2)*max((G3-K4)/_kd_,**0**)****3**)/(K5-K4))

+b3*(max((G3-K3)/_kd_,**0**)****3**+((K4-K3)*max((G3-K5)/_kd_,**0**)****3**-(K5-K3)*max((G3-K4)/_kd_,**0**)****3**)/(K5-K4))

-

(b01*Ref1

+b1*(max((Ref1-K1)/_kd_,**0**)****3**+((K4-K1)*max((Ref1-K5)/_kd_,**0**)****3**-(K5-K1)*max((Ref1-K4)/_kd_,**0**)****3**)/(K5-K4))

+b2*(max((Ref1-K2)/_kd_,**0**)****3**+((K4-K2)*max((Ref1-K5)/_kd_,**0**)****3**-(K5-K2)*max((Ref1-K4)/_kd_,**0**)****3**)/(K5-K4))

+b3*(max((Ref1-K3)/_kd_,**0**)****3**+((K4-K3)*max((Ref1-K5)/_kd_,**0**)****3**-(K5-K3)*max((Ref1-K4)/_kd_,**0**)****3**)/(K5-K4))

))*lamda);

estimate "G4" exp(-(b01*G4

+b1*(max((G4-K1)/_kd_,**0**)****3**+((K4-K1)*max((G4-K5)/_kd_,**0**)****3**-(K5-K1)*max((G4-K4)/_kd_,**0**)****3**)/(K5-K4))

+b2*(max((G4-K2)/_kd_,**0**)****3**+((K4-K2)*max((G4-K5)/_kd_,**0**)****3**-(K5-K2)*max((G4-K4)/_kd_,**0**)****3**)/(K5-K4))

+b3*(max((G4-K3)/_kd_,**0**)****3**+((K4-K3)*max((G4-K5)/_kd_,**0**)****3**-(K5-K3)*max((G4-K4)/_kd_,**0**)****3**)/(K5-K4))

-

(b01*Ref1

+b1*(max((Ref1-K1)/_kd_,**0**)****3**+((K4-K1)*max((Ref1-K5)/_kd_,**0**)****3**-(K5-K1)*max((Ref1-K4)/_kd_,**0**)****3**)/(K5-K4))

+b2*(max((Ref1-K2)/_kd_,**0**)****3**+((K4-K2)*max((Ref1-K5)/_kd_,**0**)****3**-(K5-K2)*max((Ref1-K4)/_kd_,**0**)****3**)/(K5-K4))

+b3*(max((Ref1-K3)/_kd_,**0**)****3**+((K4-K3)*max((Ref1-K5)/_kd_,**0**)****3**-(K5-K3)*max((Ref1-K4)/_kd_,**0**)****3**)/(K5-K4))

))*lamda);

estimate "G5" exp(-(b01*G5

+b1*(max((G5-K1)/_kd_,**0**)****3**+((K4-K1)*max((G5-K5)/_kd_,**0**)****3**-(K5-K1)*max((G5-K4)/_kd_,**0**)****3**)/(K5-K4))

+b2*(max((G5-K2)/_kd_,**0**)****3**+((K4-K2)*max((G5-K5)/_kd_,**0**)****3**-(K5-K2)*max((G5-K4)/_kd_,**0**)****3**)/(K5-K4))

+b3*(max((G5-K3)/_kd_,**0**)****3**+((K4-K3)*max((G5-K5)/_kd_,**0**)****3**-(K5-K3)*max((G5-K4)/_kd_,**0**)****3**)/(K5-K4))

-

(b01*Ref1

+b1*(max((Ref1-K1)/_kd_,**0**)****3**+((K4-K1)*max((Ref1-K5)/_kd_,**0**)****3**-(K5-K1)*max((Ref1-K4)/_kd_,**0**)****3**)/(K5-K4))

+b2*(max((Ref1-K2)/_kd_,**0**)****3**+((K4-K2)*max((Ref1-K5)/_kd_,**0**)****3**-(K5-K2)*max((Ref1-K4)/_kd_,**0**)****3**)/(K5-K4))

+b3*(max((Ref1-K3)/_kd_,**0**)****3**+((K4-K3)*max((Ref1-K5)/_kd_,**0**)****3**-(K5-K3)*max((Ref1-K4)/_kd_,**0**)****3**)/(K5-K4))

))*lamda);

estimate "G6" exp(-(b01*G6

+b1*(max((G6-K1)/_kd_,**0**)****3**+((K4-K1)*max((G6-K5)/_kd_,**0**)****3**-(K5-K1)*max((G6-K4)/_kd_,**0**)****3**)/(K5-K4))

+b2*(max((G6-K2)/_kd_,**0**)****3**+((K4-K2)*max((G6-K5)/_kd_,**0**)****3**-(K5-K2)*max((G6-K4)/_kd_,**0**)****3**)/(K5-K4))

+b3*(max((G6-K3)/_kd_,**0**)****3**+((K4-K3)*max((G6-K5)/_kd_,**0**)****3**-(K5-K3)*max((G6-K4)/_kd_,**0**)****3**)/(K5-K4))

-

(b01*Ref1

+b1*(max((Ref1-K1)/_kd_,**0**)****3**+((K4-K1)*max((Ref1-K5)/_kd_,**0**)****3**-(K5-K1)*max((Ref1-K4)/_kd_,**0**)****3**)/(K5-K4))

+b2*(max((Ref1-K2)/_kd_,**0**)****3**+((K4-K2)*max((Ref1-K5)/_kd_,**0**)****3**-(K5-K2)*max((Ref1-K4)/_kd_,**0**)****3**)/(K5-K4))

+b3*(max((Ref1-K3)/_kd_,**0**)****3**+((K4-K3)*max((Ref1-K5)/_kd_,**0**)****3**-(K5-K3)*max((Ref1-K4)/_kd_,**0**)****3**)/(K5-K4))

))*lamda);

estimate "G7" exp(-(b01*G7

+b1*(max((G7-K1)/_kd_,**0**)****3**+((K4-K1)*max((G7-K5)/_kd_,**0**)****3**-(K5-K1)*max((G7-K4)/_kd_,**0**)****3**)/(K5-K4))

+b2*(max((G7-K2)/_kd_,**0**)****3**+((K4-K2)*max((G7-K5)/_kd_,**0**)****3**-(K5-K2)*max((G7-K4)/_kd_,**0**)****3**)/(K5-K4))

+b3*(max((G7-K3)/_kd_,**0**)****3**+((K4-K3)*max((G7-K5)/_kd_,**0**)****3**-(K5-K3)*max((G7-K4)/_kd_,**0**)****3**)/(K5-K4))

-

(b01*Ref1

+b1*(max((Ref1-K1)/_kd_,**0**)****3**+((K4-K1)*max((Ref1-K5)/_kd_,**0**)****3**-(K5-K1)*max((Ref1-K4)/_kd_,**0**)****3**)/(K5-K4))

+b2*(max((Ref1-K2)/_kd_,**0**)****3**+((K4-K2)*max((Ref1-K5)/_kd_,**0**)****3**-(K5-K2)*max((Ref1-K4)/_kd_,**0**)****3**)/(K5-K4))

+b3*(max((Ref1-K3)/_kd_,**0**)****3**+((K4-K3)*max((Ref1-K5)/_kd_,**0**)****3**-(K5-K3)*max((Ref1-K4)/_kd_,**0**)****3**)/(K5-K4))

))*lamda);

*Nadir computation;

*Reference: Sempos CT, Durazo-Arvizu RA, Dawson-Hughes B, et al. Is there a reverse J-shaped association between 25-hydroxyvitamin D and all-cause mortality? Results from the U.S. nationally representative NHANES. J Clin Endocrinol Metab 2013; 98(7): 3001-9.;

b1_cs=**3***(((K5-K1)/_kd_)+(**3***((K4-K5)/_kd_)-(K5-K1)*(**3***(K5-K4)/_kd_))/(K5-K4));

b2_cs=**3***(((K5-K2)/_kd_)+(**3***((K4-K5)/_kd_)-(K5-K2)*(**3***(K5-K4)/_kd_))/(K5-K4));

b3_cs=**3***(((K5-K3)/_kd_)+(**3***((K4-K5)/_kd_)-(K5-K3)*(**3***(K5-K4)/_kd_))/(K5-K4));

b1_cs2=**3***(((K5-K1)/_kd_)****2**+(**3***((K4-K5)/_kd_)****2**-(K5-K1)*(**3***(K5-K4)/_kd_)****2**)/(K5-K4));

b2_cs2=**3***(((K5-K2)/_kd_)****2**+(**3***((K4-K5)/_kd_)****2**-(K5-K2)*(**3***(K5-K4)/_kd_)****2**)/(K5-K4));

b3_cs2=**3***(((K5-K3)/_kd_)****2**+(**3***((K4-K5)/_kd_)****2**-(K5-K3)*(**3***(K5-K4)/_kd_)****2**)/(K5-K4));

A = (**3***(b1+b2+b3));

B = (-**6***(b1*b1_cs+b2*b2_cs+b3*b3_cs));

C = (b01 + **3***(b1*b1_cs2+b2*b2_cs2+b3*b3_cs2));

*The critical points (zero derivative) are obtained by a general quadratic function;

estimate "Nadir" ((-B+sqrt((B****2**)-(**4***A*C)))/(**2***A))*lamda*(-**1**);

b1_cs3=**3***(((K1-K5)/_kd_)+(**3***((K4-K5)/_kd_)-(K5-K1)*(**3***(K5-K4)/_kd_))/(K5-K4));

b2_cs3=**3***(((K2-K5)/_kd_)+(**3***((K4-K5)/_kd_)-(K5-K2)*(**3***(K5-K4)/_kd_))/(K5-K4));

b3_cs3=**3***(((K3-K5)/_kd_)+(**3***((K4-K5)/_kd_)-(K5-K3)*(**3***(K5-K4)/_kd_))/(K5-K4));

b1_cs32=**3***(((K1-K5)/_kd_)****2**+(**3***((K4-K5)/_kd_)****2**-(K5-K1)*(**3***(K5-K4)/_kd_)****2**)/(K5-K4));

b2_cs32=**3***(((K2-K5)/_kd_)****2**+(**3***((K4-K5)/_kd_)****2**-(K5-K2)*(**3***(K5-K4)/_kd_)****2**)/(K5-K4));

b3_cs32=**3***(((K3-K5)/_kd_)****2**+(**3***((K4-K5)/_kd_)****2**-(K5-K3)*(**3***(K5-K4)/_kd_)****2**)/(K5-K4));

A_2 = (**3***(b1+b2+b3));

B_2 = (-**6***(b1*b1_cs3+b2*b2_cs3+b3*b3_cs3));

C_2 = (b01 + **3***(b1*b1_cs32+b2*b2_cs32+b3*b3_cs32));

estimate "Nadir2" ((-B_2+sqrt((B_2****2**)-(**4***A_2*C_2)))/(**2***A_2))*lamda*(-**1**);

**run**;

title;

ods output close;

**11. Additional funding sources**

**A. Design and conduct of the study**

LR has received founding from the Danish Council for Independent Research in Medical Sciences (FSS), The Danish Dairy Foundation, The Novo Nordic Foundation, and the Toyota Foundation.

*Tromsø Study*: None.

*Ludwigshafen RIsk and Cardiovascular Health Study*: None.

*Age, Gene/Environment Susceptibility Reykjavik Study*: This study has been funded by NIH contract N01-AG012100, the NIA Intramural Research Program, Hjartavernd (the Icelandic Heart Association), and the Althingi (the Icelandic Parliament), an Intramural Research Program Award (ZIAEY000401) from the National Eye Institute, an award from the National Institute on Deafness and Other Communication Disorders (NIDCD) Division of Scientific Programs (IAA Y2-DC_1004-02), The study is approved by the Icelandic National Bioethics Committee, VSN: 00-063.

*The New Hoorn Study*: The New Hoorn Study was funded by VUmc.

*Aarhus Mammography Cohort Study*: The study was supported by a grant (# 09-070940) from the Danish Council for Independent Research in Medical Sciences (FSS) and was founded by the Department of Experimental Clinical Research, the Aarhus University Research Foundation, Helga & Peter Kornings foundation, Frits, Georg & Marie Cecilie Gluds foundation, Aase & Ejnar Danielsens foundation and Eva & Henry Fraenkels memorial foundation

*German Health Interview and Examination Survey for Adults*: None.

*Longitudinal Aging Study Amsterdam*: The Longitudinal Aging Study Amsterdam is largely supported by a grant from the Netherlands Ministry of Health Welfare and Sports, Directorate of Long-Term Care.

**B. Collection, management, analysis, and interpretation of the data**

A license of SAS Analytics was provided by the Steiermärkische Krankenanstaltengesellschaft m.b.H. (KAGES), Graz, Austria.

**C. Preparation, review, or approval of the manuscript**

None.

**12. Acknowledgements**

We want to thank the participants of each single studies for their participation and willingness to be part of a study cohort.

We want to thank Regina Riedl and Sereina Herzog at the Institute for Medical Informatics, Statistics and Documentation Medical University of Graz, for their critical check of the underlying statistics of the manuscript, who received no compensation for their contributions.

*Age, Gene/Environment Susceptibility Reykjavik Study*: The researchers are indebted to the participants for their willingness to participate in the study.

Martin Gaksch and Stefan Pilz had full access to all of the data in the study and take responsibility for the integrity of the data and the accuracy of the data analysis. The manuscript is an honest, accurate, and transparent account of the study being reported; no important aspects of the study have been omitted; any discrepancies from the study as planned (and, if relevant, registered) have been explained.

**13. Author's contribution**

All authors substantially contributed to the conception of the work and critically revised the manuscript. Authors contributed furthermore to:

Substantial effort in drafting and writing the manuscript was made by S.P.

Substantial effort in data analysis was made by M.G.A., S.P., .A.B., G.B.M.M., C.T.S., and R.A.D.A.

Substantial effort in standardization of 25(OH)D measurements were made by Z.S., K.G.D., K.D.C., and M.K.

Substantial effort in interpretation of the data was made by M.G.R., S.P., R.O.J., R.A.J., E.B.M., G.E., E.F.G., T.B.H., M.F.C., F.R., J.W.J.B., E.V.R., M.A.B., G.B.M.M., C.S.N., K.M.A.S., N.M.S., I.A.B., P.L., C.T.S., K.G.D., K.D.C., and M.K.

Substantial effort in the design of the work was made by S.P., R.O.J., R.A.J., G.E., V.G., L.R., N.M.S., P.L., K.D.C., and M.K.

Substantial effort in data collection was made by M.G.A., S.P., R.O.J., G.G., R.A.J., E.B.M., T.W., I.N., M.L.L., M.E.K., W.M., G.E., E.F.G., V.G., T.A., T.B.H., M.F.C., F.R., J.W.J.B., E.V.R., G.N., J.M.D., D.G.L., L.R., M.A.B., G.B.M.M., C.S.N., K.M.A.S., N.M.S., I.A.B., P.L., Z.S., K.G.D., K.D.C., and M.K.

**14. PRISMA-IPD^96^**

**Title**

1. Title

Identify the report as a systematic review and meta-analysis of individual participant data (Reported in main article on page 1).

**Abstract**

2. Structured summary

Provide a structured summary including as applicable:

(Reported in main article on page 5)

Background: State research question and main objectives, with information on participants, interventions, comparators, and outcomes.

Methods: Report eligibility criteria; data sources including dates of last bibliographic search or elicitation, noting that IPD were sought; methods of assessing risk of bias.

Results: Provide number and type of studies and participants identified and number (%) obtained; summary effect estimates for main outcomes (benefits and harms) with confidence intervals and measures of statistical heterogeneity. Describe the direction and size of summary effects in terms meaningful to those who would put findings into practice.

Discussion: State main strengths and limitations of the evidence, general interpretation of the results, and any important implications.

Other: Report primary funding source, registration number, and registry name for the systematic review and IPD meta-analysis.

**Introduction**

3. Rationale

Describe the rationale for the review in the context of what is already known (Reported in main article on page 8).

4. Objectives

Provide an explicit statement of the questions being addressed with reference, as applicable, to participants, interventions, comparisons, outcomes, and study design (PICOS). Include any hypotheses that relate to particular types of participant-level subgroups (Reported in main article on page 8).

**Methods**

5. Protocol and registration

Indicate if a protocol exists and where it can be accessed. If available, provide registration information including registration number and registry name. Provide publication details, if applicable. (Reported in main article on page 9; Protocol published: Clinicaltrials.gov number NCT02438488).

6. Eligibility criteria

Specify inclusion and exclusion criteria including those relating to participants, interventions, comparisons, outcomes, study design, and characteristics (eg, years when conducted, required minimum follow-up). Note whether these were applied at the study or individual level, ie, whether eligible participants were included (and ineligible participants excluded) from a study that included a wider population than specified by the review inclusion criteria. The rationale for criteria should be stated (Reported in main article on page 9 and 11 and appendix, section 1).

7. Identifying studies—information sources

Describe all methods of identifying published and unpublished studies including, as applicable: which bibliographic databases were searched with dates of coverage; details of any hand searching including of conference proceedings; use of study registers and agency or company databases; contact with the original research team and experts in the field; open advertisements; and surveys. Give the date of last search or elicitation (The underlying analysis is a collaborative meta analysis and does not include a systematic review; study identification methods are reported in main article on page 9 and appendix, section 1).

8. Identifying studies—search

Present the full electronic search strategy for at least 1 database, including any limits used, such that it could be repeated (The underlying analysis is a collaborative meta analysis and does not include a systematic review; study identification methods are reported in main article on page 9 and appendix, section 1).

9. Study selection processes

State the process for determining which studies were eligible for inclusion (Reported in main article on pages 9, 10 and appendix, section 1).

10. Data collection processes

Describe how IPD were requested, collected, and managed, including any processes for querying and confirming data with investigators. If IPD were not sought from any eligible study, the reason for this should be stated (for each such study). If applicable, describe how any studies for which IPD were not available were dealt with. This should include whether, how, and what aggregate data were sought or extracted from study reports and publications (such as extracting data independently in duplicate) and any processes for obtaining and confirming these data with investigators (reported in appendix, section 3).

11. Data items

Describe how the information and variables to be collected were chosen. List and define all study-level and participant-level data that were sought, including baseline and follow-up information. If applicable, describe methods of standardizing or translating variables within the IPD data sets to ensure common scales or measurements across studies. IPD integrity A1 Describe what aspects of IPD were subject to data checking (such as sequence generation, data consistency and completeness, baseline imbalance) and how this was done (Reported in appendix, section 4 and 5).

12. Risk of bias assessment in individual studies

Describe methods used to assess risk of bias in the individual studies and whether this was applied separately for each outcome. If applicable, describe how findings of IPD checking were used to inform the assessment. Report if and how risk of bias assessment was used in any data synthesis (Reported in appendix section 9).

13. Specification of outcomes and effect measures

State all treatment comparisons of interest. State all outcomes addressed and define them in detail. State whether they were prespecified for the review and, if applicable, whether they were primary/main or secondary/additional outcomes. Give the principal measures of effect (such as risk ratio, hazard ratio, difference in means) used for each outcome (Reported in main article on page 11).

14. Synthesis methods

Describe the meta-analysis methods used to synthesize IPD. Specify any statistical methods and models used. Issues should include (but are not restricted to):

(Reported in main article on page 13/appendix section 9)

• Use of a 1-stage or 2-stage approach

• How effect estimates were generated separately within each study and combined across studies (where applicable)

• Specification of 1-stage models (where applicable) including how clustering of participants within studies was accounted for

• Use of fixed- or random-effects models and any other model assumptions, such as proportional hazards

• How (summary) survival curves were generated (where applicable)

• Methods for quantifying statistical heterogeneity (such as I2 and τ2)

• How studies providing IPD and not providing IPD were analyzed together (where applicable)

• How missing data within the IPD were dealt with (where applicable).

A2. Exploration of variation in effects

If applicable, describe any methods used to explore variation in effects by study- or participant-level characteristics (such as estimation of interactions between effect and covariates). State all participant-level characteristics that were analyzed as potential effect modifiers and whether these were prespecified (Reported in main article on page 13/appendix section 9).

15. Risk of bias across studies

Specify any assessment of risk of bias relating to the accumulated body of evidence, including any pertaining to not obtaining IPD for particular studies, outcomes, or other variables (Reported in appendix section 9).

16. Additional analyses

Describe methods of any additional analyses, including sensitivity analyses. State which of these were prespecified (Reported in main article on page 12 and 13).

**Results**

17. Study selection and IPD obtained

Give numbers of studies screened, assessed for eligibility, and included in the systematic review with reasons for exclusions at each stage. Indicate the number of studies and participants for which IPD were sought and for which IPD were obtained. For those studies for which IPD were not available, give the numbers of studies and participants for which aggregate data were available. Report reasons for nonavailability of IPD. Include a flow diagram (The underlying analysis is a collaborative meta analysis and does not include a systematic review; study identification methods are reported in main article on page 9 and appendix, section 1).

18. Study characteristics

For each study, present information on key study and participant characteristics (such as description of interventions, numbers of participants, demographic data, unavailability of outcomes, funding source, and if applicable duration of follow-up). Provide (main) citations for each study. Where applicable, also report similar study characteristics for any studies not providing IPD (Reported in main article on page 15/appendix section 2).

A3. IPD integrity

Report any important issues identified in checking IPD or state that there were none (No issues identified).

19. Risk of bias within studies

Present data on risk of bias assessments. If applicable, describe whether data checking led to the up-weighting or down-weighting of these assessments. Consider how any potential bias affects the robustness of meta-analysis conclusions (Reported in appendix section 9 as well as section 2 with related references).

20. Results of individual Studies

For each comparison and for each main outcome (benefit or harm), for each individual study report the number of eligible participants for which data were obtained and show simple summary data for each intervention group (including, where applicable, the number of events), effect estimates, and confidence intervals. These may be tabulated or included on a forest plot (Reported in supplementary tables).

21. Results of syntheses

Present summary effects for each meta-analysis undertaken, including confidence intervals and measures of statistical heterogeneity. State whether the analysis was prespecified, report the numbers of studies and participants and, where applicable, report the number of events on which it is based. When exploring variation in effects due to patient or study characteristics, present summary interaction estimates for each characteristic examined, including confidence intervals and measures of statistical heterogeneity. State whether the analysis was prespecified. State whether any interaction is consistent across trials. Provide a description of the direction and size of effect in terms meaningful to those who would put findings into practice (Reported in main article on page 15).

22. Risk of bias across studies

Present results of any assessment of risk of bias relating to the accumulated body of evidence, including any pertaining to the availability and representativeness of available studies, outcomes, or other variables (Reported in appendix section 9).

23. Additional analyses

Give results of any additional analyses (eg, sensitivity analyses). If applicable, this should also include any analyses that incorporate aggregate data for studies that do not have IPD. If applicable, summarize the main meta-analysis results following the inclusion or exclusion of studies for which IPD were not available (Reported in main article on page 15/supplementary tables).

**Discussion**

24. Summary of evidence

Summarize the main findings, including the strength of evidence for each main outcome (Reported in main article on page 15).

25. Strengths and limitations

Discuss any important strengths and limitations of the evidence, including the benefits of access to IPD and any limitations arising from IPD that were not available (Reported in main article on page 18).

26. Conclusions

Provide a general interpretation of the findings in the context of other evidence (Reported in main article on page 18).

A4. Implications

Consider relevance to key groups (such as policy makers, service providers, and service users). Consider implications for future research (Reported in main article on page 19).

**Funding**

27. Funding

Describe sources of funding and other support (such as supply of IPD) and the role in the systematic review of those providing such support (Reported in main article on page 7, appendix section 11).

**15.** References of Appendix

1. Ross AC, Manson JE, Abrams SA, et al. [The 2011 report on dietary reference intakes for calcium and vitamin D from the Institute of Medicine: what clinicians need to know.](http://www.ncbi.nlm.nih.gov/pubmed/21118827) J Clin Endocrinol Metab 2011;96(1):53-8.

2. Holick MF, Binkley NC, Bischoff-Ferrari HA, et al. [Evaluation, treatment, and prevention of vitamin D deficiency: an Endocrine Society clinical practice guideline.](http://www.ncbi.nlm.nih.gov/pubmed/21646368) J Clin Endocrinol Metab 2011;96(7):1911-30.

3. Autier P, Boniol M, Pizot C, Mullie P. Vitamin D status and ill health: a systematic

review. Lancet Diabetes Endocrinol 2014;2(1):76-89.

4. Theodoratou E, Tzoulaki I, Zgaga L, Ioannidis JP. Vitamin D and multiple health outcomes: umbrella review of systematic reviews and meta-analyses of observational studies and randomised trials. BMJ 2014;348:g2035.

5. Chowdhury R, Kunutsor S, Vitezova A, et al. Vitamin D and risk of cause specific death: systematic review and meta-analysis of observational cohort and randomised intervention studies. BMJ 2014;348:g1903.

6. Sempos CT, Durazo-Arvizu RA, Dawson-Hughes B, et al. [Is there a reverse J-shaped association between 25-hydroxyvitamin D and all-cause mortality? Results from the U.S. nationally representative NHANES.](http://www.ncbi.nlm.nih.gov/pubmed/23666975) J Clin Endocrinol Metab 2013;98(7):3001-9.

7. Schöttker B, Jorde R, Peasey A, et al. Vitamin D and mortality: meta-analysis of individual participant data from a large consortium of cohort studies from Europe and the United States. BMJ 2014;348:g3656.

8. Zittermann A, Iodice S, Pilz S, Grant WB, Bagnardi V, Gandini S. Vitamin D deficiency and mortality risk in the general population: a meta-analysis of prospective cohort studies. Am J Clin Nutr 2012;95(1):91-100.

9. Binkley N, Krueger D, Cowgill CS, et al. [Assay variation confounds the diagnosis of hypovitaminosis D: a call for standardization.](http://www.ncbi.nlm.nih.gov/pubmed/15240586) J Clin Endocrinol Metab 2004;89(7):3152-7.

10. Schöttker B, Jansen EH, Haug U, Schomburg L, Köhrle J, Brenner H. [Standardization of misleading immunoassay based 25-hydroxyvitamin D levels with liquid chromatography tandem-mass spectrometry in a large cohort study.](http://www.ncbi.nlm.nih.gov/pubmed/23133659) PLoS One 2012;7(11):e48774.

11. Binkley N, Sempos CT. Vitamin D Standardization Program (VDSP). [Standardizing vitamin D assays: the way forward.](http://www.ncbi.nlm.nih.gov/pubmed/24737265) J Bone Miner Res 2014;29(8):1709-14.

12. Cashman KD, Kiely M, Kinsella M, et al. [Evaluation of Vitamin D Standardization Program protocols for standardizing serum 25-hydroxyvitamin D data: a case study of the program's potential for national nutrition and health surveys.](http://www.ncbi.nlm.nih.gov/pubmed/23615829) Am J Clin Nutr 2013;97(6):1235-42.

13. Riley RD, Lambert PC, Abo-Zaid G. [Meta-analysis of individual participant data: rationale, conduct, and reporting.](http://www.ncbi.nlm.nih.gov/pubmed/20139215) BMJ 2010;340:c221.

14. Debray TP, Moons KG, Abo-Zaid GM, Koffijberg H, Riley RD. Individual participant data meta-analysis for a binary outcome: one-stage or two-stage? PLoS One 2013;8(4):e60650.

15. Cashman KD, Dowling KG, Škrabáková Z, et al. Vitamin D deficiency in Europe: pandemic? Am J Clin Nutr 2016 Feb 10. pii:ajcn120873.

16. Hicks KA, Hung HMJ, Mahaffey KW, et al. Standardized definitions for end point events in cardiovascular trials. (Accessed June 9^th^, 2014, at http://www.clinpage.com/images/uploads/endpoint-defs_11-16-2010.pdf).

17. Riley RD. Commentary: like it and lump it? Meta-analysis using individual participant data. Int J Epidemiol 2010;39(5):1359-61.

18. Royston P. Flexible parametric alternatives to the Cox model, and more. Stata J 2001; 1:1-28.

19. Abo-Zaid G, Guo B, Deeks JJ, et al. Individual participant data meta-analyses should not ignore clustering. J Clin Epidemiol 2013;66(8):865-873.

20. Liu Q. A two-stage hierarchical regression model for meta-analysis of epidemiologic nonlinear dose-response data. Comput Stat Data Anal 2009;53:4157-67.

21. Bagnardi V. Flexible meta-regression functions for modeling aggregate dose-response data, with an application to alcohol and mortality. Am J Epidemiol 2004;159:1077-86.

22. Harrell FE. Regression Modeling Strategies with Applications to Linear Models, Logistic egression and Survival Analysis. New York: Springer-Verlag 2001.

23. Jason PF, Gray RJ. A proportional hazards model for the subdistribution of a competing risk. J Am Stat Assoc 1999;94(446):496-509.

24. Thomas D, Radji S, Benedetti A. Systematic review of methods for individual patient data meta- analysis with binary outcomes. BMC Med Res Methodol 2014;14:79.

25. Harrell FE., Jr, DASPLINE Macro for SAS. (Accessed January 16^th^, 2015, at <http://biostat.mc.vanderbilt.edu/twiki/pub/Main/SasMacros/survrisk.txt>)

26. Zhou B, Latouche A, Rocha V, Fine J. Competing risks regression for stratified data. Biometrics 2011;67(2):661-70.

27. Bolland MJ, Grey A, Gamble GD, Reid IR. The effect of vitamin D supplementation on skeletal, vascular, or cancer outcomes: a trial sequential meta-analysis. Lancet Diabetes

Endocrinol 2014;2(4):307-20.

28. Bjelakovic G, Gluud LL, Nikolova D, et al. [Vitamin D supplementation for prevention of mortality in adults.](http://www.ncbi.nlm.nih.gov/pubmed/24414552) Cochrane Database Syst Rev 2014;1:CD007470.

29. Rejnmark L, Avenell A, Masud T, et al. [Vitamin D with calcium reduces mortality: patient level pooled analysis of 70,528 patients from eight major vitamin D trials.](http://www.ncbi.nlm.nih.gov/pubmed/22605432) J Clin Endocrinol Metab 2012;97(8):2670-81.

30. Kupferschmidt K. Uncertain verdict as vitamin D goes on trial. Science 2012;337(6101):1476-8.30

31. Pilz S, Rutters F, Dekker JM. [Disease prevention: vitamin D trials.](http://www.ncbi.nlm.nih.gov/pubmed/23161977) Science 2012;338(6109):883.

32. Amrein K, Schnedl C, Holl A, et al. [Effect of high-dose vitamin D3 on hospital length of stay in critically ill patients with vitamin D deficiency: the VITdAL-ICU randomized clinical trial.](http://www.ncbi.nlm.nih.gov/pubmed/25268295) JAMA 2014;312(15):1520-30.

33. Kiely M, Cashman KD, on behalf of the ODIN Consortium. The ODIN project: development of food-based approaches for prevention of vitamin D deficiency throughout life. *Nutr Bull.* 2015; 40:235-246.

34. Jorde R, Schirmer H, Wilsgaard T, et al. Polymorphisms related to the serum 25-hydroxyvitamin D level and risk of myocardial infarction, diabetes, cancer and mortality. The Tromso Study. *PLoS One.* 2012;7(5):e37295.

35. Jacobsen BK, Eggen AE, Mathiesen EB, Wilsgaard T, Njølstad I. Cohort profile: the Tromso Study. *Int J Epidemiol.* 2012;41(4):961-7.

36. Winkelmann BR, Marz W, Boehm BO, et al. Rationale and design of the LURIC study--a resource for functional genomics, pharmacogenomics and long-term prognosis of cardiovascular disease. *Pharmacogenomics.* 2001;2:S1-73.

37. Harris TB, Launer LJ, Eiriksdottir G, et al. Age, Gene/Environment Susceptibility-Reykjavik Study: multidisciplinary applied phenomics. *Am J Epidemiol.* 2007;165(9):1076-87.

38. van 't Riet E, Alssema M, Rijkelijkhuizen JM, Kostense PJ, Nijpels G, Dekker JM. Relationship between A1C and glucose levels in the general Dutch population: the new Hoorn study. *Diabetes Care.* 2010;33(1):61-6.

39. Rejnmark L, Tietze A, Vestergaard P, et al. Reduced prediagnostic 25-hydroxyvitamin D levels in women with breast cancer: a nested case-control study. *Cancer Epidemiol Biomarkers Prev* 2009;18(10):2655-60.

40. Scheidt-Nave C, Kamtsiuris P, Gößwald A, et al. German health interview and examination survey for adults (DEGS) - design, objectives and implementation of the first data collection wave. *BMC Public Health.* 2012;12:730.

41. Bellach BM, Knopf H, Thefeld W. The German Health Survey 1997/98 (article in German) *Gesundheitswesen.* 1998;60(Suppl 2):59-68.

42. Huisman M, Poppelaars J, van der Horst M, et al. Cohort profile: the Longitudinal Aging Study Amsterdam. *Int J Epidemiol.* 2011;40(4):868-76.

43. van Schoor NM, Knol DL, Deeg DJ, Peters FP, Heijboer AC, Lips P. Longitudinal changes and seasonal variations in serum 25-hydroxyvitamin D levels in different age groups: results of the Longitudinal Aging Study Amsterdam. *Osteoporos Int.* 2014;25(5):1483-91.

44. Metcalfe A, Neudam A, Forde S, et al. Case definitions for acute myocardial infarction in administrative databases and their impact on in-hospital mortality rates. *Health Serv Res.* 2013, Feb;48(1):290-318.

45. Hennessy S, Leonard CE, Freeman CP, et al. Validation of diagnostic codes for outpatient-originating sudden cardiac death and ventricular arrhythmia in Medicaid and Medicare claims data. *Pharmacoepidemiol Drug Saf*. 2010, Jun;19(6):555-562.

46. Quach S, Blais C, Quan H. Administrative data have high variation in validity for recording heart failure. *Can J Cardiol.* 2010, Oct;26(8):306-12.

47. Kokotailo RA, Hill MD. Coding of stroke and stroke risk factors using international classification of diseases, revisions 9 and 10. *Stroke.* 2005, Aug;36(8):1776-81.

48. Freedman DM, Looker AC, Chang SC, Graubard BI. Prospective study of serum vitamin D and cancer mortality in the United States. *J Natl Cancer Inst.* 2007;99(21):1594-602.

49. Kvamme JM, Holmen J, Wilsgaard T, Florholmen J, Midthjell K, Jacobsen BK. Body mass index and mortality in elderly men and women: the Tromsø and HUNT studies. *J Epidemiol Community Health.* 2012;66(7):611-7.

50. Murr C, Grammer TB, Kleber ME, Meinitzer A, März W, Fuchs D. Low serum tryptophan predicts higher mortality in cardiovascular disease. *Eur J Clin Invest.* 2015;45(3):247-54.

51. Statistics Iceland. Births and deaths . [Internet] Statistics Iceland, Borgartúni 21a, 150 Reykjavík. (updated 2013; cited 2015 Apr 8, Available at http://www.statice.is/Statistics/Population/Births-and-deaths).

52. Rejnmark L, Vestergaard P, Heickendorff L, Mosekilde L. Determinants of plasma PTH and their implication for defining a reference interval. *Clin Endocrinol. (Oxf)* 2011;74:37-43.

53. Wolf IK, Busch M, Lange M, et al. [Mortality follow-up of the German Health Interview and Examination Survey for Adults (DEGS). Methods and first results]. *Bundesgesundheitsbl.* 2014;57:1331-1337.

54. de Jongh RT, Lips P, Rijs KJ, et al. Associations between vitamin D receptor genotypes and mortality in a cohort of older Dutch individuals. *Eur J Endocrinol.* 2011;164(1):75-82.

55. Mancia G et al. Task Force Members. 2013 ESH/ESC Guidelines for the management of arterial hypertension: the Task Force for the management of arterial hypertension of the European Society of Hypertension (ESH) and of the European Society of Cardiology (ESC); *J Hypertens.* 2013;31(7):1281-357.

56. American Diabetes Association. Diagnosis and classification of diabetes mellitus. *Diabetes Care.* 2010 Jan;33 Suppl 1:S62-9.

57. Löllgen H, Böckenhoff A, Knapp G. Physical activity and all-cause mortality: an updated meta-analysis with different intensity categories. *Int J Sports Med.* 2009;30(3):213-24.

58. Grimnes G, Almaas B, Eggen AE, et al. Effect of smoking on the serum levels of 25-hydroxyvitamin D depends on the assay employed. *Eur J Endocrinol.* 2010 Aug;163(2):339-48.

59. Jorde R, Figenschau Y, Hutchinson M, Emaus N, Grimnes G. High serum 25-hydroxyvitamin D concentrations are associated with a favourable serum lipid profile. *Eur J Clin Nutr* 2010;64(12):1457-64.

60. Jorde R, Bonaa KH, Sundsfjord J. Population based study on serum ionised calcium, serum parathyroid hormone, and blood pressure. The Tromsø study. *Eur J Endocrinol.* 1999;141(4):350-7.

61. WORLD HEALTH ORGANIZATION: Diabetes mellitus: report of a WHO study group. Technical report series 727. WHO, Geneva, Switzerland (1985).

62. WHO. Global recommendations on physical activity for health. 2010. http://whqlibdoc.who.int/publications/2010/9789241599979_eng.pdf. Accessed 14th September 2014.

63. Woodard T, Sigurdsson S, Gotal JD, et al. Segmental kidney volumes measured by dynamic contrast-enhanced magnetic resonance imaging and their association with CKD in older people. *Am J Kidney Dis.* 2015;65(1):41-8.

64. Eiriksdottir G, Aspelund T, Bjarnadottir K, et al. Apolipoprotein E genotype and statins affect CRP levels through independent and different mechanisms: AGES-Reykjavik Study. *Atherosclerosis.* 2006;186(1):222-4.

65. van Ballegooijen AJ, Visser M, Cotch MF, et al. Serum vitamin D and parathyroid hormone in relation to cardiac structure and function: the ICELAND-MI sub study of AGES-Reykjavik. *J Clin Endocrinol Metab.* 2013;98(6):2544-52.

66. Whitworth JA1; World Health Organization, International Society of Hypertension Writing Group. 2003 World Health Organization (WHO)/International Society of Hypertension (ISH) statement on management of hypertension. *J Hypertens*. 2003 Nov; 21(11):1983-1992

67. Maunsell Z, Wright DJ, Rainbow SJ. Routine isotopedilution liquid chromatography-tandem mass spectrometry assay for simultaneous measurement of the 25-hydroxy metabolites of vitamins D2 and D3. *Clinical Chemistry.* 2005;51,1683-1690.

68. Paprott R, Schaffrath Rosario A, Busch MA, et al. Association Between Hemoglobin A1c and All-Cause Mortality: Results of the Mortality Follow-up of the German National Health Interview and Examination Survey 1998. *Diabetes Care.* 2015;38:249-256.

69. Mensink GB. [Physical activity]. *Gesundheitswesen.* 1999;61:S126-31. German.

70. Hintzpeter B, Mensink GB, Thierfelder W, Müller MJ, Scheidt-Nave C. Vitamin D status and health correlates among German adults. *Eur J Clin Nutr.* 2008;62(9):1079-89.

71. Schienkiewitz A, Mensink GB, Scheidt-Nave C. Comorbidity of overweight and obesity in a nationally representative sample of German adults aged 18-79 years. *BMC Public Health.* 2012;12:658.

72. Neuhauser HK, Adler C, Rosario AS, Diederichs C, Ellert U. Hypertension prevalence, awareness, treatment and control in Germany 1998 and 2008-11. *J Hum Hypertens.* 2015;29(4):247-53.

73. Burger M, Mensink G, Brönstrup A, Thierfelder W, Pietrzik K. Alcohol consumption and its relation to cardiovascular risk factors in Germany. *Eur J Clin Nutr.* 2004 Apr; 58(4):605-14.60.

74. Stel VS, Smit JH, Pluijm SM, Visser M, Deeg DJ, Lips P. Comparison of the LASA Physical Activity Questionnaire with a 7-day diary and pedometer. *J Clin Epidemiol.* 2004;57:252-8.

75. Puts MT, Visser M, Twisk JW, Deeg DJ, Lips P. Endocrine and inflammatory markers as predictors of frailty. *Clin Endocrinol (Oxf).* 2005;63(4):403-11.

76. Sohl E, de Jongh RT, Swart KM et al. The Association Between Vitamin D Status and Parameters for Bone Density and Quality is Modified by Body Mass Index. *Calcif Tissue Int.* 2015;96(2):113-22.

77. Kuchuk NO, Pluijm SM, van Schoor NM, Looman CW, Smit JH, Lips P. Relationships of serum 25-hydroxyvitamin D to bone mineral density and serum parathyroid hormone and markers of bone turnover in older persons. *J Clin Endocrinol Metab.* 2009;94(4):1244-50.

78. Herdzik E, Safranow K, Ciechanowski K: Diagnostic value of fasting capillary glucose, fructosamine and glycosylated haemoglobin in detecting and other glucose tolerance abnormalities compared to oral glucose tolerance test. *Acta Diabetol.* 2002;39:15-22.

79. Cashman KD, Kinsella M, McNulty BA, et al. Dietary vitamin D2-a potentially underestimated contributor to vitamin D nutritional status of adults? *Br J Nutr.* 2014;112(2):193-202.

80. Sempos CT, Vesper HW, Phinney KW, Thienpont LM, Coates PM. Vitamin D status as an international issue: National surveys and the problem of standardization. *Scand J Clin Lab Invest.* 2012;72:32-40.

81. Rahmani YE, Botelho JC & Vesper HW (2013). CDC Vitamin D Standardization Certification Program. Endocr Rev 34, (03_MeetingAbstracts): SUN-277.

82. Tian L, Durazo-Arvizu RA, Myers G, Brooks S, Sarafin K, Sempos CT. The estimation of calibration equations for variables with heteroscedastic measurement errors. *Stat Med.* 2014;33:4420-36.

83. Stewart GB, Altman DG, Askie LM, Duley L, Simmonds MC, Stewart LA. Statistical analysis of individual participant data meta-analyses: a comparison of methods and recommendations for practice. *PLoS One.* 2012;7(10):e46042.

84. Anderson KM. A nonproportional hazards Weibull accelerated failure time regression model. *Biometrics.* 1991;47(1):281-8.

85. Donnan PT, Donnelly L, New J, Morris AM: Predicting the absolute risk of major coronary heart disease (CHD) events in type 2 diabetes in the United Kingdom population. *Diabetes Care.* 2006,29(6):1231-6.

86. Collett, D. Modelling Survival Data in Medical Research, 2nd edition (2003), Chapman & Hall / CRC, Boca Raton, FL.

87. Stedman MR, Lew RA, Losina E, Gagnon DR, Solomon DH, Brookhart MA. A comparison of statistical approaches for physician-randomized trials with survival outcomes. *Contemp Clin Trials.* 2012;33(1):104-15.

88. SAS Institute Inc. 2008. SAS/STAT® 9.2 User’s Guide. The NLMIXED Procedure. Cary, NC: SAS Institute Inc.

89. Gagnier JJ, Moher D, Boon H, Beyene J, Bombardier C. Investigating clinical heterogeneity in systematic reviews: a methodologic review of guidance in the literature. *BMC Med Res Methodol.* 2012;12:111.

90. Klein JP, van Houwelingen HC, Ibrahim JG, Scheike TH. Handbook of Survival Analysis. Chapman & Hall/CRC Handbooks of Modern Statistical Methods. Chapman and Hall/CRC.

91. Rodrıguez, G. and Elo, I. Intra-class correlation in random-effects models for binary data. *The Stata Journal.* 2003;3(1):32-46.

92. Tierney JF, Vale C, Riley R, Smith CT, Stewart L, Clarke M, Rovers M. Individual Participant Data (IPD) Meta-analyses of Randomised Controlled Trials: Guidance on Their Use. *PLoS Med.* 2015;12(7):e1001855.

93. Ahmed I, Sutton AJ, Riley RD. Assessment of publication bias, selection bias, and unavailable data in meta-analyses using individual participant data: a database survey. *BMJ*. 2012;344:d7762.

94. John Amrhein. Introduction to Frailty Models. *McDougall Scientific Ltd. Paper.* 1492-2014.

95. Xian Liu. Survival Analysis: Models and Applications. Wiley 1 edition (August 13, 2012).

96. Stewart LA, Clarke M, Rovers M, Riley RD, Simmonds M, Stewart G, Tierney JF; PRISMA-IPD Development Group. Preferred Reporting Items for Systematic Review and Meta-Analyses of individual participant data: the PRISMA-IPD Statement. *JAMA*. 2015 Apr 28;313(16):1657-65.

**16. Supplementary Supplementary Figures**

# Supplementary Figure B. Dose-Response Trend of Hazard Ratios of Death From All Causes (95% CI) by Standardized Versus Original 25-Hydroxyvitamin D Concentrations in nmol/L excluding the New Hoorn study.

#
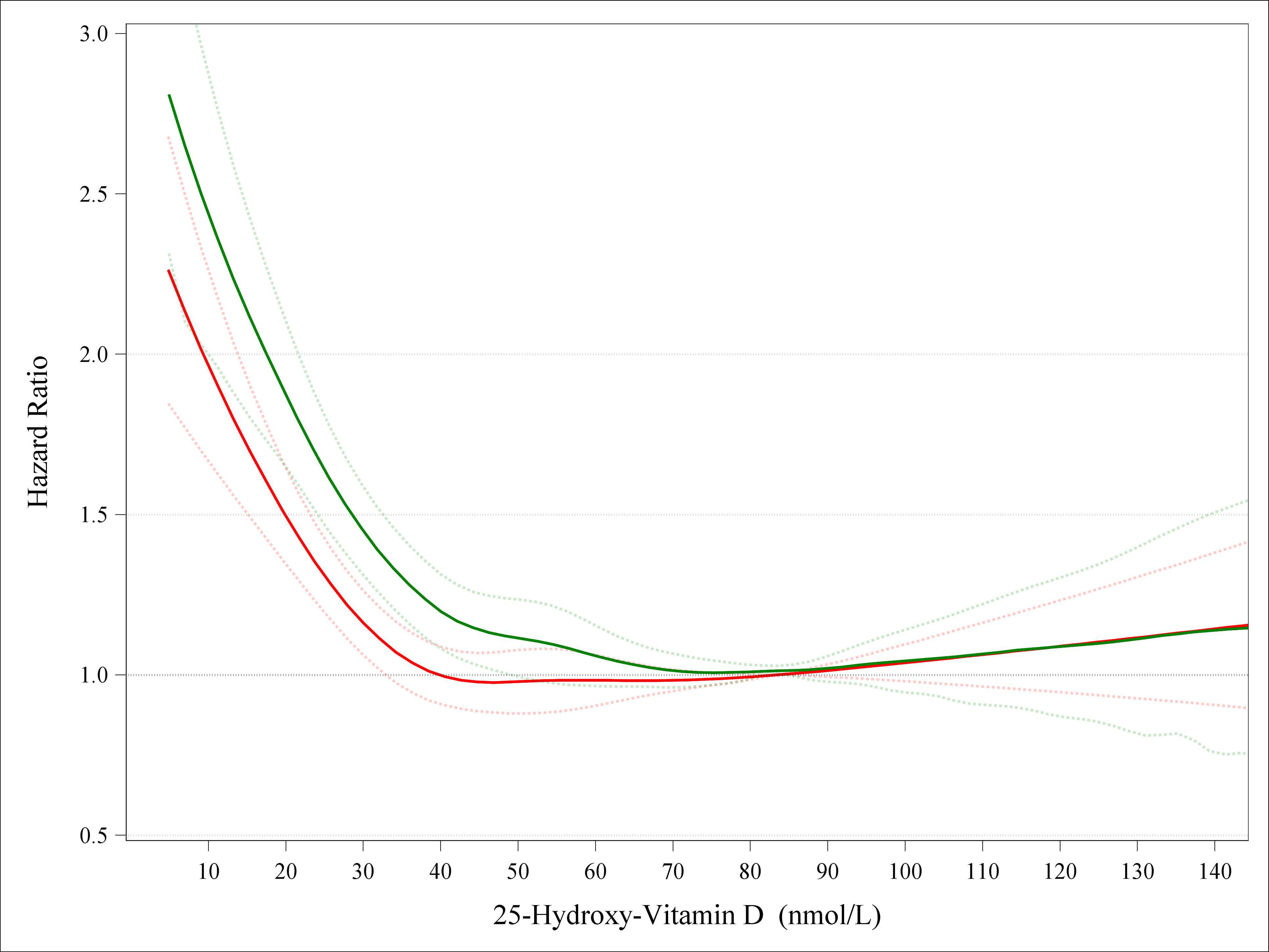


# Dose-response trend of hazard ratios for all-cause mortality adjusted for age, sex, season of blood drawing, and body mass index. Hazard ratios are shown for original 25-hydroxyvitamin D (red line with 95% CI as the dotted red lines) and for standardized 25-hydroxyvitamin D (green line with 95% CI as the dotted green lines) both excluding the New Hoorn study as the New Hoorn study had no original 25-hydroxyvitamin D values. Hazard ratios are referring to the standardized 25-hydroxyvitamin D concentration of 83.4 nmol/L (i.e. the median 25-hydroxyvitamin D concentration for the group with 25-hydroxyvitamin D concentrations from 75 to 99.99 nmol/L).

# Supplementary Figure C. Dose-Response Trend of Hazard Ratios of Death From All Causes (95% CI) by Standardized Versus Original 25-Hydroxyvitamin D Concentrations in nmol/L for the Tromsø Study.

#
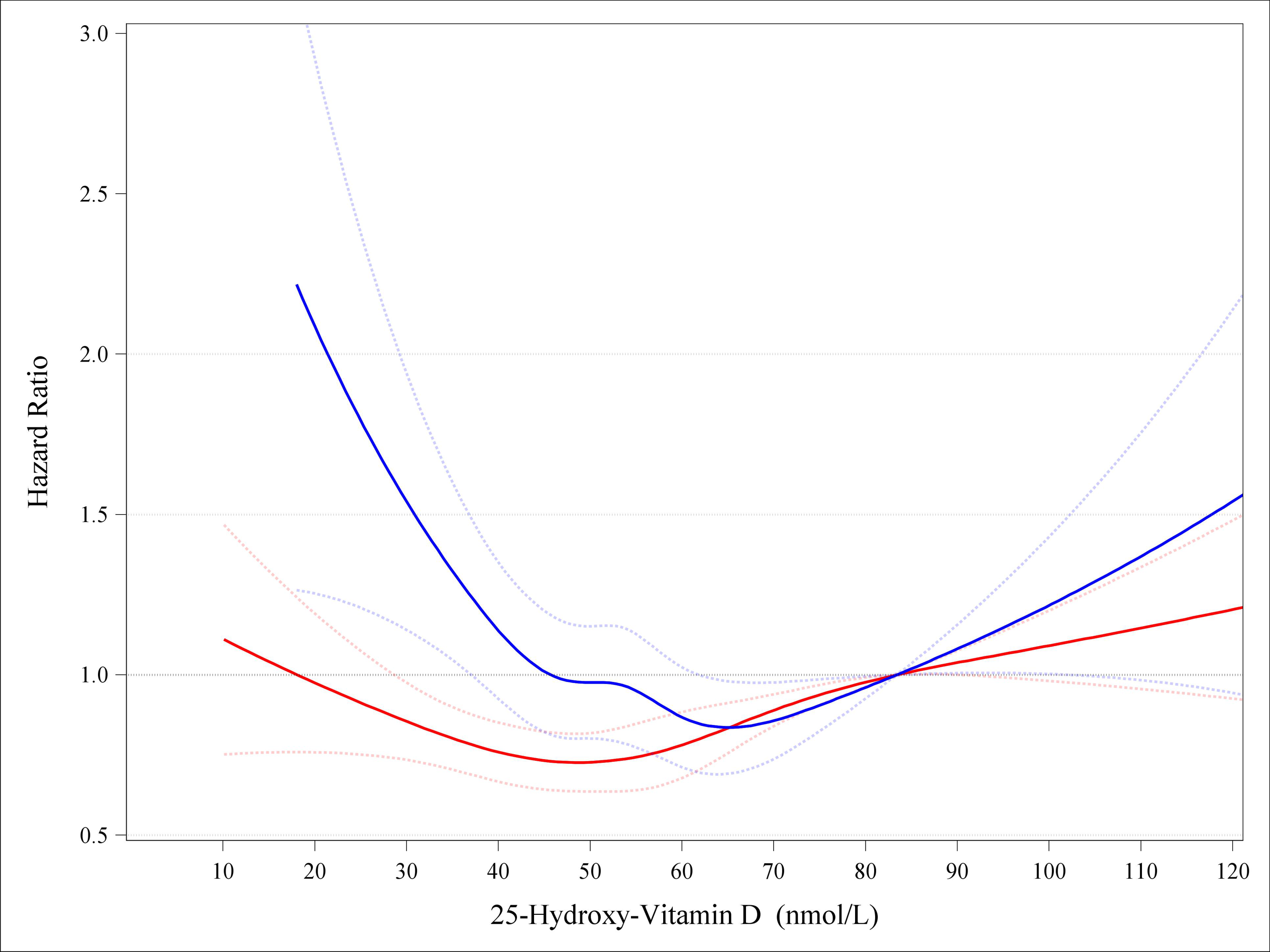


# Dose-response trend of hazard ratios for all-cause mortality adjusted for age, sex, season of blood drawing, and body mass index. Hazard ratios are shown for original 25-hydroxyvitamin D (red line with 95% CI as the dotted red lines) and for standardized 25-hydroxyvitamin D (blue line with 95% CI as the dotted blue lines). Hazard ratios are referring to the standardized 25-hydroxyvitamin D concentration of 83.4 nmol/L (i.e. the median 25-hydroxyvitamin D concentration for the group with 25-hydroxyvitamin D concentrations from 75 to 99.99 nmol/L).

# Supplementary Figure D. Dose-Response Trend of Hazard Ratios of Death From All Causes (95% CI) by Standardized Versus Original 25-Hydroxyvitamin D Concentrations in nmol/L for the Ludwigshafen RIsk and Cardiovascular Health Study.

#
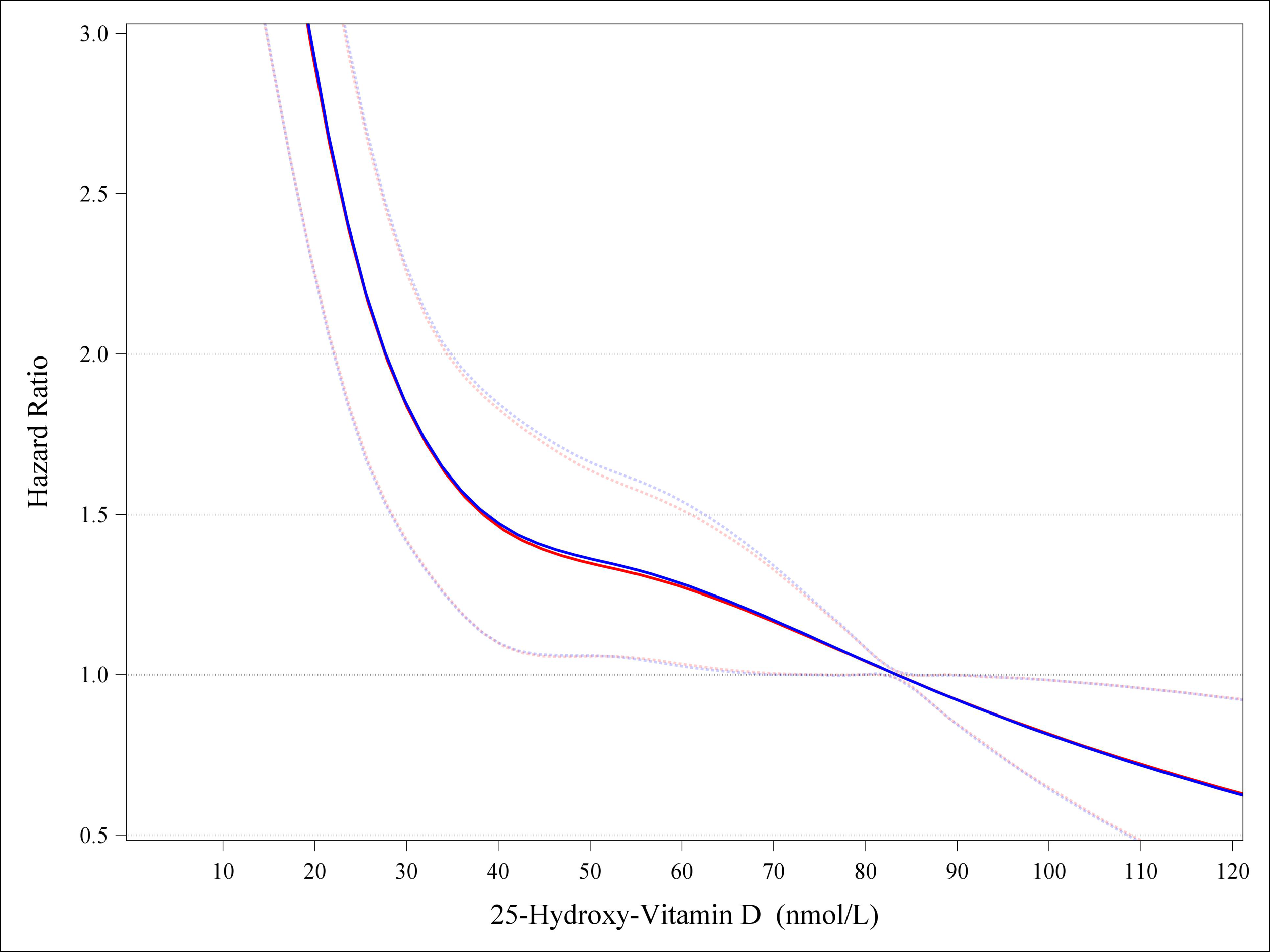


# Dose-response trend of hazard ratios for all-cause mortality adjusted for age, sex, season of blood drawing, and body mass index. Hazard ratios are shown for original 25-hydroxyvitamin D (red line with 95% CI as the dotted red lines) and for standardized 25-hydroxyvitamin D (blue line with 95% CI as the dotted blue lines). Hazard ratios are referring to the standardized 25-hydroxyvitamin D concentration of 83.4 nmol/L (i.e. the median 25-hydroxyvitamin D concentration for the group with 25-hydroxyvitamin D concentrations from 75 to 99.99 nmol/L).

**Supplementary Figure E.** Dose-Response Trend of Hazard Ratios of Death From All Causes (95% CI) by Standardized Versus Original 25-Hydroxyvitamin D Concentrations in nmol/L for the Age, Gene/Environment Susceptibility Reykjavik Study.

#
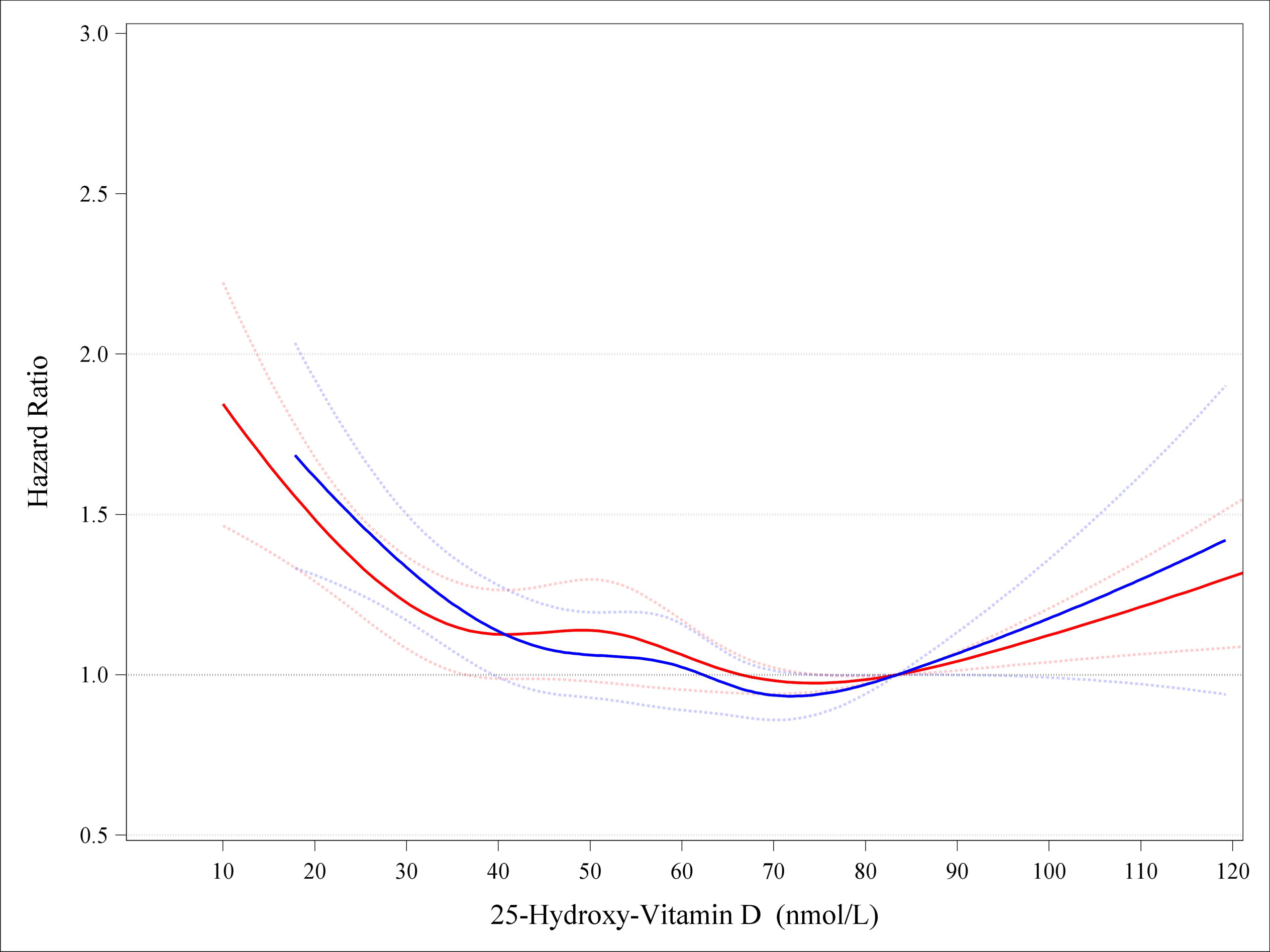


Dose-response trend of hazard ratios for all-cause mortality adjusted for age, sex, season of blood drawing, and body mass index. Hazard ratios are shown for original 25-hydroxyvitamin D (red line with 95% CI as the dotted red lines) and for standardized 25-hydroxyvitamin D (blue line with 95% CI as the dotted blue lines). Hazard ratios are referring to the standardized 25-hydroxyvitamin D concentration of 83.4 nmol/L (i.e. the median 25-hydroxyvitamin D concentration for the group with 25-hydroxyvitamin D concentrations from 75 to 99.99 nmol/L).

# Supplementary Figure F. Dose-Response Trend of Hazard Ratios of Death From All Causes (95% CI) by Standardized Versus Original 25-Hydroxyvitamin D Concentrations in nmol/L for the German Health Interview and Examination Survey for Adults.

#
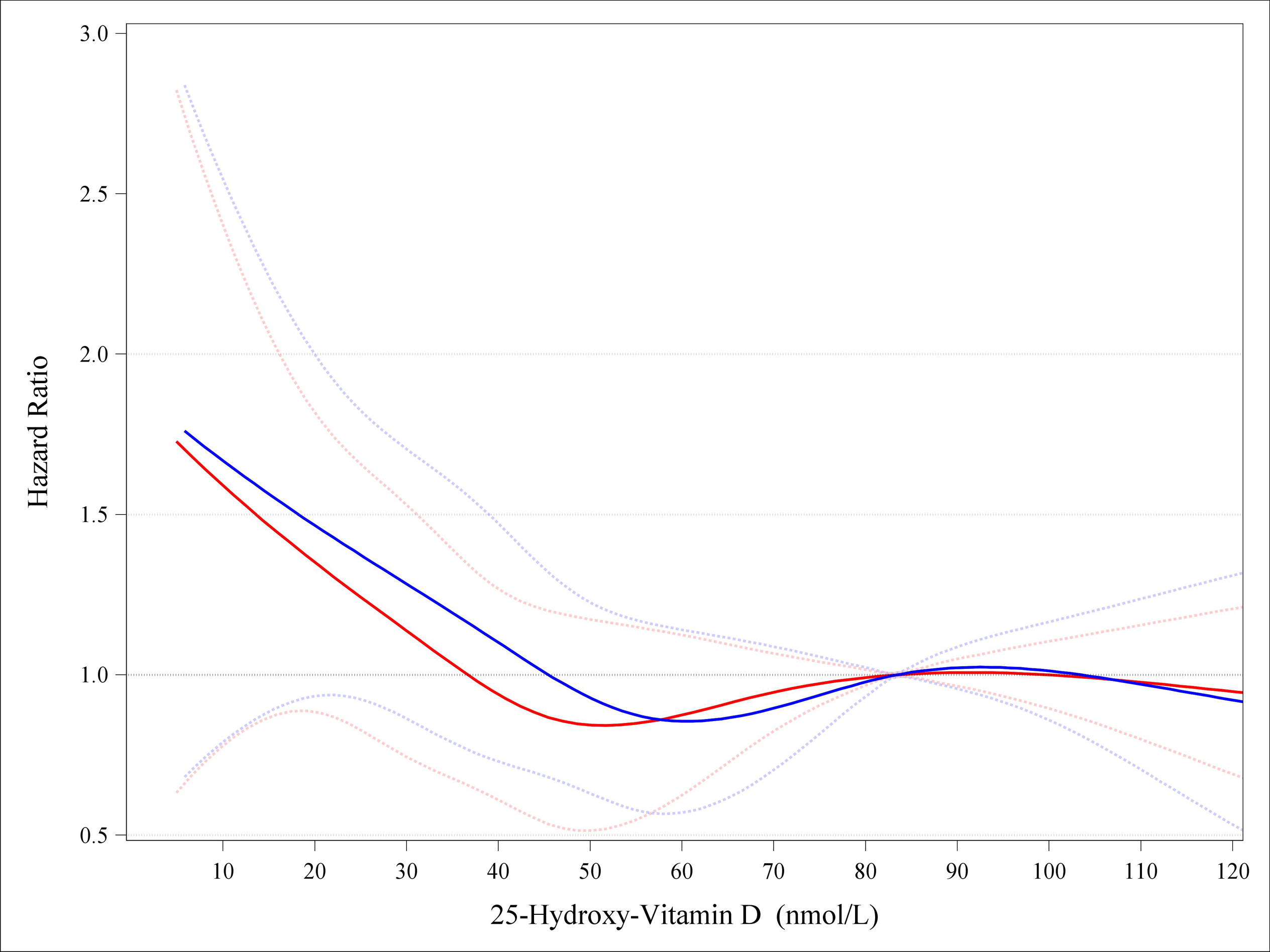


Dose-response trend of hazard ratios for all-cause mortality adjusted for age, sex, season of blood drawing, and body mass index. Hazard ratios are shown for original 25-hydroxyvitamin D (red line with 95% CI as the dotted red lines) and for standardized 25-hydroxyvitamin D (blue line with 95% CI as the dotted blue lines). Hazard ratios are referring to the standardized 25-hydroxyvitamin D concentration of 83.4 nmol/L (i.e. the median 25-hydroxyvitamin D concentration for the group with 25-hydroxyvitamin D concentrations from 75 to 99.99 nmol/L).

# Supplementary Figure G. Dose-Response Trend of Hazard Ratios of Death From All Causes (95% CI) by Standardized Versus Original 25-Hydroxyvitamin D Concentrations in nmol/L for the Longitudinal Aging Study Amsterdam, first cohort.

#
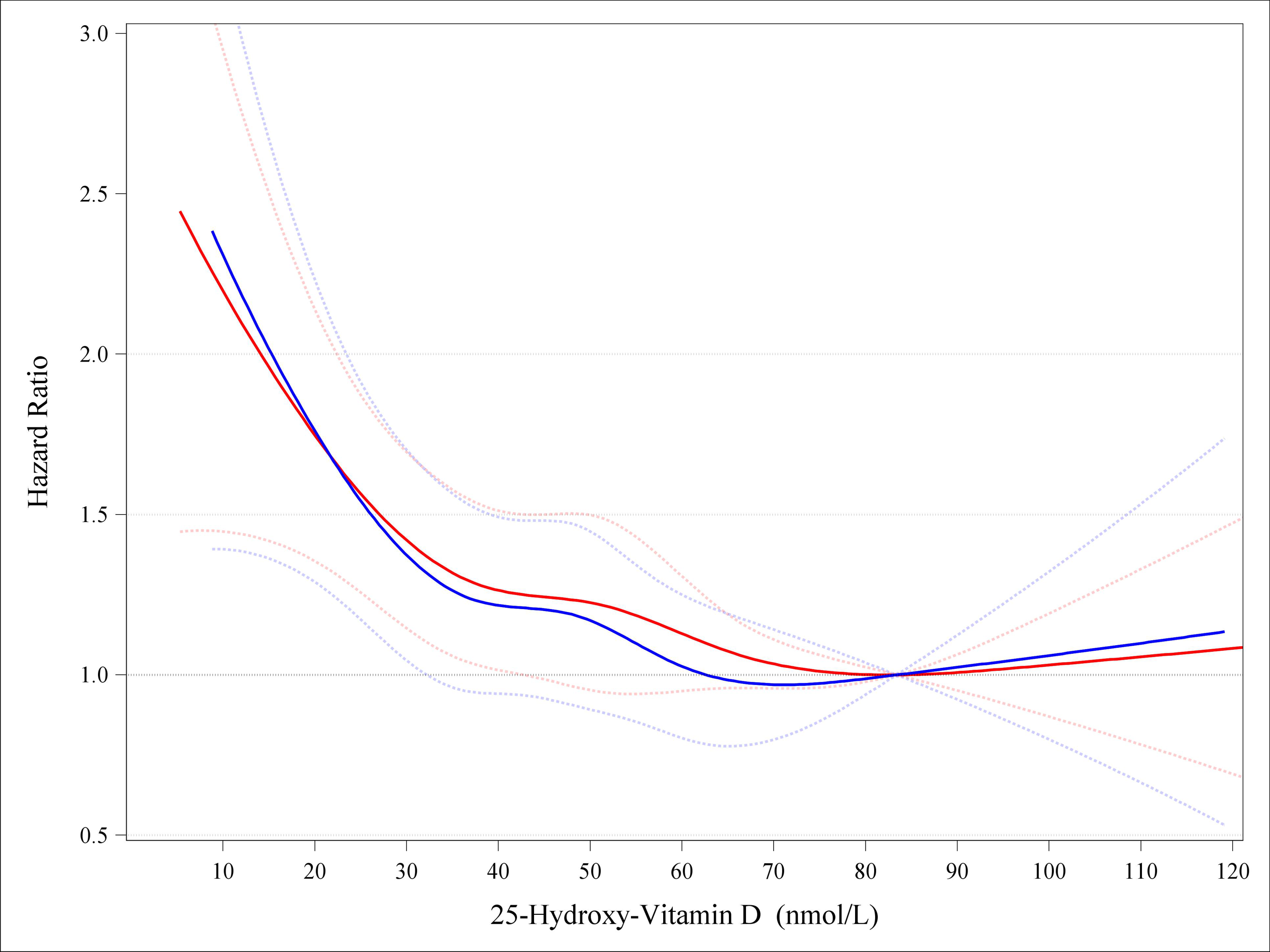


Dose-response trend of hazard ratios for all-cause mortality adjusted for age, sex, season of blood drawing, and body mass index. Hazard ratios are shown for original 25-hydroxyvitamin D (red line with 95% CI as the dotted red lines) and for standardized 25-hydroxyvitamin D (blue line with 95% CI as the dotted blue lines). Hazard ratios are referring to the standardized 25-hydroxyvitamin D concentration of 83.4 nmol/L (i.e. the median 25-hydroxyvitamin D concentration for the group with 25-hydroxyvitamin D concentrations from 75 to 99.99 nmol/L).

For Aarhus Aarhus Mammography Cohort Study, New Hoorn Study and Longitudinal Aging Study Amsterdam, second cohort, the total number of deaths was considered to be too small for graphical implementation.

**17. Supplementary Supplementary Tables**

**Supplementary Table A.** Description of Cohort Studies included in Meta-Analyses of the Associations between Serum 25-Hydroxyvitamin D Concentrations and Mortality Outcomes.

| Study name | Place and years of baseline examination | Study sample description | 25(OH)D assay | Study population total, N | With measured Total 25(OH)D^†^, N | Included in Analysis^‡^, N | Excluded^‡^, % |
| --- | --- | --- | --- | --- | --- | --- | --- |
| Overall | — | — |  | 47856 | 27133 | 26916 | 43.8 |
| Tromsø | Tromsø, north Norway, 1994-95^§^ | Population-based | Modular E170 Roche^a^ | 26956 | 7160 | 7145 | 73.5 |
| LURIC | Ludwigshafen, Germany, 1997-2000 | Hospital-based^¶^ | RIA Diasorin^b^ | 3316 | 3299 | 3299 | 0.5 |
| AGES | Reykjavík, Iceland, 2002-06 | Population-based | CLIA Diasorin^c^ | 5764 | 5519 | 5510 | 4.4 |
| NHS | City of Hoorn, the Netherlands, 2006/07 | Population-based | LC-MS^#^ | 2807 | 2625 | 2591 | 7.7 |
| Aarhus | County of Aarhus, Denmark, 2003-07 | Population-based | LC-MS^d^ | 2555 | 2555 | 2473 | 3.2 |
| DEGS^‖^ | Nation-wide, Germany, 1997-99 | Population-based | CLIA Diasorin^c^ | 4030 | 3917 | 3862 | 4.2 |
| LASA, first cohort | Regions Amsterdam, Zwolle and Oss, the Netherlands, 1995/96 | Population-based, older cohort | RIA^e^ | 1509 | 1320 | 1302 | 13.7 |
| LASA, second cohort | Similar to LASA 1, 2002/03 | Population-based, younger cohort | CLIA Diasorin^c^ | 919 | 738 | 734 | 20.1 |

Characteristics are presented as total number (N) or percentage (%) where appropriate. Abbreviations: Tromsø = Tromsø Study; LURIC = Ludwigshafen RIsk and Cardiovascular Health Study; AGES = Age, Gene/Environment Susceptibility Reykjavik Study; NHS = New Hoorn Study; Aarhus = Aarhus Mammography Cohort Study; DEGS = German Health Interview and Examination Survey for Adults; LASA = Longitudinal Aging Study Amsterdam; 25(OH)D=25-hydroxyvitamin D; CLIA = ChemiLuminescent ImmunoAssay; LC-MS = Liquid chromatography-mass spectrometry; RIA = radioimmunoassay; ^*^Manufacturers of 25(OH)D assays originally used in the single cohort studies: ^a^ECLIA on a Modular E170 Roche (Roche Diagnostics, Rotkreuz, Swizerland), ^b^RIA (DiaSorin Inc, Antony, France, and Stillwater, MN), ^c^CLIA Diasorin-Liaison (Diasorin, Stillwater, USA), ^d^Aarhus hospital in-house isotope dilution liquid chromatography-tandem mass spectrometry (not commercially available); ^e^RIA (Nichols Diagnostics Capistrano, CA, USA). **^†^**Number of participants with available measurements of standardized 25(OH)D. **^‡^**Inclusion criterion for the present analysis were available participant data for original and standardized 25(OH)D measurements, data on vital status at follow-up and follow-up time/censoring time, data on age, sex, body mass index and season of blood sampling. ^§^Baseline visit for the Tromsø Study was the 4^th^ cycle. ^¶^Study participants were referred to diagnostic angiography. ^#^NHS had no original 25(OH)D and was measured in full by the same LC-MS method used for the standardization of the other studies. ^‖^ For the present analysis, the German National Health Interview and Examination Survey 1998 (GNHIES98) with the integrated German Nutrition Survey 1998 (GeNuS98) was the baseline visit.

**Supplementary Table B.** Comparison of Original and Standardized 25-hydroxyvitamin D Values, both in nmol/L.

| Study | Original 25(OH)D^a^, nmol/L | Percentage <30nmol/L^b^, % | Percentage <40nmol/L^b^, % | Percentage <50nmol/L^b^, % | Standardized 25(OH)D, nmol/L | Percentage <30nmol/L^c^, % | Percentage <40nmol/L^c^, % | Percentage <50nmol/L^c^, % | Mean difference ±SE^d^, nmol/L |
| --- | --- | --- | --- | --- | --- | --- | --- | --- | --- |
| Overall (N=26916)^e^ | 52.5 (37.1-69.2) | 13.9 | 26.6 | 41.4 | 53.8^f^ (41.4-66.1) | 11.0 ^e^ | 22.3 ^e^ | 42.0 ^e^ | 0.1838  ±0.0542 ^*, e^ |
| Tromsø (N=7145) | 57.0 (44.8-70.4) | 4.9 | 16.5 | 35.4 | 53.5 (46.5-61.2) | 0.7 | 8.7 | 36.7 | -4.4704  ±0.1394 ^*^ |
| LURIC (N=3299) | 39.0 (25.3-57.5) | 33.6 | 51.7 | 65.9 | 38.6 (25.0-56.8) | 34.0 | 52.5 | 66.5 | -0.5333  ±0.0070 ^*^ |
| AGES (N=5510) | 51.8 (35.8-67.6) | 17.3 | 31.3 | 47.0 | 59.1 (44.0-68.8) | 8.4 | 19.6 | 33.6 | 3.7098  ±0.0998 ^*^ |
| NHS (N=2591) | N.A. | N.A. | N.A. | N.A. | 59.1 (44.7-73.1) | 9.1 | 18.8 | 33.5 | N.A. |
| Aarhus (N=2473) | 61.9 (45.6-78.3) | 8.5 | 18.2 | 31.0 | 58.0 (43.0-72.0) | 10.1 | 21.5 | 36.7 | -4.5252  ±0.0588 ^*^ |
| DEGS (N=3862) | 46.0 (32.2-71.0) | 21.7 | 40.0 | 56.1 | 55.9 (39.0-86.4) | 14.3 | 26.3 | 42.1 | 6.8579  ±0.1331 ^*^ |
| LASA, first cohort (N=1302) | 51.0 (36.2-68.2) | 17.5 | 31.1 | 48.2 | 48.5 (35.6-61.9) | 17.2 | 33.0 | 52.9 | -4.1741  ±0.1557 ^*^ |
| LASA, second cohort (N=734) | 55.0 (42.0-68.1) | 7.6 | 20.7 | 40.5 | 54.3 (41.7-66.9) | 7.6 | 21.4 | 41.1 | -1.4601 ±0.0882 ^*^ |

Comparison of original and standardized 25-hydroxyvitamin-D values with mean difference and standard errors (SE) were calculated by Student’s paired t-test. Characteristics are presented as median with interquartile range (IQR), mean difference with SE or percentage (%) where appropriate. Abbreviations: 25(OH)D = 25-hydroxyvitamin D; Tromsø = Tromsø Study; LURIC = Ludwigshafen RIsk and Cardiovascular Health Study; AGES = Age, Gene/Environment Susceptibility Reykjavik Study; NHS = New Hoorn Study; Aarhus = Aarhus Mammography Cohort Study; DEGS = German Health Interview and Examination Survey for Adults; LASA = Longitudinal Aging Study Amsterdam; N.A. = Not available; *Statistically significant (P<0.05). ^a^Original 25(OH)D values were measured by study-specific assays. ^b^Percentage of individuals with original 25(OH)D level below given threshold. ^c^Percentage of individuals with standardized 25(OH)D level below given threshold. ^d^Mean absolute difference was defined as standardized 25(OH)D measurements minus original measurements. ^e^The overall number, percentages and mean difference ±SE consisted of individual participants from Tromsø, LURIC, AGES, Aarhus, LASA, DEGS, first and second cohort, as NHS had no original 25(OH)D measurements. ^f^The overall median 25(OH)D value for all studies (including NHS; n=26 916) was 55.4 nmol/L (41.7-66.8). For all studies, the percentage of individuals with standardized 25(OH)D <30, <40, and <50 nmol/L was 10.96, 22.5, and 41.15 %, respectively. For all studies from the general population (i.e. all studies except LURIC; n=23 617), the percentage of individuals with original measurements of 25(OH)D <30, <40, and <50 nmol/L was 7.7, 18.3, and 37.6%, respectively.

**Supplementary Table C.** Standardized 25-hydroxyvitamin D concentrations by baseline characteristics of the study populations, in nmol/L.

| Characteristic | Total cohort | Tromsø | LURIC | AGES | NHS | Aarhus Mammography | DEGS | LASA, first cohort | LASA, second cohort |
| --- | --- | --- | --- | --- | --- | --- | --- | --- | --- |
| Age, in years |  |  |  |  |  |  |  |  |  |
| <60 | 55.0 (42.7-69.1)^*^ | 54.8 (47.7-62.7)^*^ | 42.5 (28.0-60.0)^*^ | N.A. | 58.1 (43.5-72.2)^*^ | 57.0 (42.0-73.0)^*^ | 56.9 (39.5-88.2)^*^ | N.A. | 53.9 (42.7-68.0) |
| 60-69.9 | 53.4 (42.4-65.0)^*^ | 52.8 (46.0-60.3)^*^ | 41.0 (26.5-58.0)^*^ | 58.3 (43.0-67.7) | 62.7 (48.3-75.9)^*^ | 61.0 (48.0-73.0)^*^ | 56.3 (40.8-84.4)^*^ | 58.5 (43.1-68.5)^*^ | 54.7 (41.1-66.9) |
| ≥70 | 53.7 (39.0-65.5)^*^ | 51.4 (44.7-58.7)^*^ | 32.4 (20.9-46.4)^*^ | 59.2 (44.0-68.9) | N.A. | 59.0 (48.0-66.8)^*^ | 45.0 (30.0-67.4)^*^ | 45.5 (32.4-58.9)^*^ | N.A. |
| Sex |  |  |  |  |  |  |  |  |  |
| Women | 54.4 (42.0-67.0)^*^ | 53.5 (46.1-61.2) | 32.4 (20.9-52.8)^*^ | 57.6 (42.2-67.2)^*^ | 60.0 (46.0-75.6)^*^ | 58.0 (43.0-72.0) | 55.8 (39.4-89.2)^*^ | 44.3 (32.3-58.3)^*^ | 53.9 (42.3-69.1) |
| Men | 53.9 (41.0-66.6)^*^ | 53.5 (47.1-61.1) | 41.0 (27.7-58.8)^*^ | 61.1 (46.9-71.1)^*^ | 57.7 (43.1-70.2)^*^ | N.A. | 56.0 (38.5-82.6)^*^ | 53.4 (40.0-65.3)^*^ | 54.8 (41.4-65.5) |
| Season^a^ |  |  |  |  |  |  |  |  |  |
| Winter | 50.4 (38.9-61.9)^*^ | 51.5 (44.3-59.0)^*^ | 31.6 (20.9-45.2)^*^ | 57.8 (41.7-67.5)^*^ | 49.1 (36.5-65.3)^*^ | 51.0 (39.0-67.0)^*^ | 47.0 (36.6-69.9)^*^ | 44.6 (30.1-58.7)^*^ | 54.0 (42.4-67.3)^*^ |
| Spring | 53.9 (40.0-65.7)^*^ | 56.9 (49.7-46.6)^*^ | 28.2 (19.4-40.3)^*^ | 60.5 (44.3-71.1)^*^ | 67.1 (55.1-81.7)^*^ | 53.0 (39.0-68.0)^*^ | 46.0 (30.0-71.0)^*^ | 43.7 (33.9-59.0)^*^ | 53.4 (40.8-65.5)^*^ |
| Summer | 61.2 (47.0-75.6)^*^ | 62.3 (54.0-69.9)^*^ | 51.1 (35.6-66.2)^*^ | 61.2 (47.8-71.0)^*^ | 63.5 (49.8-76.3)^*^ | 67.5 (55.0-83.0)^*^ | 63.1 (43.8-92.2)^*^ | 53.9 (40.9-66.2)^*^ | 62.0 (48.2-72.7)^*^ |
| Autumn | 54.7 (43.4-67.3)^*^ | 51.2 (45.1-58.2)^*^ | 44.5 (30.2-60.2)^*^ | 58.4 (43.8-67.2)^*^ | 50.3 (36.2-62.6)^*^ | 60.0 (47.0-74.0)^*^ | 47.0 (36.6-69.9)^*^ | 53.0 (40.1-62.8)^*^ | 46.5 (38.7-52.6)^*^ |
| BMI^b^ |  |  |  |  |  |  |  |  |  |
| Normal or  Underweight | 56.9 (44.1-69.5)^*^ | 54.5 (47.5-62.8)^*^ | 37.0 (23.8-57.0)^*^ | 61.4 (45.9-70.5)^*^ | 62.8 (48.7-76.7)^*^ | 60.0 (46.0-74.0)^*^ | 59.9 (40.8-91.8)^*^ | 51.2 (36.8-65.4)^*^ | 56.1 (42.4-66.9) |
| Overweight | 54.2 (41.8-66.3)^*^ | 53.7 (46.8-60.9)^*^ | 41.0 (26.8-58.2)^*^ | 59.5 (45.7-69.0)^*^ | 58.2 (43.4-71.9)^*^ | 57.0 (41.0-70.0)^*^ | 56.0 (39.0-87.6)^*^ | 48.7 (36.6-61.3)^*^ | 55.6 (42.9-69.0) |
| Obesity | 48.6 (36.7-61.2)^*^ | 49.6 (43.4-57.2)^*^ | 36.1 (23.8-52.1)^*^ | 53.0 (39.2-64.8)^*^ | 51.9 (37.4-64.5)^*^ | 47.0 (35.0-61.0)^*^ | 49.1 (36.0-69.8)^*^ | 43.4 (32.1-55.6)^*^ | 50.9 (40.0-64.5) |
| Smoking |  |  |  |  |  |  |  |  |  |
| No current smoking | 55.0 (42.1-67.1)^*^ | 53.9 (46.8-61.6)^*^ | 39.3 (25.8-56.5) | 59.7 (45.3-69.1)^*^ | 59.9 (46.1-74.0)^*^ | 58.0 (45.0-73.0)^*^ | 55.8 (39.2-84.5) | 48.7 (35.9-62.1) | 56.5 (44.0-69.6)^*^ |
| Current | 52.0 (40.4-65.0)^*^ | 52.6 (45.6-60.5)^*^ | 36.0 (22.8-57.0) | 52.9 (36.4-66.0)^*^ | 55.2 (40.1-71.3)^*^ | 55.0 (38.0-72.0)^*^ | 55.8 (38.1-89.6) | 45.7 (33.9-61.5) | 47.7 (38.1-61.5)^*^ |
| Characteristic | Total cohort | Tromsø | LURIC | AGES | NHS | Aarhus Mammography | DEGS | LASA, first cohort | LASA, second cohort |
| Physical activity^c^ |  |  |  |  |  |  |  |  |  |
| Low frequency | 51.3 (38.8-63.4)^*^ | 52.6 (45.7-60.3)^*^ | 35.9 (23.3-53.1)^*^ | 58.0 (41.7-67.9)^*^ | 56.3 (39.6-69.0)^*^ | N.A. | 53.6 (36.6-80.6)^*^ | 44.2 (32.2-59.1)^*^ | 51.6 (40.7-66.9) |
| Moderate frequency | 57.0 (45.1-68.9)^*^ | 55.3 (48.1-62.8)^*^ | 47.9 (33.2-63.8)^*^ | 61.0 (47.6-70.0)^*^ | 59.3 (44.8-74.1)^*^ | N.A. | 59.4 (42.1-91.2)^*^ | 53.8 (41.2-65.7)^*^ | 55.2 (42.4-67.5) |
| High frequency | 60.2 (47.8-73.1)^*^ | 55.0 (48.3-63.0)^*^ | 57.0 (40.4-74.2)^*^ | 62.7 (52.6-70.9)^*^ | 62.7 (50.1-75.7)^*^ | N.A. | 60.5 (42.0-90.8)^*^ | 56.9 (48.1-65.0)^*^ | 58.4 (47.6-68.3) |
| Present  diabetes^d^ |  |  |  |  |  |  |  |  |  |
| No | 55.0 (42.8-67.2)^*^ | 53.6 (46.7-61.4)^*^ | 41.3 (27.0-59.5)^*^ | 59.5 (44.5-69.0)^*^ | 60.4 (46.2-73.8)^*^ | 58.0 (44.0-72.3)^*^ | 56.6 (39.9-87.8)^*^ | 48.9 (36.1-62.3)^*^ | 54.9 (42.6-68.3)^*^ |
| Yes | 46.2 (31.2-61.9)^*^ | 49.9 (43.4-56.9)^*^ | 34.4 (21.7-49.4)^*^ | 55.7 (40.3-66.9)^*^ | 54.8 (40.4-71.1)^*^ | 49.0 (33.0-67.0)^*^ | 47.2 (31.3-68.7)^*^ | 41.2 (30.5-54.8)^*^ | 44.0 (35.0-57.6)^*^ |
| Glucose, in mmol/L |  |  |  |  |  |  |  |  |  |
| <7.0 | 55.7 (38.6-70.5)^*^ | N.A. | 39.5 (26.0-57.7)^*^ | 59.3 (44.2-68.9)^*^ | 60.5 (46.2-73.9)^*^ | N.A. | 56.3 (39.7-87.2)^*^ | 51.1 (39.2-67.4) | 59.4 (46.1-70.9) |
| ≥7.0 | 48.2 (32.2-64.5)^*^ | N.A. | 33.9 (20.9-48.1)^*^ | 57.1 (41.3-67.2)^*^ | 53.2 (39.4-69.8)^*^ | N.A. | 47.1 (32.4-70.5)^*^ | 52.5 (35.8-63.8) | 47.0 (40.3-61.5) |
| Present HTN^e^ |  |  |  |  |  |  |  |  |  |
| No | 56.9 (45.0-69.6)^*^ | 54.6 (47.5-62.3)^*^ | 46.8 (28.3-63.9)^*^ | 60.2 (47.1-69.0)^*^ | 59.9 (45.8-74.2)^*^ | 58.0 (44.0-72.0) | 59.0 (41.5-90.9)^*^ | 48.5 (36.7-61.9) | 56.6 (42.5-70.3)^*^ |
| Yes | 52.3 (39.4-65.0)^*^ | 52.7 (45.8-60.4)^*^ | 38.1 (24.8-56.0)^*^ | 59.0 (43.3-68.7)^*^ | 57.5 (43.1-71.8)^*^ | 57.0 (43.0-74.0) | 52.3 (36.4-78.3)^*^ | 48.6 (35.4-62.0) | 52.4 (41.5-66.2)^*^ |
| SBP, in mmHg |  |  |  |  |  |  |  |  |  |
| <140 | 55.2 (42.3-67.9)^*^ | 54.5 (47.5-62.2)^*^ | 39.5 (25.3-57.5)^*^ | 59.3 (44.4-68.9) | 59.5 (45.7-74.0)^*^ | N.A. | 57.8 (40.5-89.7)^*^ | 48.7 (36.3-62.2) | 54.3 (42.1-68.2) |
| ≥140 | 52.3 (40.6-64.4)^*^ | 52.5 (45.7-60.1)^*^ | 37.8 (24.8-56.0)^*^ | 59.0 (43.7-68.6) | 57.4 (42.5-71.8)^*^ | N.A. | 52.3 (35.6-78.2)^*^ | 48.7 (35.6-62.1) | 54.5 (41.7-66.7) |

| Characteristic | Total cohort | Tromsø | LURIC | AGES | NHS | Aarhus Mammography | DEGS | LASA, first cohort | LASA, second cohort |
| --- | --- | --- | --- | --- | --- | --- | --- | --- | --- |
| LDL, (in mmol/L |  |  |  |  |  |  |  |  |  |
| <2.6 | 53.6 (37.1-68.7) | 54.8 (46.4-63.6) | 37.3 (24.6-55.6) | 60.5 (46.1-72.1)^*^ | 57.0 (43.6-74.1) | N.A. | 61.5 (42.4-92.5)^*^ | 42.0 (27.9-55.3)^*^ | 55.9 (45.4-67.2) |
| 2.6-4.0 | 53.9 (40.5-67.2) | 53.4 (46.2-61.3) | 39.5 (25.5-57.0) | 59.1 (43.9-68.9)^*^ | 59.4 (45.5-72.4) | N.A. | 56.9 (39.8-88.4)^*^ | 48.2 (35.7-61.7)^*^ | 59.9 (45.1-72.4) |
| ≥4.1 | 54.1 (44.2-64.4) | 53.7 (46.9-61.2) | 37.8 (24.2-56.7) | 58.4 (42.7-67.4)^*^ | 59.3 (44.2-73.2) | N.A. | 53.9 (37.8-81.6)^*^ | 51.3 (38.6-64.5)^*^ | 55.8 (46.1-68.4) |
| History of CVD^f^ |  |  |  |  |  |  |  |  |  |
| No | 54.4 (42.5-66.6)^*^ | 53.5 (46.6-61.2) | 39.5 (26.0-57.0)^*^ | 59.3 (44.5-68.7) | N.A. | 58.0 (43.0-72.0)^*^ | 56.0 (39.2-86.4) | 48.6 (36.1-62.3)^*^ | 54.5 (41.7-66.9) |
| Yes | 48.6 (32.9-63.2)^*^ | 53.2 (46.1-60.3) | 37.3 (24.2-56.0)^*^ | 58.3 (42.2-69.0) | N.A. | 64.0 (42.0-81.5)^*^ | 48.1 (34.0-74.7) | 44.8 (30.0-58.5)^*^ | 52.5 (43.3-68.6) |
| History of  cancer |  |  |  |  |  |  |  |  |  |
| No | 53.8 (41.6-66.2)^*^ | 53.5 (46.6-61.2)^*^ | 39.1 (25.3-57.0) | 59.4 (44.4-69.1)^*^ | N.A. | 58.0 (44.0-73.0) | 56.0 (39.0-86.8) | 48.7 (36.1-62.3) | 54.3 (42.3-67.3) |
| Yes | 53.4 (39.3-64.6)^*^ | 52.8 (45.6-60.9)^*^ | 35.9 (21.7-52.4) | 58.1 (42.4-67.1)^*^ | N.A. | 58.0 (41.0-71.0) | 53.9 (35.0-82.7) | 46.1 (33.9-60.4) | 52.2 (38.6-66.7) |
| eGFR, in mL/min/1.73m² |  |  |  |  |  |  |  |  |  |
| <60 | 53.2 (37.3-66.8)^*^ | 55.2 (47.1-63.5) | 33.4 (20.4-50.3)^*^ | 58.3 (42.2-68.8)^*^ | N.A. | 62.0 (45.5-76.0) | 50.5 (36.4-73.1)^*^ | 47.3 (35.0-60.7) | 58.4 (45.3-70.3)^*^ |
| ≥60 | 53.9 (42.0-66.0)^*^ | 53.5 (46.5-61.2) | 39.5 (25.8-57.3)^*^ | 59.4 (44.8-68.8)^*^ | N.A. | 58.0 (43.0-72.0) | 56.1 (38.9-87.1)^*^ | 49.2 (36.0-62.7) | 53.4 (41.3-66.7)^*^ |
| CRP, in mg/L |  |  |  |  |  |  |  |  |  |
| <2 | 55.0 (38.3-69.7)^*^ | N.A. | 41.0 (27.7-58.5)^*^ | 59.6 (45.5-68.8)^*^ | N.A. | N.A. | 55.0 (38.7-83.4)^*^ | 51.4 (38.8-64.6)^*^ | N.A. |
| ≥2 | 51.0 (34.4-67.1)^*^ | N.A. | 37.1 (23.6-55.0)^*^ | 58.6 (42.2-68.8)^*^ | N.A. | N.A. | 57.3 (39.7-90.2)^*^ | 47.7 (34.9-60.5)^*^ | N.A. |

| Characteristic | Total cohort | Tromsø | LURIC | AGES | NHS | Aarhus Mammography | DEGS | LASA, first cohort | LASA, second cohort |
| --- | --- | --- | --- | --- | --- | --- | --- | --- | --- |
| Intake of calcium supplements |  |  |  |  |  |  |  |  |  |
| No | 52.6 (39.7-64.1)^*^ | 53.9 (46.8-61.5)^*^ | 38.6 (25.0-56.8)^*^ | 58.5 (42.3-68.3)^*^ | N.A. | 55.0 (40.0-70.0)^*^ | N.A. | 48.1 (34.7-61.3)^*^ | N.A. |
| Yes | 61.4 (50.5-71.0)^*^ | 57.3 (48.5-63.9)^*^ | 39.7 (30.7-63.3)^*^ | 62.5 (51.9-70.7)^*^ | N.A. | 65.0 (55.0-77.0)^*^ | N.A. | 55.7 (40.4-66.0)^*^ | N.A. |
| Intake of vitamin D supplements |  |  |  |  |  |  |  |  |  |
| No | 48.0 (35.3-59.5)^*^ | 52.2 (45.3-59.8)^*^ | 38.6 (25.0-56.5)^*^ | 41.1 (30.9-56.8) | N.A. | 52.0 (37.0-68.0)^*^ | N.A. | N.A. | N.A. |
| Yes | 60.1 (49.6-69.5)^*^ | 57.3 (49.8-64.7)^*^ | 51.6 (34.6-78.1)^*^ | 61.6 (49.7-70.6)^*^ | N.A. | 61.0 (49.0-74.0)^*^ | N.A. | N.A. | N.A. |
| PTH, in pmol/L |  |  |  |  |  |  |  |  |  |
| <6.8 | 52.8 (39.8-66.6)^*^ | 52.3 (45.4-59.6)^*^ | 39.5 (25.8-57.3)^*^ | 60.2 (45.2-69.4)^*^ | N.A. | 58.0 (45.0-74.0)^*^ | 57.6 (40.4-89.4)^*^ | 49.6 (36.5-62.5)^*^ | 55.9 (43.8-69.6)^*^ |
| ≥6.8 | 41.0 (27.8-56.7)^*^ | 47.3 (40.6-52.5)^*^ | 25.3 (18.1-38.0)^*^ | 39.9 (29.4-55.9)^*^ | N.A. | 47.5 (32.0-62.8)^*^ | 37.9 (24.4-58.8)^*^ | 35.7 (22.4-46.2)^*^ | 48.1 (36.6-62.5)^*^ |

Characteristics are presented as median 25-Hydroxy-vitamin D [25(OH)D] values with interquartile range. Abbreviations: Tromsø = Tromsø Study; LURIC = Ludwigshafen RIsk and Cardiovascular Health Study; AGES = Age, Gene/Environment Susceptibility Reykjavik Study; NHS = New Hoorn Study; Aarhus = Aarhus Mammography Cohort Study; DEGS = German Health Interview and Examination Survey for Adults; LASA = Longitudinal Aging Study Amsterdam; BMI = Body mass index; HTN = Arterial hypertension; SBP = Systolic blood pressure; LDL = Low density lipoprotein; CVD = Cardiovascular disease; eGFR = Estimated glomerular filtration rate according to the four-variable Modification of Diet in Renal Disease (MDRD) formula; CRP = C-reactive protein; PTH = Parathyroid hormone; N.A. = Not available; *Statistically significant (P<0.05) by analysis of variance. ^a^Season of baseline blood sampling was defined as spring (March to May), summer (June to August), autumn (September to November), and winter (December to February). ^b^Definition of categories of BMI by World Health Organization BMI categories: normal or underweight (<25 kg/m²), overweight (25 to <30 kg/m²); obesity (≥30 kg/m²). ^c^Physical activity was defined as frequency of medium- or vigorous leisure activity and was stratified in low (<1 hour per week), medium (1-3 hours) and high frequency (>3 hours per week). ^d^Diabetes mellitus at baseline was defined as (listed according to priority - highest priority first): Those participants on glucose lowering drugs, physician-reported, self-reported or according to ADA: fasting glucose ≥ 7.0 mmol/L, 2h postload glucose ≥ 11.1 mmol/L or HbA1c ≥ 6.5% (ICD-9: 250; ICD-10: E10-E14). ^e^Arterial hypertension at baseline was defined as (listed according to priority - highest priority first): Participants already on antihypertensive drug treatment, physician-reported, self-reported HTN, office systolic and/or diastolic blood pressure of equal to or higher than 140 and/or 90 mm Hg (ICD-9: 401,405; ICD-10: I10,I15). ^f^History of CVD at baseline was defined as positive history of myocardial infarction and/or stroke.

**Supplementary Table D.** Description of Follow-Up time in Cohort Studies and Mortality Outcomes.

| Study name | End of follow-up, Year | Median  follow-up  time, years | Participants at risk, N | Total  Deaths, N | CVD  Deaths, N | Cancer  Deaths, N | Other  Deaths, N | Unspecified  Deaths, N |
| --- | --- | --- | --- | --- | --- | --- | --- | --- |
| Overall | - | 10.5 | 26916 | 6802^†^ | 1810 | 1414 | 1450 | 958 |
| Tromsø | 2013 | 17.8 | 7145 | 2203 | 665 | 661 | N.A. | 877 |
| LURIC | 2010 | 9.9 | 3299 | 985 | 613 | 141 | 210 | 21 |
| AGES | 2013^†^ | 8.9 | 5510 | 2146^†^ | 252 | 307 | 487 | 2 |
| NHS | 2014 | 7.5 | 2591 | 69 | N.A.^‡^ | N.A.^‡^ | N.A.^‡^ | N.A.^‡^ |
| Aarhus | 2014 | 8.7 | 2473 | 78 | 11 | 56 | 10 | 1 |
| DEGS^*^ | 2010 | 12.1 | 3862 | 282 | 89 | 85 | 55 | 53 |
| LASA, first cohort | 2013 | 14.4 | 1302 | 973 | 175 | 153 | 638 | 6 |
| LASA, second cohort | 2013 | 10.8 | 734 | 66 | 5 | 11 | 50 | 0 |

Characteristics are presented as median follow-up time and total number (N) of death where appropriate. Abbreviations: Tromsø = Tromsø Study; LURIC = Ludwigshafen RIsk and Cardiovascular Health Study; AGES = Age, Gene/Environment Susceptibility Reykjavik Study; NHS = New Hoorn Study; Aarhus = Aarhus Mammography Cohort Study; DEGS = German Health Interview and Examination Survey for Adults; LASA = Longitudinal Aging Study Amsterdam; 25(OH)D=25-hydroxyvitamin D; CVD death = Cardiovascular death; CLIA = ChemiLuminescent ImmunoAssay; LC-MS = Liquid chromatography-mass spectrometry; RIA = radioimmunoassay; N.A. = not available; ^*^For the present analysis, the German National Health Interview and Examination Survey 1998 (GNHIES98) with the integrated German Nutrition Survey 1998 (GeNuS98) was the baseline visit. **^†^**For AGES, follow-up for death of all causes extends through end of year 2013, but cause specific death was coded through the year 2009. Due to the difference in follow-up time the number of total deaths and cause specific deaths differ. ^‡^For NHS only data on all-cause mortality was available.

# Supplementary Table E. Baseline Characteristics of Individuals Alive and Deceased.

| Characteristic | Alive  (n = 20114) | Deceased  (n = 6802) |
| --- | --- | --- |
| Age, years | 57.9 (48.3-67.4)^*^ | 72.6 (66.4-79.0)^*^ |
| Sex, % women | 62^*^ | 46^*^ |
| Season |  |  |
| Winter, % | 26.6^*^ | 27.0^*^ |
| Spring, % | 26.5^*^ | 14.0^*^ |
| Summer, % | 16.6^*^ | 30.3^*^ |
| Autumn, % | 30.3^*^ | 30.3^*^ |
| BMI, kg/m² | 25.8 (23.7-28.7)^*^ | 26.2 (23.7-29.1)^*^ |
| Current smoking, % | 24 | 25 |
| Physical activity |  |  |
| Low frequency, % | 53.7^*^ | 73.7^*^ |
| Medium frequency, % | 31.5^*^ | 19.5^*^ |
| High frequency, % | 14.8^*^ | 6.8^*^ |
| Present Diabetes, % | 9^*^ | 17^*^ |
| Glucose, mmHg | 5.4 (4.8-6-0)^*^ | 5.5 (5.1-6.2)^*^ |
| Present HTN, % | 53^*^ | 80^*^ |
| SBP, mmHg | 137.0 (124.0-151.5)^*^ | 146.0 (131.0-164.0)^*^ |
| LDL, mmol/L | 3.6 (2.9-4.4)^*^ | 3.7 (2.9-4.5)^*^ |
| History of CVD, % | 10^*^ | 24^*^ |
| History of cancer, % | 5^*^ | 14^*^ |
| eGFR, mL/min/1.73m² | 85.9 (72.5-98.9)^*^ | 75.7 (61.8-92.2)^*^ |
| CRP, mg/dL | 1.7 (0.8-3.8)^*^ | 2.7 (2.2-6.1)^*^ |
| Calcium Supplements, % | 12^*^ | 12^*^ |
| Vitamin D Supplements, % | 46^*^ | 50^*^ |
| PTH, pmol/L | 3.5 (2.5-4.7)^*^ | 3.5 (2.5-4.8)^*^ |
| Original 25(OH)D, nmol/L | 53.6 (38.3-70.5)^*^ | 49.7 (33.9-66.0)^*^ |
| Standardized 25(OH)D, nmol/L | 55.4 (42.8-68.1)^*^ | 50.9 (38.2-62.7)^*^ |

Characteristics are presented as median with interquartile range or percentage where appropriate. Abbreviations: BMI = Body mass index; HTN = Arterial hypertension; SBP = Systolic blood pressure; LDL = Low density lipoprotein; CVD = Cardiovascular disease; eGFR = Estimated glomerular filtration rate according to the four-variable Modification of Diet in Renal Disease (MDRD) formula; CRP = C-reactive protein; PTH = Parathyroid hormone; 25(OH)D = 25-Hydroxy-vitamin D; N.A. = Not available; *Statistically significant (P<0.05) by analysis of variance or chi square test. ^a^Season of baseline blood sampling was defined as spring (March to May), summer (June to August), autumn (September to November), and winter (December to February). ^b^Physical activity was defined as frequency of medium- or vigorous leisure activity and was stratified in low (<1 hour per week), medium (1-3 hours) and high frequency (>3 hours per week). ^c^Diabetes mellitus at baseline was defined as (listed according to priority - highest priority first): Those participants on glucose lowering drugs, physician-reported, self-reported or according to ADA: fasting glucose ≥ 7.0 mmol/L, 2h postload glucose ≥ 11.1 mmol/L or HbA1c ≥ 6.5% (ICD-9: 250; ICD-10: E10-E14). ^d^Arterial hypertension at baseline was defined as (listed according to priority - highest priority first): Participants already on antihypertensive drug treatment, physician-reported, self-reported HTN, office systolic and/or diastolic blood pressure of equal to or higher than 140 and/or 90 mm Hg (ICD-9: 401,405; ICD-10: I10,I15). History of CVD at baseline was defined as positive history of myocardial infarction and/or stroke.

**Supplementary Table F.** Additional Adjustments for Hazard Ratios of Death From All Causes (95% CI) by Standardized Total 25-Hydroxy-Vitamin D Concentrations in nmol/L and Statistical Approach.

|  | Category | <30 | 30-39.99 | 40-49.99 | 50-74.99 | 75-99.99 | 100-124.99 | ≥125 |
| --- | --- | --- | --- | --- | --- | --- | --- | --- |
| Additional model 1^a^ | Plus adjustment for intake of calcium^a^ | 1.78 (1.47-2.09) | 1.36 (1.13-1.60) | 1.19 (0.99-1.39) | 1.09 (0.93-1.25) | 1.00 | 1.03 (0.53-1.54) | 1.18 (0.00-2.70) |
| Additional model 2^b^ | Plus adjustment for intake of vitamin D^b^ | 1.82 (1.42-2.22) | 1.37 (1.08-1.67) | 1.17 (0.94-1.41) | 1.06 (0.87-1.25) | 1.00 | 0.96 (0.38-1.55) | 1.02 (0.00-2.58) |
| Additional model 3^c^ | Plus adjustment for PA^c^ | 1.64 (1.42-1.86) | 1.32 (1.14-1.49) | 1.12 (0.98-1.26) | 1.05 (0.93-1.17) | 1.00 | 1.00 (0.66-1.33) | 0.98 (0.27-1.69) |
| Additional model 4^d^ | Plus adjustment for eGFR^d^ | 1.70 (1.46-1.94) | 1.35 (1.17-1.54) | 1.17 (1.01-1.32) | 1.06 (0.93-1.18) | 1.00 | 1.03 (0.67-1.40) | 1.01 (0.25-1.77) |
| Additional model 5^e^ | Plus adjustment for PTH^e^ | 1.75 (1.43-2.08) | 1.41 (1.15-1.67) | 1.22 (1.00-1.44) | 1.14 (0.95-1.33) | 1.00 | 1.04 (0.57-1.52) | 1.03 (0.22-1.84) |
| Additional model 6^f^ | Plus adjustment for CRP^f^ | 1.67 (1.34-2.00) | 1.31 (1.04-1.57) | 1.14 (0.91-1.37) | 1.06 (0.87-1.25) | 1.00 | 1.00 (0.52-1.48) | 0.99 (0.00-2.01) |
| Additional model 7^g^ | Plus adjustment for SBP^g^ | 1.66 (1.42-1.89) | 1.31 (1.13-1.49) | 1.14 (0.99-1.28) | 1.05 (0.92-1.17) | 1.00 | 1.00 (0.65-1.35) | 0.98 (0.25-1.71) |
| Additional model 8^h^ | Plus adjustment for LDL-C^h^ | 1.67 (1.44-1.91) | 1.30 (1.12-1.49) | 1.14 (1.00-1.29) | 1.05 (0.92-1.17) | 1.00 | 0.99 (0.64-1.34) | 0.99 (0.25-1.73) |
| Additional model 9^i^ | Plus adjustment for glucose^i^ | 1.73 (1.44-2.02) | 1.31 (1.09-1.54) | 1.11 (0.92-1.30) | 1.08 (0.92-1.24) | 1.00 | 0.98 (0.60-1.37) | 1.02 (0.18-1.85) |

Statistical approach was based on categorical models. Categories are based on the Institute of Medicine report 2011 used cut-off values. Abbreviations: DEGS = German Health Interview and Examination Survey for Adults; NHS = The New Hoorn Study; HR = Hazard ratio with 95% confidence interval (CI).

^a^Additional cumulative adjustment for supplemental intake of calcium (yes/no) after adjustment for age, sex, season of blood drawing and BMI. Due to missing data on supplemental intake of calcium, the analysis was performed without the New Hoorn Study (NHS), and Longitudinal Aging Study Amsterdam (LASA), second cohort.

^b^Additional cumulative adjustment for supplemental intake of vitamin D (yes/no) after adjustment for age, sex, season of blood drawing and BMI. Due to missing data on supplemental intake of vitamin D, the analysis was performed without NHS, and LASA, first and second cohort.

^c^Additional cumulative adjustment for physical activity (PA), either low frequency (yes/no), medium (yes/no) or high frequency (yes/no), after adjustment for age, sex, season of blood drawing and BMI. Due to missing data on physical activity, the analysis was performed without the Aarhus Mammography Cohort Study.

^d^Additional cumulative adjustment for estimated glomerular filtration rate (eGFR) in mL/min/1.73m² according to the four-variable Modification of Diet in Renal Disease formula, after adjustment for age, sex, season of blood drawing and BMI. Due to missing data on eGFR, the analysis was performed without NHS.

^e^Additional cumulative adjustment for parathyroid hormone (PTH) in pmol/L, after adjustment for age, sex, season of blood drawing and BMI. Due to missing data on PTH, the analysis was performed without NHS.

^f^Additional cumulative adjustment for C-reactive protein (CRP) in mg/L, after adjustment for age, sex, season of blood drawing and BMI. Due to missing data on CRP, the analysis was performed without the New Hoorn Study, Aarhus Mammography Cohort Study and LASA, second cohort.

^g^Additional cumulative adjustment for systolic blood pressure (SBP) in mm Hg, after adjustment for age, sex, season of blood drawing and BMI. Due to missing data on SBP, the analysis was performed without the Aarhus Mammography Cohort Study.

^h^Additional cumulative adjustment for low-density lipoprotein cholesterol (LDL-C) in mmol/L, after adjustment for age, sex, season of blood drawing and BMI. Due to missing data on LDL-C, the analysis was performed without the Aarhus Mammography Cohort Study.

^i^Additional cumulative adjustment for glucose in mmol/L, after adjustment for age, sex, season of blood drawing and BMI.

# Supplementary Table G. Adjusted Hazard Ratio of Death From All Causes (95% CI) by Standardized 25-Hydroxyvitamin D Concentrations in nmol/L and Statistical Approach For Full Database Without the New Hoorn Study.

|  | Category | <30 | 30-39.99 | 40-49.99 | 50-74.99 | 75-99.99 | 100-124.99 | ≥125 |
| --- | --- | --- | --- | --- | --- | --- | --- | --- |
|  | Median*, nmol/L | 22.8 | 35.9 | 45.3 | 60.2 | 83.7 | 107.1 | 135.0 |
|  | Sample size, n | 2716 | 2853 | 4638 | 10717 | 2648 | 592 | 161 |
|  | Deaths, n | 990 | 887 | 1377 | 2905 | 508 | 55 | 11 |
| Model 1^a^ | Categorical HR (95% CI) | 1.65 (1.42-1.88) | 1.33 (1.14-1.51) | 1.14 (0.99-1.29) | 1.05 (0.92-1.17) | 1.00 | 1.00 (0.65-1.36) | 0.98 (0.25-1.72) |
|  | Cubic-splines HR (95% CI) | 1.74 (1.56-1.93) | 1.28 (1.16-1.40) | 1.13 (1.02-1.24) | 1.05 (0.96-1.14) | 1.00 | 1.06 (0.92-1.20) | 1.13 (0.80-1.46) |
|  | Nadir, nmol/L (95% CI) | 78.5 (67.6-89.4) |  |  |  |  |  |  |
| Model 2^b^ | Categorical HR (95% CI) | 1.67 (1.44-1.91) | 1.34 (1.16-1.53) | 1.15 (1.00-1.30) | 1.05 (0.93-1.18) | 1.00 | 1.01 (0.65-1.36) | 0.99 (0.25-1.73) |
|  | Cubic-splines HR (95% CI) | 1.76 (1.57-1.96) | 1.29 (1.17-1.42) | 1.14 (1.03-1.25) | 1.05 (0.96-1.15) | 1.00 | 1.05 (0.91-1.19) | 1.12 (0.79-1.45) |
|  | Nadir, nmol/L (95% CI) | 78.9 (67.0-90.9) |  |  |  |  |  |  |
| Model 3^c^ | Categorical HR (95% CI) | 1.62 (1.39-1.84) | 1.33 (1.14-1.51) | 1.15 (1.00-1.30) | 1.06 (0.94-1.19) | 1.00 | 1.03 (0.67-1.39) | 0.98 (0.23-1.73) |
|  | Cubic-splines HR (95% CI) | 1.71 (1.52-1.91) | 1.27 (1.15-1.39) | 1.13 (1.01-1.24) | 1.05 (0.96-1.13) | 1.00 | 1.06 (0.91-1.20) | 1.14 (0.75-1.54) |
|  | Nadir, nmol/L (95% CI) | 78.7 (67.6-89.8) |  |  |  |  |  |  |
| Model 4^d^ | Categorical HR (95% CI) | 1.50 (1.28-1.71) | 1.24 (1.07-1.42) | 1.12 (0.97-1.27) | 1.05 (0.92-1.18) | 1.00 | 1.07 (0.69-1.45) | 0.87 (0.21-1.53) |
|  | Cubic-splines HR (95% CI) | 1.56 (1.38-1.74) | 1.19 (1.08-1.31) | 1.08 (0.97-1.19) | 1.04 (0.95-1.12) | 1.00 | 1.04 (0.89-1.18) | 1.10 (0.72-1.49) |
|  | Nadir, nmol/L (95% CI) | 78.6 (69.3-88.0) |  |  |  |  |  |  |

# Statistical approach was based on 1) categorical models and 2) cubic splines models. Estimates of the cubic splines approach were calculated for the median 25-hydroxyvitamin D value (*) of each category. Categories are based on the Institute of Medicine report 2011 used cut-off values. Abbreviations: HR = Hazard ratio with 95% confidence interval (CI). The nadir is the level of 25-hydroxyvitamin D with the lowest predicted risk. ^a^Adjusted for age, sex, and season of blood drawing. ^b^Adjusted for age, sex, season of blood drawing, and body mass index (BMI). ^c^Adjusted for age, sex, season of blood drawing, BMI, diabetes mellitus at baseline, and arterial hypertension at baseline. ^d^Adjusted for age, sex, season of blood drawing, BMI, active smoker status, history of cardiovascular disease (CVD), and history of cancer. History of CVD was defined as history of myocardial infarction and/or history of stroke.

# Supplementary Table H. Adjusted Hazard Ratio of Death From All Causes (95% CI) by Original 25-Hydroxyvitamin D Concentrations in nmol/L and Statistical Approach For Full Database Without The New Hoorn Study.

|  | Category | <30 | 30-39.99 | 40-49.99 | 50-74.99 | 75-99.99 | 100-124.99 | ≥125 |
| --- | --- | --- | --- | --- | --- | --- | --- | --- |
|  | Median*, nmol/L | 22.3 | 35.5 | 45.2 | 61.0 | 83.9 | 108.0 | 143.0 |
|  | Sample size, n | 3740 | 3417 | 3994 | 8616 | 3390 | 853 | 315 |
|  | Deaths, n | 1355 | 982 | 1066 | 2320 | 792 | 170 | 48 |
| Model 1^a^ | Categorical HR (95% CI) | 1.45 (1.29-1.61) | 1.11 (0.98-1.24) | 0.98 (0.87-1.09) | 1.01(0.91-1.11) | 1.00 | 1.12 (0.90-1.34) | 1.26(0.82-1.71) |
|  | Cubic-splines HR (95% CI) | 1.40 (1.27-1.53) | 1.04 (0.95-1.13) | 0.97 (0.88-1.06) | 0.98 (0.91-1.06) | 1.00 | 1.06 (0.97-1.15) | 1.16 (0.91-1.41) |
|  | Nadir, nmol/L (95% CI) | 43.4 (37.2-49.5) |  |  |  |  |  |  |
| Model 2^b^ | Categorical HR (95% CI) | 1.46 (1.30-1.63) | 1.12 (0.99-1.25) | 0.99 (0.88-1.10) | 1.01 (0.92-1.11) | 1.00 | 1.12 (0.90-1.34) | 1.26 (0.82-1.70) |
|  | Cubic-splines HR (95% CI) | 1.41 (1.28-1.54) | 1.05 (0.96-1.15) | 0.98 (0.89-1.07) | 0.98 (0.91-1.06) | 1.00 | 1.06 (0.97-1.15) | 1.15 (0.90-1.40) |
|  | Nadir, nmol/L (95% CI) | 44.1 (35.8-52.4) |  |  |  |  |  |  |
| Model 3^c^ | Categorical HR(95% CI) | 1.41 (1.25-1.57) | 1.10 (0.97-1.23) | 0.97 (0.86-1.08) | 1.01 (0.91-1.11) | 1.00 | 1.10 (0.88-1.32) | 1.28 (0.83-1.74) |
|  | Cubic-splines HR (95% CI) | 1.36 (1.24-1.49) | 1.03 (0.94-1.13) | 0.97 (0.88-1.06) | 0.98 (0.91-1.06) | 1.00 | 1.06 (0.96-1.15) | 1.15 (0.89-1.40) |
|  | Nadir, nmol/L (95% CI) | 43.6 (36.4-50.7) |  |  |  |  |  |  |
| Model 4^d^ | Categorical HR (95% CI) | 1.47 (1.29-1.64) | 1.20 (1.06-1.35) | 1.08 (0.95-1.20) | 1.08 (0.97-1.19) | 1.00 | 1.10 (0.87-1.33) | 1.31 (0.83-1.80) |
|  | Cubic-splines HR (95% CI) | 1.40 (1.27-1.53) | 1.11 (1.01-1.21) | 1.06 (0.96-1.16) | 1.04 (0.96-1.12) | 1.00 | 1.06 (0.96-1.15) | 1.15 (0.88-1.42) |
|  | Nadir, nmol/L (95% CI) | 83.2 (75.9-90.5) |  |  |  |  |  |  |

# Statistical approach was based on 1) categorical models and 2) cubic splines models. Estimates of the cubic splines approach were calculated for the median 25-hydroxyvitamin D value (*) of each category. Categories are based on the Institute of Medicine report 2011 used cut-off values. Abbreviations: HR = Hazard ratio with 95% confidence interval (CI). The nadir is the level of 25-hydroxyvitamin D with the lowest predicted risk. ^a^Adjusted for age, sex, and season of blood drawing. ^b^Adjusted for age, sex, season of blood drawing, and body mass index (BMI). ^c^Adjusted for age, sex, season of blood drawing, BMI, diabetes mellitus at baseline, and arterial hypertension at baseline. ^d^Adjusted for age, sex, season of blood drawing, BMI, active smoker status, history of cardiovascular disease (CVD), and history of cancer. History of CVD was defined as history of myocardial infarction and/or history of stroke.

# Supplementary Table I. Adjusted Hazard Ratio of Death From All Causes (95% CI) by Standardized Versus Original 25-Hydroxyvitamin D Concentrations in nmol/L for the Tromsø Study.

|  | Category | <30 | 30-39.99 | 40-49.99 | 50-74.99 | 75-99.99 | ≥100 |
| --- | --- | --- | --- | --- | --- | --- | --- |
|  | Median*, nmol/L | 27.8 | 37.2 | 45.8 | 58.5 | 79.7 | 112.0 |
|  | Sample size, n | 49 | 572 | 2019 | 4173 | 317 | 15 |
|  | Deaths, n | 19 | 211 | 700 | 1193 | 77 | 3 |
| Standardized measurements | |  |  |  |  |  |  |
| Model 1^a^ | Categorical HR (95% CI) | 1.24 (0.60-1.88) | 1.19 (0.88-1.50) | 0.98 (0.75-1.21) | 0.89 (0.68-1.09) | 1.00 | 1.07 (0.00-2.29) |
| Model 2^b^ | Categorical HR (95% CI) | 1.26 (0.61-1.91) | 1.21 (0.89-1.53) | 0.99 (0.76-1.22) | 0.89 (0.69-1.09) | 1.00 | 1.08 (0.00-2.30) |
| Model 3^c^ | Categorical HR (95% CI) | 1.20 (0.58-1.82) | 1.23 (0.90-1.56) | 1.02 (0.78-1.27) | 0.93 (0.72-1.15) | 1.00 | 1.12 (0.00-2.37) |
| Model 4^d^ | Categorical HR (95% CI) | 1.12 (0.54-1.70) | 1.11 (0.82-1.41) | 0.96 (0.73-1.19) | 0.89 (0.69-1.10) | 1.00 | 1.14 (0.00-2.48) |
| Original measurements | |  |  |  |  |  |  |
| Model 1^a^ | Categorical HR (95% CI) | 0.96 (0.76-1.16) | 0.86 (0.72-1.00) | 0.73 (0.62-0.84) | 0.85 (0.74-0.95) | 1.00 | 1.28 (0.96-1.61) |
| Model 2^b^ | Categorical HR (95% CI) | 0.97 (0.77-1.18) | 0.87 (0.72-1.01) | 0.73 (0.62-0.85) | 0.85 (0.74-0.95) | 1.00 | 1.28 (0.95-1.61) |
| Model 3^c^ | Categorical HR (95% CI) | 0.91 (0.72-1.11) | 0.85 (0.71-0.99) | 0.71 (0.60-0.82) | 0.84 (0.73-0.95) | 1.00 | 1.26 (0.93-1.58) |
| Model 4^d^ | Categorical HR (95% CI) | 1.44 (1.12-1.76) | 1.35 (1.11-1.59) | 1.07 (0.90-1.25) | 1.05 (0.92-1.19) | 1.00 | 1.12(0.83-1.41) |

Categories are based on the Institute of Medicine report 2011 used cut-off values. Category characteristics refer to standardized 25-hydroxyvitamin D concentrations. Abbreviations: HR = Hazard ratio with 95% confidence interval (CI); *The median is the median standardized 25-hydroxyvitamin D value of each category. ^a^Adjusted for age, sex, and season of blood drawing. ^b^Adjusted for age, sex, season of blood drawing, and body mass index (BMI). ^c^Adjusted for age, sex, season of blood drawing, BMI, diabetes mellitus at baseline, and arterial hypertension at baseline. ^d^Adjusted for age, sex, season of blood drawing, BMI, active smoker status, history of cardiovascular disease (CVD), and history of cancer. History of CVD was defined as history of myocardial infarction and/or history of stroke.

# Supplementary Table J. Adjusted Hazard Ratio of Death From All Causes (95% CI) by Standardized Versus Original 25-Hydroxyvitamin D Concentrations in nmol/L for the Ludwigshafen RIsk and Cardiovascular Health Study.

|  | Category | <30 | 30-39.99 | 40-49.99 | 50-74.99 | 75-99.99 | ≥100 |
| --- | --- | --- | --- | --- | --- | --- | --- |
|  | Median*, nmol/L | 20.9 | 35.1 | 45.0 | 59.5 | 84.3 | 108.4 |
|  | Sample size, n | 1123 | 609 | 463 | 791 | 253 | 60 |
|  | Deaths, n | 477 | 173 | 116 | 175 | 37 | 7 |
| Standardized measurements | |  |  |  |  |  |  |
| Model 1^a^ | Categorical HR (95% CI) | 2.92 (1.98-3.85) | 1.73 (1.14-2.31) | 1.46 (0.95-1.97) | 1.27 (0.85-1.70) | 1.00 | 0.63 (0.15-1.11) |
| Model 2^b^ | Categorical HR (95% CI) | 2.94 (1.99-3.89) | 1.74 (1.15-2.34) | 1.48 (0.96-2.00) | 1.28 (0.85-1.71) | 1.00 | 0.67 (0.18-1.17) |
| Model 3^c^ | Categorical HR (95% CI) | 2.69 (1.82-3.56) | 1.61 (1.06-2.15) | 1.37 (0.89-1.85) | 1.25 (0.83-1.67) | 1.00 | 0.70 (0.18-1.22) |
| Model 4^d^ | Categorical HR (95% CI) | 2.63 (1.76-3.48) | 1.57 (1.04-2.10) | 1.41 (0.91-1.90) | 1.25 (0.83-1.68) | 1.00 | 0.71 (0.19-1.24) |
| Original measurements | |  |  |  |  |  |  |
| Model 1^a^ | Categorical HR (95% CI) | 2.92 (1.98-3.85) | 1.73 (1.14-2.31) | 1.46 (0.95-1.97) | 1.27 (0.85-1.70) | 1.00 | 0.63 (0.15-1.11) |
| Model 2^b^ | Categorical HR (95% CI) | 2.94 (1.99-3.89) | 1.74 (1.15-2.33) | 1.47 (0.96-1.99) | 1.28 (0.85-1.70) | 1.00 | 0.63 (0.16-1.11) |
| Model 3^c^ | Categorical HR (95% CI) | 2.70 (1.83-3.58) | 1.62 (1.07-2.16) | 1.38 (0.89-1.86) | 1.25 (0.83-1.67) | 1.00 | 0.67 (0.16-1.78) |
| Model 4^d^ | Categorical HR (95% CI) | 2.62 (1.77-3.46) | 1.57 (1.04-2.10) | 1.40 (0.91-1.90) | 1.25 (0.83-1.66) | 1.00 | 0.67 (0.16-1.17) |

Categories are based on the Institute of Medicine report 2011 used cut-off values. Category characteristics refer to standardized 25-hydroxyvitamin D concentrations. Abbreviations: HR = Hazard ratio with 95% confidence interval (CI); *The median is the median standardized 25-hydroxyvitamin D value of each category. ^a^Adjusted for age, sex, and season of blood drawing. ^b^Adjusted for age, sex, season of blood drawing, and body mass index (BMI). ^c^Adjusted for age, sex, season of blood drawing, BMI, diabetes mellitus at baseline, and arterial hypertension at baseline. ^d^Adjusted for age, sex, season of blood drawing, BMI, active smoker status, history of cardiovascular disease (CVD), and history of cancer. History of CVD was defined as history of myocardial infarction and/or history of stroke.

# Supplementary Table K . Adjusted Hazard Ratio of Death From All Causes (95% CI) by Standardized Versus Original 25-Hydroxyvitamin D Concentrations in nmol/L for the Age, Gene/Environment Susceptibility Reykjavik Study.

|  | Category | <30 | 30-39.99 | 40-49.99 | 50-74.99 | 75-99.99 | ≥100 |
| --- | --- | --- | --- | --- | --- | --- | --- |
|  | Median*, nmol/L | 24.9 | 35.5 | 45.0 | 62.7 | 81.3 | 110.1 |
|  | Sample size, n | 460 | 622 | 767 | 2881 | 722 | 58 |
|  | Deaths, n | 218 | 266 | 289 | 1075 | 276 | 22 |
| Standardized measurements | |  |  |  |  |  |  |
| Model 1^a^ | Categorical HR (95% CI) | 1.48 (1.21-1.74) | 1.32 (1.10-1.55) | 1.05 (0.88-1.23) | 1.04 (0.90-1.18) | 1.00 | 1.29 (0.73-1.85) |
| Model 2^b^ | Categorical HR (95% CI) | 1.51 (1.28-1.75) | 1.22 (1.02-1.43) | 1.09 (0.91-1.26) | 1.11 (0.96-1.27) | 1.00 | 1.35 (1.02-1.68) |
| Model 3^c^ | Categorical HR (95% CI) | 1.49 (1.26-1.72) | 1.22 (1.01-1.42) | 1.09 (0.91-1.27) | 1.12 (0.96-1.27) | 1.00 | 1.33 (1.00-1.67) |
| Model 4^d^ | Categorical HR (95% CI) | 1.38 (1.16-1.60) | 1.18 (0.98-1.39) | 1.04 (0.87-1.22) | 1.13 (0.97-1.29) | 1.00 | 1.42 (1.07-1.78) |
| Original measurements | |  |  |  |  |  |  |
| Model 1^a^ | Categorical HR (95% CI) | 1.50 (1.27-1.73) | 1.21 (1.01-1.41) | 1.08 (0.90-1.25) | 1.11 (0.96-1.27) | 1.00 | 1.37 (1.03-1.70) |
| Model 2^b^ | Categorical HR (95% CI) | 1.53 (1.29-1.77) | 1.23 (1.03-1.44) | 1.09 (0.91-1.27) | 1.12 (0.96-1.28) | 1.00 | 1.36 (1.03-1.70) |
| Model 3^c^ | Categorical HR (95% CI) | 1.53 (1.29-1.76) | 1.22 (1.02-1.43) | 1.10 (0.92-1.28) | 1.12 (0.97-1.28) | 1.00 | 1.36 (1.02-1.69) |
| Model 4^d^ | Categorical HR (95% CI) | 1.39 (1.17-1.61) | 1.18 (0.98-1.38) | 1.05 (0.87-1.23) | 1.13 (0.97-1.29) | 1.00 | 1.40 (1.05-1.76) |

Categories are based on the Institute of Medicine report 2011 used cut-off values. Category characteristics refer to standardized 25-hydroxyvitamin D concentrations. Abbreviations: HR = Hazard ratio with 95% confidence interval (CI); *The median is the median standardized 25-hydroxyvitamin D value of each category. ^a^Adjusted for age, sex, and season of blood drawing. ^b^Adjusted for age, sex, season of blood drawing, and body mass index (BMI). ^c^Adjusted for age, sex, season of blood drawing, BMI, diabetes mellitus at baseline, and arterial hypertension at baseline. ^d^Adjusted for age, sex, season of blood drawing, BMI, active smoker status, history of cardiovascular disease (CVD), and history of cancer. History of CVD was defined as history of myocardial infarction and/or history of stroke.

# Supplementary Table L. Adjusted Hazard Ratio of Death From All Causes (95% CI) by Newly Measured 25-Hydroxyvitamin D Concentrations in nmol/L for the New Hoorn Study.

|  | Category | <30 | 30-39.99 | 40-49.99 | 50-74.99 | 75-99.99 | ≥100 |
| --- | --- | --- | --- | --- | --- | --- | --- |
|  | Median*, nmol/L | 24.8 | 35.5 | 45.4 | 62.2 | 83.1 | 108.4 |
|  | Sample size, n | 235 | 253 | 380 | 1148 | 477 | 98 |
|  | Deaths, n | 9 | 5 | 9 | 30 | 14 | 2 |
| Model 1^a^ | Categorical HR (95% CI) | 1.85 (0.20-3.49) | 0.88 (0.00-1.81) | 0.91 (0.13-1.69) | 0.97 (0.35-1.59) | 1.00 | 0.76 (0.00-1.88) |
| Model 2^b^ | Categorical HR (95% CI) | 1.44 (0.00-4.34) | 0.74 (0.69-0.79) | 0.76 (0.72-0.81) | 0.89 (0.87-0.92) | 1.00 | 0.77 (0.73-0.82) |
| Model 3^c^ | Categorical HR (95% CI) | 1.41 (0.00-4.21) | 0.73 (0.68-0.78) | 0.74 (0.69-0.79) | 0.90 (0.88-0.92) | 1.00 | 0.78 (0.74-0.83) |
| Model 4^d^ | Categorical HR (95% CI) | 1.16 (0.00-3.61) | 0.65 (0.59-0.72) | 0.65 (0.59-0.72) | 0.81 (0.77-0.85) | 1.00 | 0.82 (0.78-0.86) |

Categories are based on the Institute of Medicine report 2011 used cut-off values. Category characteristics refer to standardized 25-hydroxyvitamin D concentrations. Abbreviations: HR = Hazard ratio with 95% confidence interval (CI); *The median is the median newly measured 25-hydroxyvitamin D value of each category. ^a^Adjusted for age, sex, and season of blood drawing. ^b^Adjusted for age, sex, season of blood drawing, and body mass index (BMI). ^c^Adjusted for age, sex, season of blood drawing, BMI, diabetes mellitus at baseline, and arterial hypertension at baseline. ^d^Adjusted for age, sex, season of blood drawing, BMI, active smoker status, history of cardiovascular disease (CVD), and history of cancer. History of CVD was defined as history of myocardial infarction and/or history of stroke.

# Supplementary Table M. Adjusted Hazard Ratio of Death From All Causes (95% CI) by Standardized Versus Original 25-Hydroxyvitamin D Concentrations in nmol/L for the Aarhus Mammography Cohort Study.

|  | Category | <30 | 30-39.99 | 40-49.99 | 50-74.99 | 75-99.99 | ≥100 |
| --- | --- | --- | --- | --- | --- | --- | --- |
|  | Median*, nmol/L | 25.0 | 36.0 | 46.0 | 62.0 | 84.0 | 110.0 |
|  | Sample size, n | 251 | 280 | 377 | 1048 | 412 | 105 |
|  | Deaths, n | 7 | 16 | 12 | 35 | 6 | 2 |
| Standardized measurements | |  |  |  |  |  |  |
| Model 1^a^ | Categorical HR (95% CI) | 2.39 (0.00-4.90) | 3.81 (0.47-7.15) | 2.28 (0.21-4.36) | 2.08 (0.55-3.61) | 1.00 | 1.28 (0.00-2.94) |
| Model 2^b^ | Categorical HR (95% CI) | 2.16 (0.00-4.45) | 3.33 (0.36-6.31) | 2.10 (0.18-4.02) | 2.03 (0.54-3.52) | 1.00 | 1.29 (0.00-2.95) |
| Model 3^c^ | Categorical HR (95% CI) | 2.17 (0.00-4.46) | 3.14 (0.33-5.96) | 2.13 (0.18-4.07) | 2.06 (0.55-3.58) | 1.00 | 1.32 (0.00-3.02) |
| Model 4^d^ | Categorical HR (95% CI) | 1.78 (0.00-3.77) | 3.00 (0.31-5.68) | 1.97 (0.16-3.77) | 2.07 (0.54-3.60) | 1.00 | 1.09 (0.00-2.53) |
| Original measurements | |  |  |  |  |  |  |
| Model 1^a^ | Categorical HR (95% CI) | 2.41 (0.00-4.91) | 3.77 (0.48-7.06) | 2.23 (0.21-4.26) | 2.03 (0.55-3.51) | 1.00 | 1.23 (0.00-2.83) |
| Model 2^b^ | Categorical HR (95% CI) | 2.20 (0.00-4.51) | 3.33 (0.36-6.30) | 2.07 (0.18-3.96) | 2.02 (0.54-3.50) | 1.00 | 1.23 (0.00-2.84) |
| Model 3^c^ | Categorical HR (95% CI) | 2.24 (0.00-4.58) | 3.16 (0.33-5.99) | 2.13 (0.19-4.07) | 2.04 (0.55-3.54) | 1.00 | 1.29 (0.00-2.97) |
| Model 4^d^ | Categorical HR (95% CI) | 1.83 (0.00-3.88) | 3.03 (0.32-5.75) | 1.97 (0.16-3.78) | 2.09 (0.55-3.63) | 1.00 | 1.07 (0.00-2.49) |

Categories are based on the Institute of Medicine report 2011 used cut-off values. Category characteristics refer to standardized 25-hydroxyvitamin D concentrations. Abbreviations: HR = Hazard ratio with 95% confidence interval (CI); *The median is the median standardized 25-hydroxyvitamin D value of each category. ^a^Adjusted for age, sex, and season of blood drawing. ^b^Adjusted for age, sex, season of blood drawing, and body mass index (BMI). ^c^Adjusted for age, sex, season of blood drawing, BMI, diabetes mellitus at baseline, and arterial hypertension at baseline. ^d^Adjusted for age, sex, season of blood drawing, BMI, active smoker status, history of cardiovascular disease (CVD), and history of cancer. History of CVD was defined as history of myocardial infarction and/or history of stroke.

# Supplementary Table N. Adjusted Hazard Ratio of Death From All Causes (95% CI) by Standardized Versus Original 25-Hydroxyvitamin D Concentrations in nmol/L for the German Health Interview and Examination Survey for Adults.

|  | Category | <30 | 30-39.99 | 40-49.99 | 50-74.99 | 75-99.99 | ≥100 |
| --- | --- | --- | --- | --- | --- | --- | --- |
|  | Median*, nmol/L | 22.2 | 34.9 | 44.7 | 59.7 | 89.9 | 110.9 |
|  | Sample size, n | 553 | 464 | 607 | 993 | 747 | 498 |
|  | Deaths, n | 59 | 43 | 44 | 59 | 51 | 26 |
| Standardized measurements | |  |  |  |  |  |  |
| Model 1^a^ | Categorical HR (95% CI) | 1.44 (0.88-2.01) | 1.28 (0.75-1.81) | 0.98 (0.58-1.38) | 0.84 (0.52-1.15) | 1.00 | 0.94 (0.49-1.39) |
| Model 2^b^ | Categorical HR (95% CI) | 1.42 (0.86-1.98) | 1.26 (0.74-1.79) | 0.96 (0.56-1.35) | 0.83 (0.52-1.14) | 1.00 | 0.95 (0.49-1.40) |
| Model 3^c^ | Categorical HR (95% CI) | 1.35 (0.82-1.89) | 1.24 (0.72-1.75) | 0.99 (0.58-1.41) | 0.83 (0.52-1.15) | 1.00 | 0.96 (0.50-1.42) |
| Model 4^d^ | Categorical HR (95% CI) | 1.31 (0.79-1.83) | 1.29 (0.74-1.84) | 1.00 (0.58-1.41) | 0.87 (0.53-1.20) | 1.00 | 0.97 (0.50-1.44) |
| Original measurements | |  |  |  |  |  |  |
| Model 1^a^ | Categorical HR (95% CI) | 1.62 (0.90-2.33) | 1.05 (0.55-1.55) | 0.80 (0.39-1.20) | 1.10 (0.59-1.60) | 1.00 | 1.04 (0.44-1.64) |
| Model 2^b^ | Categorical HR (95% CI) | 1.59 (0.88-2.30) | 1.03 (0.54-1.52) | 0.78 (0.28-1.17) | 1.09 (0.59-1.59) | 1.00 | 1.05 (0.44-1.65) |
| Model 3^c^ | Categorical HR (95% CI) | 1.51 (0.84-2.18) | 1.03 (0.54-1.52) | 0.77 (0.38-1.17) | 1.07 (0.58-1.56) | 1.00 | 1.06 (0.45-1.67) |
| Model 4^d^ | Categorical HR (95% CI) | 1.53 (0.85-2.22) | 1.10 (0.57-1.64) | 0.81 (0.39-1.22) | 1.18 (0.63-1.74) | 1.00 | 1.08 (0.44-1.72) |

Categories are based on the Institute of Medicine report 2011 used cut-off values. Category characteristics refer to standardized 25-hydroxyvitamin D concentrations. Abbreviations: HR = Hazard ratio with 95% confidence interval (CI); *The median is the median standardized 25-hydroxyvitamin D value of each category. ^a^Adjusted for age, sex, and season of blood drawing. ^b^Adjusted for age, sex, season of blood drawing, and body mass index (BMI). ^c^Adjusted for age, sex, season of blood drawing, BMI, diabetes mellitus at baseline, and arterial hypertension at baseline. ^d^Adjusted for age, sex, season of blood drawing, BMI, active smoker status, history of cardiovascular disease (CVD), and history of cancer. History of CVD was defined as history of myocardial infarction and/or history of stroke.

# Supplementary Table O. Adjusted Hazard Ratio of Death From All Causes (95% CI) by Standardized Versus Original 25-Hydroxyvitamin D Concentrations in nmol/L for Longitudinal Aging Study Amsterdam, first cohort.

|  | Category | <30 | 30-39.99 | 40-49.99 | 50-74.99 | 75-99.99 | ≥100 |
| --- | --- | --- | --- | --- | --- | --- | --- |
|  | Median*, nmol/L | 23.8 | 35.6 | 44.5 | 60.5 | 82.5 | 104.1 |
|  | Sample size, n | 224 | 205 | 260 | 502 | 104 | 7 |
|  | Deaths, n | 204 | 165 | 201 | 341 | 57 | 5 |
| Standardized measurements | |  |  |  |  |  |  |
| Model 1^a^ | Categorical HR (95% CI) | 1.63 (1.22-2.04) | 1.33 (0.98-1.68) | 1.30 (0.98-1.62) | 1.09 (0.84-1.33) | 1.00 | 1.06 (0.66-1.47) |
| Model 2^b^ | Categorical HR (95% CI) | 1.63 (1.21-2.04) | 1.34 (0.99-1.69) | 1.30 (0.98-1.62) | 1.09 (0.85-1.33) | 1.00 | 1.07 (0.66-1.47) |
| Model 3^c^ | Categorical HR (95% CI) | 1.51 (1.13-1.90) | 1.28 (0.94-1.62) | 1.21 (0.91-1.51) | 1.06 (0.82-1.30) | 1.00 | 1.03 (0.64-1.42) |
| Model 4^d^ | Categorical HR (95% CI) | 1.37 (1.02-1.72) | 1.21 (0.90-1.53) | 1.17 (0.88-1.46) | 1.05 (0.81-1.28) | 1.00 | 1.03 (0.64-1.43) |
| Original measurements | |  |  |  |  |  |  |
| Model 1^a^ | Categorical HR (95% CI) | 1.63 (1.22-2.04) | 1.33 (0.98-1.68) | 1.30 (0.98-1.62) | 1.09 (0.84-1.33) | 1.00 | 1.06 (0.66-1.47) |
| Model 2^b^ | Categorical HR (95% CI) | 1.64 (1.22-2.06) | 1.34 (0.99-1.69) | 1.30 (0.98-1.62) | 1.09 (0.85-1.33) | 1.00 | 1.06 (0.66-1.47) |
| Model 3^c^ | Categorical HR (95% CI) | 1.52 (1.13-1.90) | 1.28 (0.95-1.62) | 1.21 (0.91-1.51) | 1.06 (0.82-1.29) | 1.00 | 1.03 (0.64-1.42) |
| Model 4^d^ | Categorical HR (95% CI) | 1.37 (1.02-1.72) | 1.22 (0.90-1.54) | 1.17 (0.88-1.46) | 1.05 (0.81-1.28) | 1.00 | 1.03 (0.64-1.43) |

# Categories are based on the Institute of Medicine report 2011 used cut-off values. Category characteristics refer to standardized 25-hydroxyvitamin D concentrations. Abbreviations: HR = Hazard ratio with 95% confidence interval (CI); *The median is the median standardized 25-hydroxyvitamin D value of each category. ^a^Adjusted for age, sex, and season of blood drawing. ^b^Adjusted for age, sex, season of blood drawing, and body mass index (BMI). ^c^Adjusted for age, sex, season of blood drawing, BMI, diabetes mellitus at baseline, and arterial hypertension at baseline. ^d^Adjusted for age, sex, season of blood drawing, BMI, active smoker status, history of cardiovascular disease (CVD), and history of cancer. History of CVD was defined as history of myocardial infarction and/or history of stroke.

# Supplementary Table P. Adjusted Hazard Ratio of Death From All Causes (95% CI) by Standardized Versus Original 25-Hydroxyvitamin D Concentrations in nmol/L for Longitudinal Aging Study Amsterdam, second cohort.

|  | Category | <30 | 30-39.99 | 40-49.99 | 50-74.99 | 75-99.99 | ≥100 |
| --- | --- | --- | --- | --- | --- | --- | --- |
|  | Median*, nmol/L | 25.6 | 36.2 | 45.0 | 60.7 | 82.1 | 106.1 |
|  | Sample size, n | 56 | 101 | 145 | 329 | 93 | 10 |
|  | Deaths, n | 6 | 13 | 15 | 27 | 4 | 1 |
| Standardized measurements | |  |  |  |  |  |  |
| Model 1^a^ | Categorical HR (95% CI) | 3.00 (0.00-6.82) | 3.96 (0.00-8.44) | 2.68 (0.00-5.68) | 2.27 (0.00-4.67) | 1.00 | 2.58 (0.00-6.98) |
| Model 2^b^ | Categorical HR (95% CI) | 3.11 (0.00-7.08) | 4.11 (0.00-8.77) | 2.80 (0.00-5.92) | 2.32 (0.00-4.76) | 1.00 | 2.73 (0.00-7.40) |
| Model 3^c^ | Categorical HR (95% CI) | 2.99 (0.00-6.82) | 3.90 (0.00-8.35) | 2.73 (0.00-5.78) | 2.27 (0.00-4.66) | 1.00 | 2.65 (0.00-7.19) |
| Model 4^d^ | Categorical HR (95% CI) | 2.46 (0.00-5.63) | 3.23 (0.00-6.90) | 2.27 (0.00-4.82) | 2.25 (0.00-4.62) | 1.00 | 2.49 (0.00-6.74) |
| Original measurements | |  |  |  |  |  |  |
| Model 1^a^ | Categorical HR (95% CI) | 3.00 (0.00-6.83) | 3.97 (0.00-8.46) | 2.69 (0.00-5.69) | 2.27 (0.00-4.67) | 1.00 | 2.58 (0.00-6.99) |
| Model 2^b^ | Categorical HR (95% CI) | 3.11 (0.00-7.09) | 4.12 (0.00-8.79) | 2.80 (0.00-5.94) | 2.32 (0.00-4.77) | 1.00 | 2.74 (0.00-7.42) |
| Model 3^c^ | Categorical HR (95% CI) | 2.99 (0.00-6.83) | 3.92 (0.00-8.39) | 2.74 (0.00-5.81) | 2.28 (0.00-4.68) | 1.00 | 2.67 (0.00-7.23) |
| Model 4^d^ | Categorical HR (95% CI) | 2.48 (0.00-5.68) | 3.26 (0.00-6.96) | 2.29 (0.00-4.86) | 2.26 (0.00-4.66) | 1.00 | 2.50 (0.00-6.78) |

Categories are based on the Institute of Medicine report 2011 used cut-off values. Category characteristics refer to standardized 25-hydroxyvitamin D concentrations. Abbreviations: HR = Hazard ratio with 95% confidence interval (CI); *The median is the median standardized 25-hydroxyvitamin D value of each category. ^a^Adjusted for age, sex, and season of blood drawing. ^b^Adjusted for age, sex, season of blood drawing, and body mass index (BMI). ^c^Adjusted for age, sex, season of blood drawing, BMI, diabetes mellitus at baseline, and arterial hypertension at baseline. ^d^Adjusted for age, sex, season of blood drawing, BMI, active smoker status, history of cardiovascular disease (CVD), and history of cancer. History of CVD was defined as history of myocardial infarction and/or history of stroke.

# Supplementary Table Q. Adjusted Hazard Ratio of Death From All Causes (95% CI) according to Standardized 25-Hydroxyvitamin D Concentrations in nmol/L in Subgroup Analysis.

|  | Category |  | <30 | 30-39.99 | 40-49.99 | 50-74.99 | 75-99.99 | 100-124.99 | ≥125 |
| --- | --- | --- | --- | --- | --- | --- | --- | --- | --- |
|  | Median*, nmol/L |  | 23.0 | 35.9 | 45.3 | 60.4 | 83.6 | 107.2 | 135 |
|  | Sample size, n |  | 2951 | 3106 | 5018 | 11865 | 3125 | 679 | 172 |
|  | Deaths, n |  | 999 | 892 | 1386 | 2935 | 522 | 57 | 11 |
| Model 2^a^ | Subgroups of sex ^a^ | Female (n=15616) | 1.55 (1.23-1.87) | 1.27 (1.01-1.53) | 1.11 (0.89-1.32) | 1.02 (0.83-1.20) | 1.00 | 0.94 (0.42-1.46) | 0.89 (0.00-2.12) |
|  |  | Male (n=11300) | 1.78 (1.45-2.11) | 1.38 (1.13-1.63) | 1.18 (0.98-1.38) | 1.08 (0.91-1.24) | 1.00 | 1.11 (0.64-1.58) | 1.00 (0.11-1.88) |
| Model 2^b^ | Subgroups of age ^b^ | <60 yrs (n=12214) | 2.29 (1.27-3.31) | 1.38 (0.76-2.01) | 1.26 (0.74-1.77) | 1.18 (0.73-1.64) | 1.00 | 0.76 (0.02-1.50) | 0.94 (0.00-2.49) |
|  |  | 60 to <70 yrs (n=6483) | 2.05 (1.45-2.64) | 1.34 (0.97-1.72) | 1.17 (0.88-1.47) | 1.02 (0.78-1.26) | 1.00 | 1.02 (0.40-1.63) | 0.70 (0.00-1.57) |
|  |  | ≥70 yrs (n=8219) | 1.89 (1.56-2.22) | 1.41 (1.16-1.66) | 1.17 (0.97-1.37) | 1.06 (0.90-1.22) | 1.00 | 1.04 (0.54-1.54) | 1.08 (0.00-2.58) |
| Model 2^c^ | Subgroups of BMI ^c^ | <25 kg/m² (n=11008) | 1.84 (1.45-2.24) | 1.38 (1.08-1.68) | 1.12 (0.90-1.34) | 1.03 (0.85-1.21) | 1.00 | 1.19 (0.61-1.76) | 0.51 (0.00-1.37) |
|  |  | 25 to <30 kg/m² (n=11026) | 1.54 (1.22-1.87) | 1.37 (1.09-1.65) | 1.25 (1.01-1.49) | 1.11 (0.92-1.31) | 1.00 | 0.73 (0.30-1.17) | 1.24 (0.02-2.46) |
|  |  | ≥30 kg/m² (n=4882) | 1.55 (1.01-2.04) | 1.16 (0.80-1.52) | 0.95 (0.66-1.24) | 0.94 (0.67-1.22) | 1.00 | 1.23 (0.30-2.16) | 1.18 (0.00-2.81) |
| Model 2^d^ | Subgroups of intake of calcium ^d^ | No intake (n=15254) | 1.87 (1.52-2.22) | 1.43 (1.16-1.70) | 1.24 (1.02-1.47) | 1.14 (0.95-1.32) | 1.00 | 1.09 (0.50-1.68) | 1.35 (0.00-3.08) |
|  |  | Intake (n=2071) | 1.20 (0.47-1.92) | 1.06 (0.53-1.59) | 0.97 (0.56-1.38) | 0.92 (0.61-1.23) | 1.00 | 0.87 (0.00-1.81) | N.A. |
| Model 2^e^ | Subgroups of intake of vitamin D ^e^ | No intake (n=8810) | 2.32 (1.39-3.24) | 1.59 (0.95-2.24) | 1.38 (0.83-1.93) | 1.26 (0.77-1.75) | 1.00 | 0.81 (0.00-1.79) | 1.09 (0.00-3.12) |
|  |  | Intake  (n=7890) | 1.46 (0.92-2.00) | 1.42 (1.00-1.84) | 1.14 (0.85-1.43) | 1.01 (0.81-1.22) | 1.00 | 1.12 (0.33-1.91) | 0.99 (0.00-4.05) |
| Model 2^f^ | Subgroups of history of CVD ^f^ | No history  (n=20934) | 1.54 (1.29-1.79) | 1.28 (1.08-1.48) | 1.09 (0.93-1.25) | 1.01 (0.87-1.15) | 1.00 | 0.95 (0.56-1.34) | 1.02 (1.12-1.93) |
|  |  | History  (n=3376) | 2.12 (1.53-2.72) | 1.62 (1.15-2.08) | 1.52 (1.09-1.95) | 1.29 (0.95-1.62) | 1.00 | 1.34 (0.42-2.26) | 0.90 (0.00-2.20) |
|  | Category |  | <30 | 30-39.99 | 40-49.99 | 50-74.99 | 75-99.99 | 100-124.99 | ≥125 |
|  | Median*, nmol/L |  | 23.0 | 35.9 | 45.3 | 60.4 | 83.6 | 107.2 | 135 |
|  | Sample size, n |  | 2951 | 3106 | 5018 | 11865 | 3125 | 679 | 172 |
|  | Deaths, n |  | 999 | 892 | 1386 | 2935 | 522 | 57 | 11 |
| Model 2^g^ | Subgroups of history of cancer ^g^ | No history  (n=22260) | 1.70 (1.44-1.96) | 1.33(1.13-1.53) | 1.15 (0.98-1.31) | 1.06 (0.92-1.20) | 1.00 | 1.03 (0.64-1.42) | 1.05 (0.22-1.88) |
|  |  | History  (n=1992) | 1.53 (0.98-2.08) | 1.36 (0.88-1.85) | 1.16 (0.77-1.56) | 0.98 (0.68-1.28) | 1.00 | 0.92 (0.01-1.82) | 0.54 (0.00-1.90) |
| Model 2^h^ | Sensitivity analysis ^h^ | >365 ds (n=26604) | 1.65 (1.42-1.87) | 1.33 (1.15-1.51) | 1.13 (0.99-1.28) | 1.04 (0.92-1.16) | 1.00 | 1.04 (0.69-1.39) | 1.04 (0.29-1.79) |
| Model 2^i^ | Sensitivity analysis ^i^ | >1095 ds  (n=25917) | 1.61 (1.37-1.85) | 1.32 (1.13-1.51) | 1.15 (0.99-1.30) | 1.04 (0.91-1.17) | 1.00 | 1.10 (0.70-1.50) | 1.24 (0.35-2.13) |
| Model 2^j^ | Sensitivity analysis ^j^ | General population  (n=23617) | 1.43 (1.21-1.66) | 1.33 (1.13-1.52) | 1.11 (0.96-1.26) | 1.03 (0.90-1.15) | 1.00 | 1.03 (0.90-1.15) | 1.13 (0.72-1.54) |

Statistical approach was based on categorical models adjusted for age, sex, season of blood drawing, and BMI (i.e. Model 2). Categories are based on the Institute of Medicine report 2011 used cut-off values. Abbreviations: HR = Hazard ratio with 95% confidence interval (CI); yrs = Years; kg/m² = Kilogram per meter squared; ds = Days; BMI = Body mass index; CVD = Cardiovascular disease. ^a^Subgroup comparison of female versus male sex. ^b^Subgroup comparison of three categories of age: <60 yrs, 60 to <70 yrs and ≥70 yrs. ^c^Subgroup comparison of three categories of BMI defined by World Health Organization BMI categories: normal or underweight (<25 kg/m²), overweight (25 to <30 kg/m²); obesity (≥30 kg/m²). ^d^Subgroup comparison of categories of no intake of calcium supplementation versus positive intake of calcium supplementation. ^e^Comparison of categories of no intake of vitamin D supplementation versus positive intake of vitamin D supplementation. ^f^Subgroup comparison of categories of no history of CVD versus positive history of CVD. History of CVD was defined as history of myocardial infarction and/or history of stroke. ^g^Subgroup comparison of categories of no history of cancer versus positive history of cancer. ^h^Sensitivity analysis restricted to participants who died >1 year after baseline examination. ^i^Sensitivity analysis restricted to participants who died >3 yrs after baseline examination. ^j^Sensitivity analysis restricted to general population cohorts (i.e. all cohorts except LURIC).

**18. Prisma Checklist**

| **Section/topic** | **#** | **Checklist item** | **Reported on page #** |
| --- | --- | --- | --- |
| **TITLE** | | |  |
| Title | 1 | Identify the report as a systematic review, meta-analysis, or both. | 1 |
| **ABSTRACT** | | |  |
| Structured summary | 2 | Provide a structured summary including, as applicable: background; objectives; data sources; study eligibility criteria, participants, and interventions; study appraisal and synthesis methods; results; limitations; conclusions and implications of key findings; systematic review registration number. | 5 |
| **INTRODUCTION** | | |  |
| Rationale | 3 | Describe the rationale for the review in the context of what is already known. | 8 |
| Objectives | 4 | Provide an explicit statement of questions being addressed with reference to participants, interventions, comparisons, outcomes, and study design (PICOS). | 8 |
| **METHODS** | | |  |
| Protocol and registration | 5 | Indicate if a review protocol exists, if and where it can be accessed (e.g., Web address), and, if available, provide registration information including registration number. | 9 |
| Eligibility criteria | 6 | Specify study characteristics (e.g., PICOS, length of follow-up) and report characteristics (e.g., years considered, language, publication status) used as criteria for eligibility, giving rationale. | 9,11, appendix, section 1 |
| Information sources | 7 | Describe all information sources (e.g., databases with dates of coverage, contact with study authors to identify additional studies) in the search and date last searched. | 9, appendix, section 1 |
| Search | 8 | Present full electronic search strategy for at least one database, including any limits used, such that it could be repeated. | 9, appendix, section 1 |
| Study selection | 9 | State the process for selecting studies (i.e., screening, eligibility, included in systematic review, and, if applicable, included in the meta-analysis). | 9,10, appendix, section 1 |
| Data collection process | 10 | Describe method of data extraction from reports (e.g., piloted forms, independently, in duplicate) and any processes for obtaining and confirming data from investigators. | Appendix, section 3 |
| Data items | 11 | List and define all variables for which data were sought (e.g., PICOS, funding sources) and any assumptions and simplifications made. | Appendix, section 4,5 |
| Risk of bias in individual studies | 12 | Describe methods used for assessing risk of bias of individual studies (including specification of whether this was done at the study or outcome level), and how this information is to be used in any data synthesis. | Appendix, section 9 |
| Summary measures | 13 | State the principal summary measures (e.g., risk ratio, difference in means). | 11 |
| Synthesis of results | 14 | Describe the methods of handling data and combining results of studies, if done, including measures of consistency (e.g., I^2^) for each meta-analysis. | 13, appendix, section 9 |

| **Section/topic** | **#** | **Checklist item** | **Reported on page #** |
| --- | --- | --- | --- |
| Risk of bias across studies | 15 | Specify any assessment of risk of bias that may affect the cumulative evidence (e.g., publication bias, selective reporting within studies). | Appendix section 9 |
| Additional analyses | 16 | Describe methods of additional analyses (e.g., sensitivity or subgroup analyses, meta-regression), if done, indicating which were pre-specified. | 12,13 |
| **RESULTS** | | |  |
| Study selection | 17 | Give numbers of studies screened, assessed for eligibility, and included in the review, with reasons for exclusions at each stage, ideally with a flow diagram. | 9, and appendix, section 1 |
| Study characteristics | 18 | For each study, present characteristics for which data were extracted (e.g., study size, PICOS, follow-up period) and provide the citations. | 15, and appendix section 2 |
| Risk of bias within studies | 19 | Present data on risk of bias of each study and, if available, any outcome level assessment (see item 12). | appendix section 2 and 9 |
| Results of individual studies | 20 | For all outcomes considered (benefits or harms), present, for each study: (a) simple summary data for each intervention group (b) effect estimates and confidence intervals, ideally with a forest plot. | Reported in supplementary tables |
| Synthesis of results | 21 | Present results of each meta-analysis done, including confidence intervals and measures of consistency. | 15 |
| Risk of bias across studies | 22 | Present results of any assessment of risk of bias across studies (see Item 15). | Appendix section 9 |
| Additional analysis | 23 | Give results of additional analyses, if done (e.g., sensitivity or subgroup analyses, meta-regression [see Item 16]). | 15 |
| **DISCUSSION** | | |  |
| Summary of evidence | 24 | Summarize the main findings including the strength of evidence for each main outcome; consider their relevance to key groups (e.g., healthcare providers, users, and policy makers). | 15 |
| Limitations | 25 | Discuss limitations at study and outcome level (e.g., risk of bias), and at review-level (e.g., incomplete retrieval of identified research, reporting bias). | 18 |
| Conclusions | 26 | Provide a general interpretation of the results in the context of other evidence, and implications for future research. | 18 |
| **FUNDING** | | |  |
| Funding | 27 | Describe sources of funding for the systematic review and other support (e.g., supply of data); role of funders for the systematic review. | 7, appendix section 11 |

*From:*  Moher D, Liberati A, Tetzlaff J, Altman DG, The PRISMA Group (2009). Preferred Reporting Items for Systematic Reviews and Meta-Analyses: The PRISMA Statement. PLoS Med 6(7): e1000097. doi:10.1371/journal.pmed1000097

For more information, visit: **www.prisma-statement.org**.

**19. Meta-analysis flow chart**


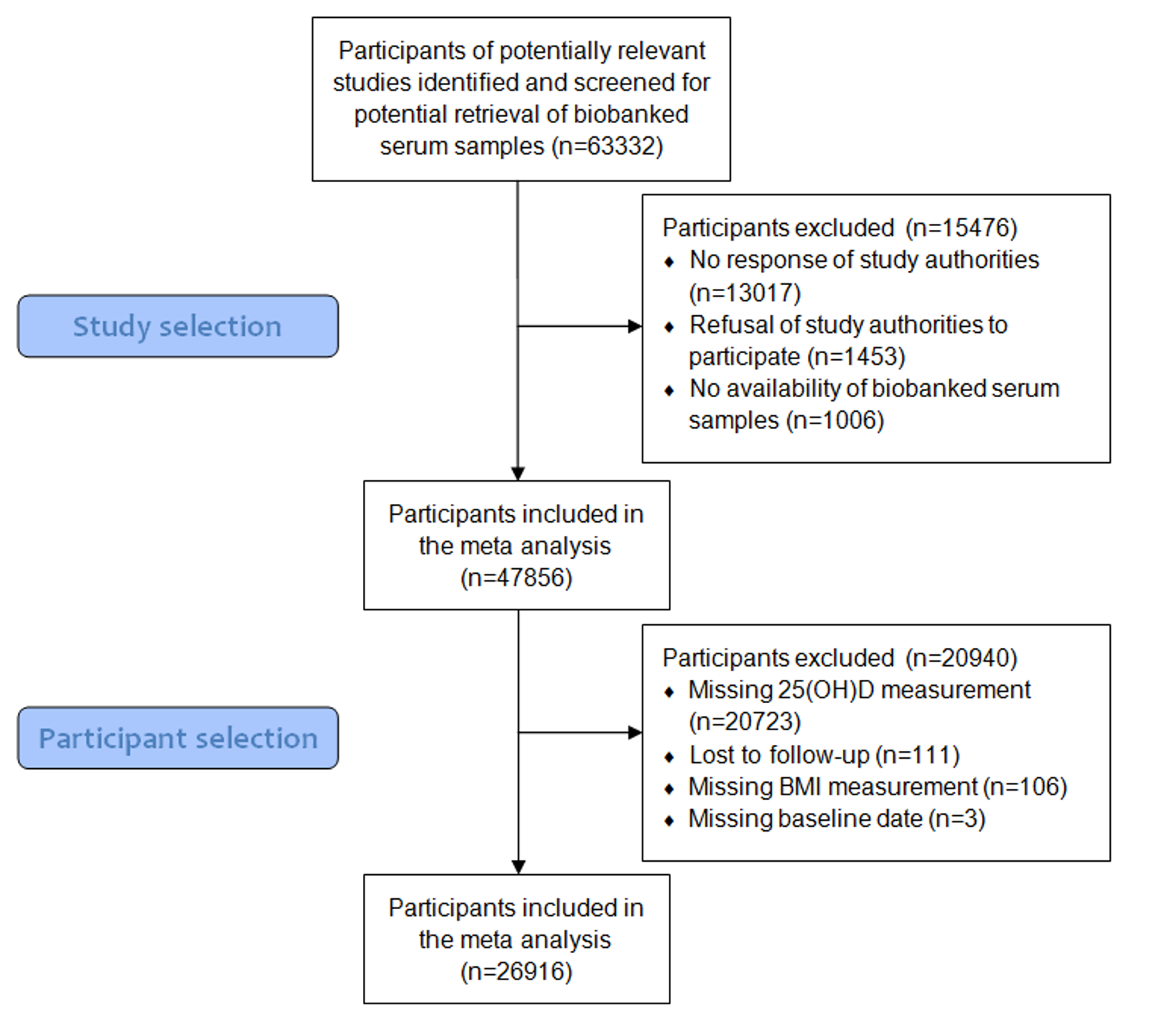

Supplement: S1 File — (DOC) [file pone.0170791.s001.doc]
